# Abemaciclib in combination with pembrolizumab for HR+, HER2− metastatic breast cancer: Phase 1b study

**DOI:** 10.1038/s41523-022-00482-2

**Published:** 2022-11-05

**Authors:** Hope S. Rugo, Peter Kabos, J. Thad Beck, Guy Jerusalem, Hans Wildiers, Elena Sevillano, Luis Paz-Ares, Michael J. Chisamore, Sonya C. Chapman, Anwar M. Hossain, Yanyun Chen, Sara M. Tolaney

**Affiliations:** 1 University of California San Francisco Helen Diller Family Comprehensive Cancer Center, San Francisco, CA USA; 2 Divisions of Medical Oncology, Department of Medicine, University of Colorado, Anschutz Medical Campus, 12801 East 17th Avenue, Aurora, CO 80045 USA; 3 Highlands Oncology Group, Fayetteville, AR USA; 4 Laboratory of Medical Oncology, University of Liège, Liège, Belgium; 5 Department of Medical Oncology, CHU Sart-Tilman Liège, Liège, Belgium; 6 Department of General Medical Oncology, University Hospitals Leuven, Leuven, Belgium; 7 Department of Medical Oncology, Centro Integral Oncologico Clara Campal, Madrid, Spain; 8 Hospital Universitario 12 de Octubre, CNIO-H120 Lung Cancer Unit, Universidad Complutense and Ciberonc, Madrid, Spain; 9 Department of Clinical Research, Merck & Co., Inc., Rahway, NJ USA; 10 Eli Lilly and Company, Bracknell, UK; 11 Eli Lilly and Company, Indianapolis, IN USA; 12 Department of Medical Oncology, Dana‐Farber Cancer Institute, Boston, MA USA

**Keywords:** Breast cancer, Cancer immunotherapy, Targeted therapies

## Abstract

This nonrandomized, open-label, multi-cohort Phase 1b study (NCT02779751) investigated the safety and efficacy of abemaciclib plus pembrolizumab with/without anastrozole in patients with hormone receptor-positive (HR+), human epidermal growth factor receptor 2-negative (HER2−) metastatic breast cancer (MBC) without prior CDK4 and 6 inhibitor exposure. Patients were divided into two cohorts: treatment naïve (cohort 1) and pretreated (cohort 2). Patients received abemaciclib plus pembrolizumab with (cohort 1) or without (cohort 2) anastrozole over 21-day cycles. The primary objective was safety, and secondary objectives included efficacy and pharmacokinetics (PK). Cohort 1/2 enrolled 26/28 patients, respectively. Neutropenia (30.8/28.6%), AST increase (34.6/17.9%), ALT increase (42.3/10.7%), and diarrhea (3.8/10.7%) were the most frequent grade ≥3 adverse events in cohort 1/2, respectively. A total of two deaths occurred, which investigators attributed to treatment-related adverse events (AEs), both in cohort 1. Higher rates of all grade and grade ≥3 interstitial lung disease (ILD)/pneumonitis were observed compared to previously reported with abemaciclib and pembrolizumab monotherapy. The PK profiles were consistent between cohorts and with previous monotherapy studies. In cohorts 1/2, the overall response rate and disease control rate were 23.1/28.6% and 84.6/82.1%, respectively. Median progression-free survival and overall survivals were 8.9 (95% CI: 3.9–11.1) and 26.3 months (95% CI: 20.0–31.0) for cohort 2; cohort 1 data are immature. Abemaciclib plus pembrolizumab demonstrated antitumor activity, but high rates of ILD/pneumonitis and severe transaminase elevations occurred with/without anastrozole compared to the previous reporting. Benefit/risk analysis does not support further evaluation of this combination in the treatment of HR+, HER2− MBC.

## Introduction

Cell cycle dysregulation and uncontrolled proliferation are hallmarks of cancer. Constitutive or deregulated activity of cyclin-dependent kinases (CDK) often drives rapid progression through the cell cycle^1^. CDK4 and 6 participate in a complex with D-type cyclins, phosphorylating the retinoblastoma tumor suppressor protein, and thereby initiating transition through the G1 phase of the cell cycle^2^. In hormone receptor-positive (HR+) breast cancer, estrogen stimulates the expression of cyclin D1, contributing to uncontrolled cell growth^3^. Because of their key role in cell cycle regulation and implication in carcinogenesis, cell cycle regulatory proteins represent attractive drug targets for cancer intervention. The development of CDK4 and 6 inhibitors has changed the therapeutic management of HR+, human epidermal growth factor receptor 2-negative (HER2−) metastatic breast cancer (MBC).

Abemaciclib is a small molecule CDK4 and 6 inhibitors administered twice-daily (BID) on a continuous schedule. Abemaciclib has received global approval for the treatment of HR+, HER2− MBC in combination with endocrine therapy (ET)^4–7^ and as a monotherapy^8^. MONARCH 3 was a randomized, double-blind Phase 3 study of abemaciclib 150 mg BID plus a nonsteroidal aromatase inhibitor (NSAI) as initial therapy in women with HR+, HER2− MBC^7^. Abemaciclib plus NSAI significantly improved progression-free survival (PFS, median 28.2 vs 14.8 months; hazard ratio [HR]:0.540; 95% confidence interval [CI] 0.418, 0.698); *P* = 0.000002). In MONARCH 1, abemaciclib 200 mg BID monotherapy in patients with refractory HR+, HER2− MBC, demonstrated an objective response rate (ORR) of 19.7%^8^. The results of MONARCH 3 and MONARCH 1 studies form the basis for abemaciclib approval in HR+, HER2− MBC plus NSAIs, and as a monotherapy, respectively^9^.

In addition to cell cycle deregulation, cancer cells have the ability to evade immune surveillance. One such approach is through the programmed death-1 (PD-1) pathway, where cancer cells expressing programmed death-ligand 1 (PD-L1) interact with PD-1-expressing T cells to inhibit immune detection and response^10^. Thus, inhibition of PD-1 and PD-L1 can lead to the reactivation of antitumor T-cell response.

Pembrolizumab is a highly selective, humanized immunoglobulin G4 (IgG4) monoclonal antibody targeting the PD-1 receptor. Pembrolizumab has demonstrated robust antitumor activity and is FDA-approved in combination with chemotherapy for the treatment of PD-L1-positive metastatic triple-negative breast cancer (TNBC). Response to immunotherapy has been conflicting in HR+/HER2− breast cancer. For example, in the KEYNOTE-028 study, single-agent pembrolizumab exhibited modest activity (objective response rate, ORR = 12%) in a subset of patients with PD-L1-positive, HR+, HER2− MBC^11^; yet in the SPY2 trial, pembrolizumab combined with neoadjuvant chemotherapy in early HER2− breast cancer resulted in significantly higher predicted pCR rate in both HR+ disease and TNBC compared with chemotherapy alone^12^.

In preclinical models, abemaciclib monotherapy increased tumor immunogenicity^13^, and synergized PD-1 blockade to enhance antitumor efficacy^14,15^. In the neoMONARCH study, abemaciclib plus anastrozole demonstrated biological and clinical activity in patients with stage I–IIIB HR+, HER2− breast cancer in the neoadjuvant setting^16^. This combination resulted in an increased adaptive immune response indicative of enhanced antigen presentation and activated T-cell phenotypes^16^. Preclinical and preliminary clinical data support the investigation of whether the combination of CDK4 and 6 inhibitors with anti-PD-1/PD-L1 could provide improved clinical benefits beyond what is observed with the current treatment options available for patients with HR+, HER2− MBC.

In an early Phase 1b study I3Y-MC-JPBJ Part E, which enrolled and treated 20 patients with stage IV non-small cell lung cancer (NSCLC) with abemaciclib and pembrolizumab, the maximum tolerated dose of abemaciclib (150 mg BID) and pembrolizumab (200 mg infused intravenously [iv] on day 1 of 21-day cycles) was determined. The safety profile for Part E of the study JPBJ was consistent with the known safety profile of the individual treatment components, abemaciclib, and pembrolizumab, in the setting of advanced NSCLC. The current study was designed to further evaluate the safety and preliminary anticancer activity of this combination in patients with stage IV NSCLC or HR+, HER2− MBC.

Here we report safety, efficacy, and pharmacokinetic (PK) results from a Phase 1b study of the combination of abemaciclib with pembrolizumab plus anastrozole as initial treatment in treatment-naive HR+, HER2− MBC patients (cohort 1) or in pretreated HR+, HER2− MBC without anastrozole (cohort 2). The results of the NSCLC cohorts will be reported separately.

## Results

### Patient demographics, treatment, and disposition

Between November 2016 and August 2019, 54 patients were enrolled in cohort 1 and 2 of this study. Twenty-six patients from two different countries (*n* = 10 Belgium; *n* = 16 United States) were enrolled in cohort 1 and received treatment with abemaciclib in combination with pembrolizumab and anastrozole. Twenty-eight patients from six different countries/regions (*n* = 1 Taiwan; *n* = 2 Belgium; *n* = 6 Spain; *n* = 1 France; *n* = 2 Italy; *n* = 16 USA) were enrolled in cohort 2 and received treatment with abemaciclib plus pembrolizumab. Baseline patient demographics and disease characteristics for each cohort are outlined in Table 1. The patients in cohort 2 received a median of three lines (range 1–7) of prior systemic therapy in the metastatic setting (Table 2).

**Table 1:** Baseline patient demographics and disease characteristics.

Characteristics	Cohort 1 *N* = 26	Cohort 2 *N* = 28
Sex, *n* (%)		
Female	26 (100.0)	27 (96.4)
Male	—	1 (3.6)
Age, years, median (range)	58 (34–79)	55 (31–76)
Race, *n* (%)		
White	24 (92.3)	25 (89.3)
Asian	2 (7.7)	1 (3.6)
Black or African American	—	1 (3.6)
Not reported	—	1 (3.6)
ECOG PS, *n* (%)		
0	19 (73.1)	19 (67.9)
1	7 (26.9)	9 (32.1)
Nature of disease, *n* (%)		
Visceral^a^	17 (65.4)	23 (82.1)
Bone-only	2 (7.7)	1 (3.6)
Others	7 (26.9)	4 (14.3)
Disease locations, *n* (%)		
Liver	12 (46.2)	18 (64.3)
Lung	6 (23.1)	10 (35.7)
Bone	19 (73.1)	19 (67.9)
Nodal	17 (65.4)	17 (60.7)
Other^b^	5 (19.1)	12 (42.8)
Number of metastatic sites^c^, *n* (%)		
1	5 (19.2)	4 (14.3)
2	7 (26.9)	4 (14.3)
≥3	14 (53.8)	20 (71.4)
PD-L1 status, *n* (%)		
Positive	3 (11.5)	12 (42.9)
Negative	15 (57.7)	12 (42.9)
Unknown	8 (30.8)	4 (14.3)
Transaminase elevations^d^, *n* (%)		
ALT increased	6 (23.1)	5 (18.5)
AST increased	8 (30.7)	7 (26.9)

Enrolled population. Data cutoff: August 19, 2020.

*ALT* alanine aminotransferase, *AST* aspartate aminotransferase, *ECOG PS* Eastern Cooperative Oncology Group performance status, *N* number of randomized patients, *n* number of patients in category, *PD-L1* programmed death-ligand 1.

^a^Included brain, central nervous system (non-brain), liver, lung, peritoneum, pleura, and other visceral sites.

^b^Included soft tissue and other sites (visceral and non-visceral).

^c^Any metastatic site.

^d^Inclusion criterion ≤3.0 × ULN OR ≤ 5 × ULN if the liver had tumor involvement. Note: excludes one missing value for ALT and two missing values for AST in cohort 2.

**Table 2:** Prior anticancer therapy and surgery.

Prior therapy, *n* (%)	Cohort 1 *N* = 26	Cohort 2 *N* = 28
Surgery	18 (69.2)	25 (89.3)
Radiotherapy	14 (53.8)	18 (64.3)
Systemic therapy	13 (50.0)	28 (100.0)
Neoadjuvant	1 (3.8)	7 (25.0)
Adjuvant	13 (50.0)	20 (71.4)
Locally advanced/metastatic	1 (3.8)	28 (100)
Chemotherapy		27 (96.4)^b^
Endocrine therapy	1 (3.8)^a^	25 (89.3)
Targeted therapy		8 (28.6)
Other		7 (25.0)
Median line of systemic therapy for metastatic disease (range)	—	3 (1–7)
Prior lines of chemotherapy for metastatic disease		
1	—	15 (53.6)
2	—	12 (42.9)
Prior lines of endocrine therapy for metastatic disease		
1	1 (3.8)^a^	10 (35.7)
2	—	7 (25.0)
≥3	—	8 (28.6)

*N* number of patients treated, *n* number of patients in category.

^a^One patient in cohort 1 previously received 8 days of NSAI in MBC setting.

^b^One patient in cohort 2 did not receive chemotherapy for metastatic disease but did receive a target therapy (alpelisib).

In cohort 1, the median duration of treatment was 12.3 weeks (range 4.1–97.0 weeks) for abemaciclib, 10.4 weeks (range 3.1–96.1 weeks) for pembrolizumab, and 15.9 weeks (range 4.1–97.0 weeks) for anastrozole. Patients received a median of 3.0 cycles (range 1–32 cycles) of abemaciclib, 2.0 cycles (range 1–32 cycles) of pembrolizumab, and 4.5 cycles (range 1–32 cycles) of anastrozole. In cohort 2, the median duration of treatment was 28.9 weeks (range 3.0–106.0 weeks) for abemaciclib and 31.1 weeks (range 4.0–106.0 weeks) for pembrolizumab. Patients received a median of 9.0 cycles (range 1–35 cycles) of abemaciclib and 8.5 cycles (range 1–35 cycles) of pembrolizumab.

At the time of data cutoff (August 19, 2020), 21 (80.8%) patients in cohort 1 and all patients (*n* = 28; 100.0%) in cohort 2 had discontinued treatment. The main reason for treatment discontinuation was progressive disease (*n* = 9; 34.6%) or adverse events (*n* = 8; 30.8%) for cohort 1 and progressive disease (*n* = 19, 67.9%) or adverse events (*n* = 5, 17.9%) for cohort 2.

### Safety

An overview of safety by cohort is presented in Table 3. In cohort 1, 9 (34.6%) patients discontinued treatment due to an AE: 7 (26.9%) due to increased ALT, 1 (3.8%) due to hyperthyroidism, and 1 (3.8%) due to ILD. In cohort 2, 6 (21.4%) patients discontinued study treatment due to an AE: 3 (10.7%) due to increased ALT, 1 (3.6%) due to acute kidney injury, 1 (3.6%) blood creatinine increase, and 1 (3.6%) due to sepsis. Nine patients in cohort 1 (34.6%) and 10 patients in cohort 2 (35.7%) had ≥1 SAE; of those, 7 (26.9%) and 6 (21.4%) were deemed related to treatment, respectively. Two patients in cohort 1 and one patient in cohort 2 died due to an AE while on study treatment or within 30 days of treatment discontinuation. Of the two deaths that occurred in cohort 1 due to an AE; one was due to ILD while on study treatment, and the other was due to acute hypoxic respiratory failure (within 30 days of treatment discontinuation) secondary to an ongoing treatment-related AE of grade 3 ILD. Both deaths were assessed as related to the study treatment. The patient from cohort 2 died due to sepsis, which was investigator-assessed and not considered to be related to the study treatment.

**Table 3:** Safety overview.

Number of patients^a^, *n* (%)	Cohort 1, (*N* = 26)	Cohort 2, (*N* = 28)
Patients with ≥1 TEAE	26 (100)	27 (96.4)
Related to study treatment^b^	26 (100)	27 (96.4)
Patients with ≥1 grade ≥3 TEAE	18 (69.2)	17 (60.7)
Related to study treatment^b^	18 (69.2)	15 (53.6)
Patients with ≥1 SAE	9 (34.6)	10 (35.7)
Related to study treatment^b^	7 (26.9)	6 (21.4)
Patients who discontinued study treatment regimen due to AE	9 (34.6)	6 (21.4)
Related to study treatment^b^	9 (34.6)	5 (17.9)
Patients who died due to AE on study treatment or within 30 days of discontinuation^c^	2 (7.7)	1 (3.6)
Related to study treatment^b^	2 (7.7)	0

*AE* adverse event, *N* number of treated patients, *n* number of patients in category, *SAE* serious adverse event, *TEAE* treatment-emergent adverse event.

^a^Subjects may be counted in more than one category.

^b^Events that were considered related to study treatment as judged by the investigator.

^c^Of the two patients that died in cohort 1, one was due to ILD and the other due to acute hypoxic respiratory failure secondary to ILD. The patient from cohort 2 died due to sepsis.

In cohort 1, any grade TEAEs were experienced by all patients (*n* = 26; 100.0%). The most frequent were diarrhea (*n* = 22; 84.6%), ALT increased (*n* = 14; 53.8%), fatigue (*n* = 13; 50.0%), and AST increased (*n* = 12; 46.2%) (Table 4). Grade ≥3 TEAEs were reported by 18 (69.2%) patients, the most common included ALT increased (*n* = 11, 42.3%), AST increased (*n* = 9, 34.6%), and neutropenia (*n* = 8, 30.8%). The overall incidence of all-grade ILD/pneumonitis was 11.5% (*n* = 3), and grade ≥3 was 7.7% (*n* = 2, one each of grade 3 and grade 5); the grade 3 ILD event was later reported to lead to acute hypoxic respiratory failure after treatment discontinuation, which resulted in death (Table 4).

**Table 4:** Treatment-emergent adverse events (all-causality) occurring in ≥25% of patients who received ≥1 dose of study treatment (in either cohort, any grade).

MedDRA preferred term	Cohort 1, (*N* = 26), *n* (%)		Cohort 2, (*N* = 28), *n* (%)	
	Any grade	Grade ≥ 3	Any grade	Grade ≥ 3
*Patients with* *≥* *1 TEAE*	26 (100.0)	18 (69.2)	27 (96.4)	17 (60.7)
Diarrhea	22 (84.6)	1 (3.8)	22 (78.6)	3 (10.7)
Fatigue	13 (50.0)	0 (0.0)	15 (53.6)	0 (0.0)
Headache	2 (7.7)	0 (0.0)	14 (50.0)	0 (0.0)
Neutropenia	10 (38.5)	8 (30.8)	14 (50.0)	8 (28.6)
Cough	6 (23.1)	0 (0.0)	12 (42.9)	0 (0.0)
Abdominal pain	6 (23.1)	0 (0.0)	11 (39.3)	1 (3.6)
Nausea	10 (38.5)	0 (0.0)	11 (39.3)	1 (3.6)
Pruritus	7 (26.9)	1 (3.8)	11 (39.3)	0 (0.0)
Decreased appetite	6 (23.1)	0 (0.0)	10 (35.7)	0 (0.0)
AST increased	12 (46.2)	9 (34.6)	9 (32.1)	5 (17.9)
Vomiting	6 (23.1)	0 (0.0)	9 (32.1)	1 (3.6)
Dyspnea	8 (30.8)	1 (3.8)	8 (28.6)	1 (3.6)
ALT increased	14 (53.8)	11 (42.3)	7 (25.0)	3 (10.7)
Anemia	5 (19.2)	2 (7.7)	7 (25.0)	1 (3.6)
Arthralgia	4 (15.4)	0 (0.0)	7 (25.0)	0 (0.0)
Rash	5 (19.2)	1 (3.8)	7 (25.0)	1 (3.6)
ILD/pneumonitis^a^	3 (11.5)	2 (7.7)	4 (14.3)	1 (3.6)

*ALT* alanine aminotransferase, *AST* aspartate aminotransferase, *ILD* interstitial lung disease, *N* number of treated patients, *n* number of patients in category, *TEAE* treatment-emergent adverse event.

^a^These events are adverse events of interest.

In cohort 1, abemaciclib, pembrolizumab, and anastrozole dose adjustments occurred for 21 (80.8%), 18 (69.2%), and 8 (30.8%) patients, respectively. Abemaciclib dose adjustments included dose reductions (*n* = 10, 38.5%) and dose omissions (*n* = 19, 73.1%). Pembrolizumab dose adjustments included dose delays (*n* = 6, 23.1%) and dose omissions (*n* = 15, 57.7%). Anastrozole dose adjustments were due to dose omissions (*n* = 8, 30.8%).

In cohort 2, all-grade TEAEs were reported by 27 (96.4%) patients. The most frequent TEAEs were diarrhea (*n* = 22; 78.6%), fatigue (*n* = 15; 53.6%), headache (*n* = 14; 50.0%), and neutropenia (*n* = 14, 50.0%) (Table 4). Grade ≥3 TEAEs were reported by 17 (60.7%) patients, the most common included neutropenia (*n* = 8; 28.6%), AST increased (*n* = 5; 17.9%), diarrhea (*n* = 3; 10.7%), and ALT increased (*n* = 3; 10.7%). The overall incidence of all-grade ILD/pneumonitis was 14.3% (*n* = 4); 1 (3.6%) was a grade 3 event; no grade 4 or 5 events (Table 4).

In cohort 2, abemaciclib and pembrolizumab dose adjustments occurred for 17 (60.7%) and 16 (57.1%) patients, respectively. Abemaciclib dose adjustments included dose reductions (*n* = 10; 35.7%) and dose omissions (*n* = 17; 60.7%). Pembrolizumab dose adjustments included dose delays (*n* = 10; 35.7%) and dose omissions (*n* = 11; 39.3%); there were no dose reductions for pembrolizumab.

#### Anticancer activity

At the time of data cutoff, the median follow-up times were 16.4 months and 39.9 months for cohorts 1 and 2, respectively.

**Fig. 1:**
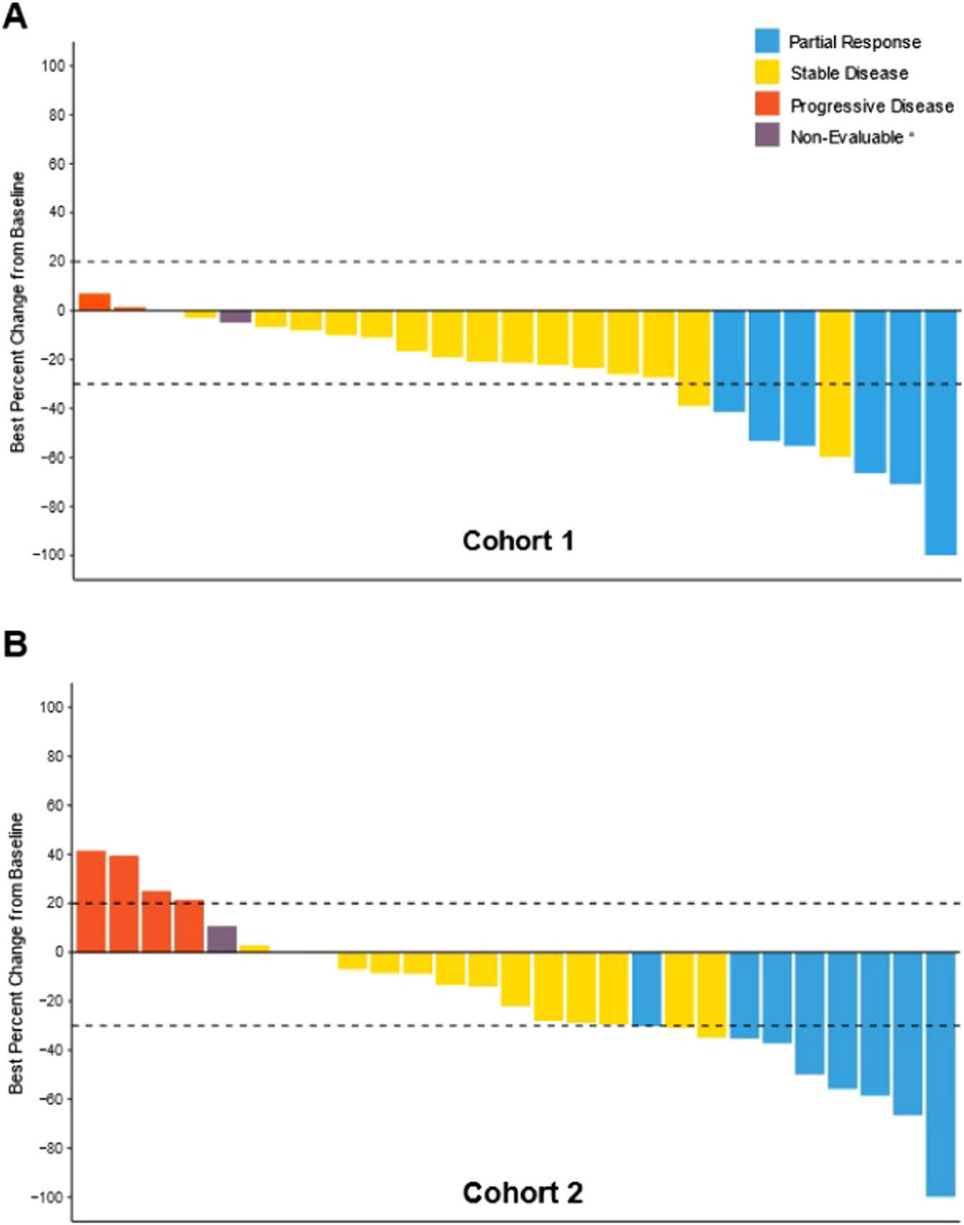
Best percent change in tumor size from baseline according to RECIST 1.1. Best percent change in tumor size from baseline is presented for the safety populations in cohort 1 (**A**) and cohort 2 (**B**). Best overall responses presented here are confirmed responses.^a^Patients without any post baseline data are not included in the graphs.

In cohort 1, 6 (23.1%) patients had a PR, 16 (61.5%) had SD, with 4 (15.4%) patients having persistent SD for ≥6 months. ORR, CBR, and DCR were 23.1%, 38.5% and 84.6%, respectively (Table 5). Median DoR for the 6 patients who had a PR was non-estimable (4 of 6 patients censored). Median PFS and OS were not reached (Supplementary Fig. 3). The best percent change in tumor size according to RECIST 1.1 criteria for evaluable patients in cohort 1 is shown in Fig. 1A.

**Table 5:** Summary of antitumor activity (safety population).

	Cohort 1, (*N* = 26), *n* (%)		Cohort 2, (*N* = 28), *n* (%)	
	*n* (%)	95% CI^a^	*n* (%)	95% CI^a^
Best overall response				
Complete response (CR)	0	NA	0	NA
Partial response (PR)	6 (23.1)	9.0–43.7	8 (28.6)	13.2–48.7
Stable disease (SD)	16 (61.5)	40.6–79.8	15 (53.6)	33.9–72.5
Persistent SD^b^	4 (15.4)	4.4–34.9	5 (17.9)	6.1–36.9
Objective PD	3 (11.5)	2.5–30.2	4 (14.3)	4.0–32.7
Non-evaluable	1 (3.8)	0.1–19.6	1 (3.6)	0.1–18.4
ORR (CR + PR)	6 (23.1)	9.0–43.7	8 (28.6)	13.2–48.7
DCR (CR + PR + SD)	22 (84.6)	65.1–95.6	23 (82.1)	63.1–93.9
CBR (CR + PR + persistent SD^b^)	10 (38.5)	20.2–59.4	13 (46.4)	27.5–66.1
PFS, median mos (95% CI)	NR		8.9 (3.9, 11.1)	
OS, median mos (95% CI)	NR		26.3 (20.0, 31.0)	

*CBR* clinical benefit rate, *CI* confidence interval, *CR* complete response, *DCR* disease control rate, *mos* months, *N* number patients in safety population, *n* number of patients in category, *NA* not applicable, *NR* not reached, *ORR* overall response rate, *OS* overall survival, *PD* progressive disease, *PFS* progression-free survival, *PR* partial response, *SD* stable disease.

^a^Confidence intervals based on the Clopper-Pearson method.

^b^Stable disease that persisted for ≥6 months.

In cohort 2, 8 (28.6%) patients had a PR and 15 (53.6%) patients had SD (Table 5). In 5 (17.9%) patients, SD persisted for a period of ≥6 months. ORR, CBR, and DCR were 28.6%, 46.4%, and 82.1%, respectively (Table 5). Median DoR for the 8 patients who had a PR was 5.2 months (95% CI: 2.8, 8.7). The median PFS and OS were 8.9 months (95% CI: 3.9, 11.1) and 26.3 months (95% CI: 20.0, 31.0), respectively (Supplementary Fig. 3). The best percent change in tumor size according to RECIST 1.1 criteria for treated patients in cohort 2 is shown in Fig. 1B. Baseline PD-L1 status (negative < 1%, positive ≥ 1%) did not appear to be predictive for response to the combination of abemaciclib plus pembrolizumab (Supplementary Fig. 2).

### Pharmacokinetic analyses

PK data were available from all 26 patients in cohort 1 and all 28 patients in cohort 2. The PK of abemaciclib, pembrolizumab, and anastrozole are shown in Supplementary Fig. 1. Mean abemaciclib concentrations decreased slightly over time, indicative of patients with high exposures experiencing dose reductions and thus being excluded from the 150 mg Q12H PK analysis group. In contrast, pembrolizumab exposures increased over time, as expected, due to the long half-life of the monoclonal antibody and extended time to steady-state conditions.

The PK profiles observed for abemaciclib and pembrolizumab were consistent between cohorts 1 and 2. For all three drugs, the PK profiles are consistent with those reported in monotherapy studies. Equally, all three drugs reached systemic concentrations which are known to be clinically efficacious^17–21^. These results support the anticipated lack of pharmacokinetic drug–drug interaction between abemaciclib and pembrolizumab, and abemaciclib, pembrolizumab, and anastrozole in breast cancer.

## Discussion

The data reported here present the results of two cohorts of a Phase 1b study of safety and preliminary clinical efficacy of abemaciclib plus pembrolizumab for patients with HR+, HER2− MBC (with and without anastrozole), including: newly diagnosed patients who had not received any prior systemic anticancer ET or chemotherapy (cohort 1) or patients heavily pretreated (cohort 2). Overall, abemaciclib plus pembrolizumab was associated with higher rates of grade ≥3 transaminase elevations and all-grade ILD/pneumonitis in both cohorts than what has previously been reported for either drug alone.

The safety profile for the triplet therapy in cohort 1 was consistent with individual treatments, except for transaminase elevations and ILD/pneumonitis^6,22^. Abemaciclib and pembrolizumab monotherapy are known to have overlapping toxicities, particularly hepatic events and ILD. In comparison to MONARCH 3 (abemaciclib plus anastrozole or letrozole in a similar patient population), patients in cohort 1 experienced increased incidence of all grades (53.8% vs 17.4%) and grade ≥3 ALT increased (42.3% vs 6.4%); all grades (46.2% vs 16.8%) and grade ≥3 AST increased (34.6% vs 3.7%); and all grades (11.5% vs 5.2%) and grade ≥3 ILD/pneumonitis (7.7% vs 1.2%)^23^. Moreover, higher rates of SAEs and treatment discontinuation due to AEs were observed in cohort 1 when compared to MONARCH 3 (34.6% vs 27.5%, 34.6% vs 16.5%)^6,7^.

When comparing cohort 2 to MONARCH 1 (monotherapy abemaciclib in patients with refractory HR+, HER2− MBC), we observed a similar incidence of all grades ALT increased (25% vs 31%), and AST increased (32.1% vs 30%) and a higher incidence of grade ≥3 increase for both (ALT: 10.7% vs 3%, AST:17.9% vs 4%). The KEYNOTE-028 study, which investigated pembrolizumab monotherapy in patients with estrogen receptor (ER)+, HER2− MBC, reported no transaminase elevations; however, one grade 3 hepatitis event was reported (4%). In addition, a higher incidence of ILD/pneumonitis was observed in cohort 2 (any grade events: 14.3%) compared to values reported across MONARCH 1, MONARCH 2, and MONARCH 3 (any grade events: 3.3% of abemaciclib-treated patients) or KEYNOTE-028 (grade 1 pneumonitis: 4%)^8,9,11^. Furthermore, higher rates of SAEs and treatment discontinuation due to AEs were reported in cohort 2 when compared to MONARCH 1 (35.7% vs 24.2%, 21.4% vs 7.6%)^8^.

In cohort 1, the combination of abemaciclib and pembrolizumab with anastrozole as first-line therapy resulted in a much higher incidence of all grades and grade ≥3 transaminase elevations, grade ≥3 ILD/pneumonitis, and treatment discontinuation due to AEs than in cohort 2 as later line therapy. Of note, this study was conducted prior to the COVID-19 pandemic, and ILD incidence was not impacted by clinically similar COVID-19 symptoms. Anastrozole is known to be associated with transaminase elevations^24^. However, the contribution of anastrozole does not account for the differences in all grades and grade ≥3 transaminase elevations observed between the two cohorts. It should be mentioned that Grade 3/Grade 4 ALT/AST increase was not associated with liver metastases (data not shown). The differences in safety profiles in cohort 1 and 2 were not attributed to differences in PK, as the PK profiles of abemaciclib and pembrolizumab were similar between cohorts. In addition, for all three study drugs, the PK profiles observed in the current study were consistent with the known monotherapy PK data for each study drug^17–21^.

From a risk/benefit perspective, it can be difficult to accurately identify the drug-causing hepatic events as all study drugs are known to be associated with liver test abnormalities. In addition, AEs were found to be a leading cause of treatment discontinuation, and when compared to previous experience with abemaciclib as monotherapy or in combination with ET, a higher rate of treatment discontinuation due to AEs was observed for this study. In cohort 1, ILD/pneumonitis was observed in three out of 26 patients, two of which had a fatal outcome; the triple combination is thus not a tolerable therapy. The NEWFLAME trial, investigating nivolumab plus abemaciclib plus ET (fulvestrant or letrozole) as a first- or second-line treatment for patients with HR+, HER2−, MBC, reported similar safety findings to this study, including high rates of treatment discontinuation, elevated liver function tests, and ILD/pneumonitis^25^.

While cross-trial comparisons must be interpreted with caution, a numerically lower ORR (23.1%; 95% CI: 9.0, 43.7) was observed for cohort 1 when compared to the MONARCH 3 study (55.4%: 95% CI: 53.3, 65.1)^7^. Lower ORR observed in cohort 1 may be explained by higher treatment discontinuation due to AEs attributable to the regimen’s toxicity and short follow-up time. The ORR observed for cohort 2 (28.6%; 95% CI: 13.2, 48.7) compares well with that observed with abemaciclib monotherapy in MONARCH 1 (19.7%; 95% CI: 13.3, 27.5), and with pembrolizumab monotherapy in patients with ER+ HER2− MBC (12%; 95% CI: 2.5, 31.2) (KEYNOTE-028)^8,11^.

In comparison to MONARCH 1 and KEYNOTE-028, higher median PFS and OS were observed for cohort 2 [PFS: cohort 2: 8.9 months (95% CI: 3.9, 11.1); MONARCH 1: 6.0 months (95% CI: 4.2, 7.5); KEYNOTE-028: 1.8 months (95% CI: 1.4, 2.0 months); OS: cohort 2: 26.3 months (95% CI: 20.0, 31.0); MONARCH 1: 17.7 months (95% CI: 16.0-NR); KEYNOTE-028: 8.6 months (95% CI: 7.3, 11.6)]. While there are limitations to cross-trial comparisons, and numbers in this study are small, there is the possibility that abemaciclib plus pembrolizumab could have resulted in additive antitumor effects for heavily pretreated patients with HR+, HER2− MBC.

Some limitations should be considered when interpreting the data. Toxicity data and antitumor activity of cohort 1 and 2 were compared to abemaciclib with or without NSAIs or pembrolizumab monotherapy in similar patient populations. This study, however, was limited by the small sample size in both cohorts, and uncontrolled design means that comparisons directly against other available therapies are impossible. In addition, a high proportion of patients in cohort 2 were heavily and heterogeneously pretreated, further complicating comparisons.

While there is a suggestion that the combination of abemaciclib and pembrolizumab has antitumor activity in patients with HR+, HER2− MBC, there were higher rates of grade ≥3 transaminase elevations and ILD/pneumonitis, SAEs and treatment discontinuation due to AEs observed with abemaciclib plus pembrolizumab with/without anastrozole compared to previous reports. Overall, benefit/risk analysis based on the totality of data does not support further evaluation of this combination in the treatment of patients with HR+, HER2− MBC.

## Methods

### Study design and treatment

This was a multicenter, open-label, nonrandomized, multi-cohort Phase 1b study of abemaciclib plus pembrolizumab in patients with stage IV NSCLC, or HR+, HER2− MBC with or without anastrozole (NCT02779751; registration: May 2016). Patients were enrolled into four tumor-specific cohorts. Two cohorts included breast cancer patients, and this report contains the safety data from these cohorts. Cohort 1 comprised patients with HR+, HER2− MBC without any prior systemic anticancer therapy in the metastatic setting. Cohort 2 comprised patients with HR+, HER2− MBC previously treated with at least one but no more than two chemotherapy regimens in the metastatic setting. Results of the NSCLC cohorts were reported separately^26^. The study protocol was approved by institutional review boards and ethics committees (e.g., the University of California - UCSF institutional review board and ethics committee) before initiation and conducted in accordance with the Declaration of Helsinki. All patients provided written informed consent before participation in the trial.

Patients received abemaciclib (150 mg orally BID) on days 1 through 21 over 21-day cycles, in combination with pembrolizumab (200 mg infused iv over ~30 min) on day 1 of every cycle. Patients in cohort 1 also received anastrozole (1 mg orally) once daily.

Abemaciclib dose reduction was required for drug-related hematologic toxicity that was recurrent grade 3 or grade 4 or required administration of blood cell growth factors; persistent or recurrent grade 2 or grade 3–4 diarrhea or any grade diarrhea requiring hospitalization; persistent or recurrent grade 2 or grade 3 alanine aminotransferase (ALT)/ aspartate aminotransferase (AST) elevation without total bilirubin >2 × upper limit of normal (ULN), in the absence of cholestasis; grade 2 interstitial lung disease (ILD)/pneumonitis; or persistent or recurrent grade 2 or grade 3–4 nonhematologic toxicity not mentioned above. Abemaciclib was discontinued for ALT/AST elevation grade 3 with total bilirubin >2 × ULN or grade 4, or recurrent grade 2 or grade 3–4 ILD/pneumonitis.

Pembrolizumab was withheld (dose reductions were not permitted) for pre-specified drug-related toxicities and severe or life-threatening adverse events (AEs), including permanent discontinuation for the following: grade 4 diarrhea/colitis; grade 3–4 AST, ALT, or increased bilirubin; grade 4 hyperthyroidism; grade 3–4 infusion reaction; grade 3–4 or recurrent grade 2 pneumonitis; grade 3–4 renal failure/nephritis; grade 3–4 myocarditis; and grade 4 or recurrent grade 3 immune-related toxicities; and for any severe or grade 3 (grade 2 for pneumonitis) recurring drug-related AE or any life-threatening event.

Per the label, dose adjustments for anastrozole are not applicable, as only a single dose strength is approved for this medication.

In the event that one component of the combination had to be discontinued, patients could continue to receive other component(s) per the investigator’s clinical judgment. Treatment continued until disease progression, unacceptable toxicity, or any other discontinuation criteria was met. Study completion occurred following the final analysis of overall survival (OS), ~1 year after the last patient entered treatment. Patients who remained on study treatment at the time of study completion could continue receiving study treatment if they were experiencing clinical benefit and no undue risk.

### Study objectives

The primary objective was to characterize the safety profile of the combination of abemaciclib and pembrolizumab with or without anastrozole. The secondary objectives were to assess the preliminary efficacy in terms of ORR, PFS, duration of response (DoR), disease control rate (DCR), clinical benefit rate (CBR), and OS, and to characterize the PK profile of abemaciclib, pembrolizumab and anastrozole when given in combination.

### Patient population

Eligible patients for both cohort 1 and 2 had HR+, HER2− MBC, were male or female, and were at least 18 years old. Patients included in cohort 1 were postmenopausal and had not received prior systemic anticancer therapy in the metastatic setting. Patients could be enrolled if they had received ≤2 weeks of NSAI for the locally advanced or metastatic disease immediately preceding screening and agreed to discontinue NSAI until study treatment initiation. Patients included in cohort 2 were required to have received 1 or 2 chemotherapy regimens in the metastatic setting. Other key eligibility criteria for cohorts 1 and 2 included an Eastern Cooperative Oncology Group (ECOG) performance status of ≤1^27^, measurable disease as defined by Response Evaluation Criteria in Solid Tumors (RECIST 1.1)^27,28^, adequate organ function, and discontinuation of prior treatments before joining the study.

Key exclusion criteria common to both cohorts included: untreated central nervous system metastases; history of or active autoimmune disease; history of or current ILD/pneumonitis; active bacterial, fungal, and/or known viral infection (for example, human immunodeficiency virus [HIV] antibodies, hepatitis B surface antigen [HBSAg], or hepatitis C antibodies [HCAb]); prior exposure to anti-PD-1/PD-L1 agents or any CDK4 and 6 inhibitors.

### Safety and efficacy assessments

Safety evaluations included physical examination, clinical laboratory tests, and vital signs. Adverse event terms and severity grades were assessed using Common Terminology Criteria for Adverse Events (CTCAE) Version 4.0 and coded using the Medical Dictionary for Regulatory Activities (MedDRA) version 23.0.

Tumors were assessed by the investigator per RECIST 1.1 every 6 weeks for the first 48 weeks, and every 9 weeks thereafter. The baseline PD-L1 protein expression was assessed by an immunohistochemistry (IHC) assay in tumor tissue samples using DAKO PD-L1 22C3 IHC PharmDx (Catalog #SK006, Dako, Agilent, Carpinteria, CA, USA) performed at NeoGenomics Laboratories^29,30^. In both cohorts 1 and 2, approximately half of tissue biopsies came from the primary tumor and 50% from the metastatic sites. Baseline PD-L1 status was categorized as positive or negative, and a positive status was defined as PD-L1 expression score ≥1% per central testing.

### Endpoints

Safety endpoints included treatment-emergent adverse events (TEAEs) and serious adverse events (SAEs). TEAE was defined as an event that first occurred or worsened in severity after baseline. Efficacy endpoints included ORR, PFS, DoR, DCR according to RECIST 1.1., CBR and OS. ORR was defined as the proportion of patients achieving the best overall response (BOR) of confirmed partial response (PR) or complete response (CR). To be assigned a status of PR or CR, changes in tumor measurements had to have been confirmed by repeat assessments no less than 4 weeks after the criteria for response were first met. DoR was defined from the date of first documented CR or PR to the date of objective progression or the date of death due to any cause, whichever was earlier. DCR was defined as the proportion of patients achieving a BOR of CR, PR, or stable disease (SD) per RECIST v.1.1. CBR was defined as the proportion of evaluable patients achieving CR + PR + persistent SD (SD persisting ≥6 months). PFS was defined as the time from the date of first treatment until the date of radiographic progression (as defined by RECIST v.1.1) based on investigator assessment or the date of death due to any cause, whichever is earlier. OS was defined as the time from the date of first treatment to the date of death from any cause. For each patient who was not known to have died as of the data cutoff date, OS was censored at the date of the last contact date prior to the cutoff date.

### Pharmacokinetic

PK samples were collected prior to study drug administration on day 1 of cycles 1, 2, 3, 6, and 8, and also 8 h after study drug administration on day 1 of cycle 1. The concentrations of abemaciclib and its metabolites, M2 and M20, and anastrozole (cohort 1 only) were determined using validated Liquid Chromatography-Tandem Mass Spectrometric (LC-MS/MS) methods. Pembrolizumab concentrations were determined using a validated enzyme-linked immunosorbent assay (ELISA). PK analyses were conducted on all patients who received at least one dose of study treatment and had at least one evaluable PK sample.

### Statistical analyses

This was a nonrandomized, open-label, phase 1b study of abemaciclib plus pembrolizumab with safety as the primary endpoint. A sample size of 25 patients per cohort was planned to enroll in the study. As an example, for an observed adverse event rate of 12% in a cohort, a sample size of *N* = 25 would provide an 80% confidence interval (CI) of (4, 25.0%). Baseline characteristics, as well as safety data, were summarized by cohort. Both the safety and efficacy analyses were based on the safety population, which included all patients who received at least one dose of study treatment (abemaciclib and pembrolizumab with or without anastrozole). ORR and DCR were summarized and included exact 95% CI using Clopper-Pearson method. OS and PFS were analyzed using Kaplan-Meier (KM) method^31^; median and exact 95% CI were estimated. Individual changes in tumor burden over time are presented graphically (as waterfall plots). Safety data, such as TEAEs and deaths on study therapy, were summarized as the percentage of patients with one or more events.

### Reporting summary

Further information on research design is available in the Nature Research Reporting Summary linked to this article.

## Supplementary information

Reporting Summary

Supplementary Material

Protocol

## Data Availability

Eli Lilly and Company provides access to all individual participant data collected during the trial, after anonymization, with the exception of pharmacokinetic or genetic data. Data are available to request 6 months after the indication studied has been approved in the United States and the European Union and after primary publication acceptance, whichever is later. No expiration date of data requests is currently set once data are made available. Access is provided after a proposal has been approved by an independent review committee identified for this purpose and after receipt of a signed data sharing agreement. Data and documents (including the study protocol, statistical analysis plan, clinical study report, and blank or annotated case report forms) will be provided in a secure data sharing environment. For details on submitting a request, see the instructions provided at www.vivli.org.
