# Supplementary material for: Abemaciclib in combination with pembrolizumab for HR+, HER2− metastatic breast cancer: Phase 1b study
Source: NPJ Breast Cancer. 2022 Nov 5;8:118. doi: 10.1038/s41523-022-00482-2 (PMC9637121; doi:10.1038/s41523-022-00482-2)
Supplement: Supplementary file 1 — Reporting Summary [file 41523_2022_482_MOESM1_ESM.pdf]

nature portfolio

Corresponding author(s): Dr. Hope Rugo

Last updated by author(s): 20/06/2022

Reporting Summary

Nature Portfolio wishes to improve the reproducibility of the work that we publish. This form provides structure for consistency and transparency in reporting. For further information on Nature Portfolio policies, see our Editorial Policies and the Editorial Policy Checklist.

Statistics

For all statistical analyses, confirm that the following items are present in the figure legend, table legend, main text, or Methods section.

n/a	Confirmed	
☐	☒	The exact sample size (n) for each experimental group/condition, given as a discrete number and unit of measurement
☐	☒	A statement on whether measurements were taken from distinct samples or whether the same sample was measured repeatedly
☐	☒	The statistical test(s) used AND whether they are one- or two-sided<br>*Only common tests should be described solely by name; describe more complex techniques in the Methods section.*
☐	☒	A description of all covariates tested
☐	☒	A description of any assumptions or corrections, such as tests of normality and adjustment for multiple comparisons
☐	☒	A full description of the statistical parameters including central tendency (e.g. means) or other basic estimates (e.g. regression coefficient) AND variation (e.g. standard deviation) or associated estimates of uncertainty (e.g. confidence intervals)
☐	☒	For null hypothesis testing, the test statistic (e.g. F, t, r) with confidence intervals, effect sizes, degrees of freedom and P value noted<br>*Give P values as exact values whenever suitable.*
☒	☐	For Bayesian analysis, information on the choice of priors and Markov chain Monte Carlo settings
☒	☐	For hierarchical and complex designs, identification of the appropriate level for tests and full reporting of outcomes
☒	☐	Estimates of effect sizes (e.g. Cohen's d, Pearson's r), indicating how they were calculated

Our web collection on statistics for biologists contains articles on many of the points above.

Software and code

Policy information about availability of computer code

Data collection	The data collection was saved in SAS data format.
Data analysis	Statistical analyses were performed in SAS 9.2 or higher.

For manuscripts utilizing custom algorithms or software that are central to the research but not yet described in published literature, software must be made available to editors and reviewers. We strongly encourage code deposition in a community repository (e.g. GitHub). See the Nature Portfolio guidelines for submitting code & software for further information.

Data

Policy information about availability of data

All manuscripts must include a data availability statement. This statement should provide the following information, where applicable:

- Accession codes, unique identifiers, or web links for publicly available datasets
- A description of any restrictions on data availability
- For clinical datasets or third party data, please ensure that the statement adheres to our policy

Data Availability Statement:

Eli Lilly and Company provides access to all individual participant data collected during the trial, after anonymization, with the exception of pharmacokinetic or genetic data. Data are available to request 6 months after the indication studied has been approved in the United States and the European Union and after primary publication acceptance, whichever is later. No expiration date of data requests is currently set once data are made available. Access is provided after a proposal has been approved by an independent review committee identified for this purpose and after receipt of a signed data sharing agreement. Data and documents

(including the study protocol, statistical analysis plan, clinical study report, and blank or annotated case report forms) will be provided in a secure data sharing environment. For details on submitting a request, see the instructions provided at www.vivli.org.

Field-specific reporting

Please select the one below that is the best fit for your research. If you are not sure, read the appropriate sections before making your selection.

☒ Life sciences ☐ Behavioural & social sciences ☐ Ecological, evolutionary & environmental sciences

For a reference copy of the document with all sections, see nature.com/documents/nr-reporting-summary-flat.pdf

Life sciences study design

All studies must disclose on these points even when the disclosure is negative.

Sample size	A sample size of 25 patients per cohort was planned to enroll in the study. As an example, for an observed adverse event rate of 12% in a cohort, a sample size of N=25 would provide an 80% confidence interval (CI) of (4, 25.0%).
Data exclusions	No data were excluded from the analyses. Both the safety and efficacy analyses were based on the safety population which included all patients who received at least one dose of study treatment (abemaciclib and pembrolizumab with or without anastrozole).
Replication	Independent replication was done during the programming.
Randomization	This is a multicenter, open-label, nonrandomized, multi-cohort Phase 1b study.
Blinding	This study is open-label.

Reporting for specific materials, systems and methods

We require information from authors about some types of materials, experimental systems and methods used in many studies. Here, indicate whether each material, system or method listed is relevant to your study. If you are not sure if a list item applies to your research, read the appropriate section before selecting a response.

Materials & experimental systems

n/a	Involved in the study	
☒	☐	Antibodies
☒	☐	Eukaryotic cell lines
☒	☐	Palaeontology and archaeology
☐	☐	Animals and other organisms
☐	☒	Human research participants
☐	☒	Clinical data
☒	☐	Dual use research of concern

Methods

n/a	Involved in the study	
☒	☐	ChIP-seq
☒	☐	Flow cytometry
☒	☐	MRI-based neuroimaging

Animals and other organisms

Policy information about studies involving animals; ARRIVE guidelines recommended for reporting animal research

Laboratory animals	NA - This is a clinical trial with human participants.
Wild animals	NA - This is a clinical trial with human participants.
Field-collected samples	NA - This is a clinical trial with human participants.
Ethics oversight	NA - This is a clinical trial with human participants.

Note that full information on the approval of the study protocol must also be provided in the manuscript.

Human research participants

Policy information about studies involving human research participants

Population characteristics	Cohort 1 comprised of patients with metastatic HR+, HER2- breast cancer without any prior systemic anticancer therapy in the metastatic setting. Cohort 2 comprised of patients with HR+, HER2- MBC previously treated with at least one but no more than two chemotherapy regimens in the metastatic setting. Baseline patient demographics and disease characteristics for

each cohort in this clinical trial are outlined in Table 1.

Recruitment	Patients were recruited globally "Twenty-six patients from two different countries (n=10 Belgium; n=16 United States) were enrolled in cohort 1 and received treatment with abemaciclib in combination with pembrolizumab and anastrozole. Twenty-eight patients from six different countries/regions (n=1 Taiwan; n=2 Belgium; n=6 Spain; n=1 France; n=2 Italy; n=16 USA) were enrolled in cohort 2 and received treatment with abemaciclib in combination with pembrolizumab."
Ethics oversight	We have provided the following ethical statement in the manuscript:<br>"The study protocol was approved by institutional review boards and ethics committees before initiation and conducted in accordance with the Declaration of Helsinki. All patients provided written informed consent before participation in the trial."

Note that full information on the approval of the study protocol must also be provided in the manuscript.

Clinical data

Policy information about clinical studies

All manuscripts should comply with the ICMJE guidelines for publication of clinical research and a completed CONSORT checklist must be included with all submissions.

Clinical trial registration	Clinical Trial Number: NCT02779751
Study protocol	Not openly available on-line; a redacted version of the protocol is provided.
Data collection	Between November 2016 and August 2019, fifty-four patients were enrolled into cohort 1 and 2 of this study.
Outcomes	How primary and secondary outcomes are predefined and how they are assessed is outlined in the methods section of this manuscript.
