# Supplementary material for: Abemaciclib in combination with pembrolizumab for HR+, HER2− metastatic breast cancer: Phase 1b study
Source: NPJ Breast Cancer. 2022 Nov 5;8:118. doi: 10.1038/s41523-022-00482-2 (PMC9637121; doi:10.1038/s41523-022-00482-2)

Supplemental Data

Supplementary Figure 1. PK of (A) abemaciclib (150 mg PO Q12H), (B) pembrolizumab (200mg iv Q21D), and (C) anastrozole (1 mg PO Q24H)

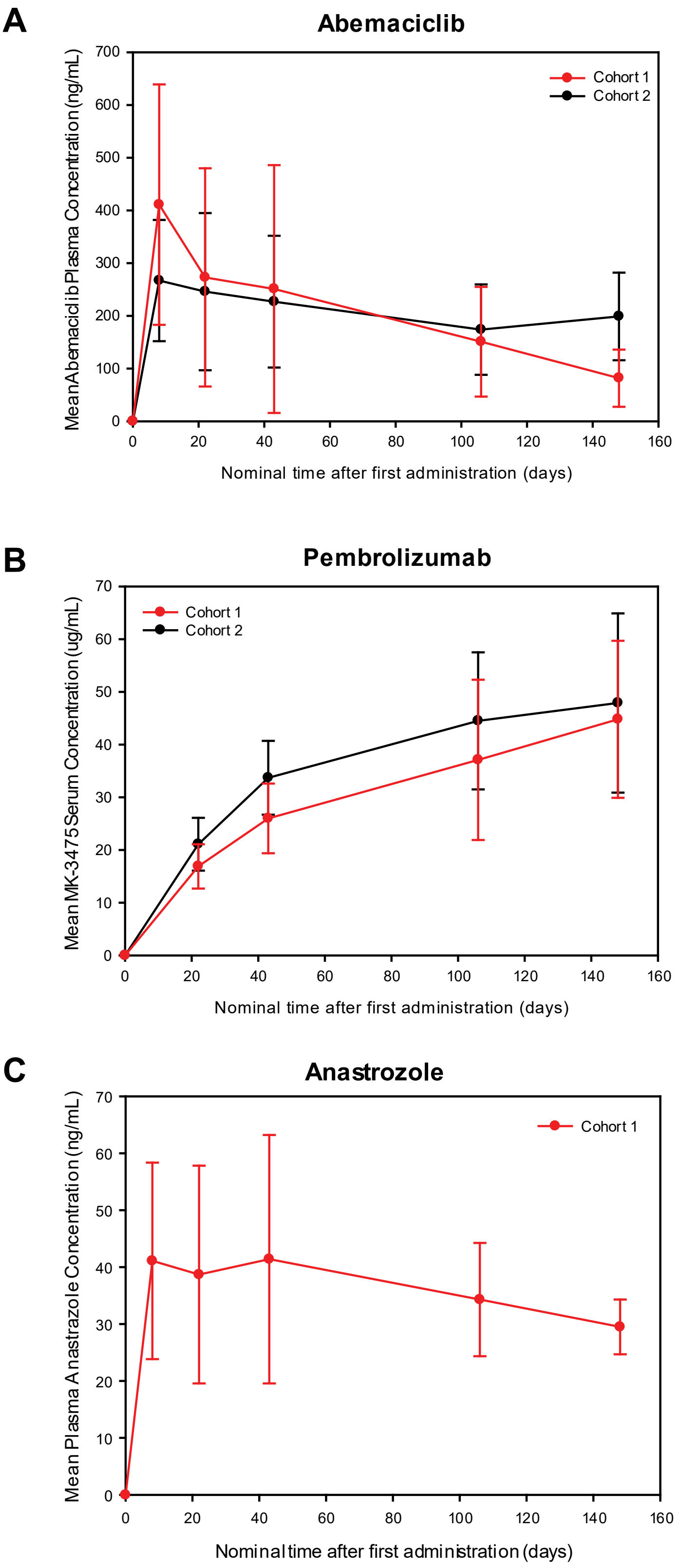

Figure Legend: Arithmetic mean plasma concentration over time for (A) abemaciclib (ng/mL), (B) pembrolizumab (µg/mL) and (C) anastrozole (ng/mL). Error bars indicate standard deviation.

Abbreviation: IV = intravenous administration; PO = oral administration; Q21H = every 12 hours; Q21D = every 21 days; Q24H = every 24 hours

Supplementary Figure 2. Best percent change in tumor size from baseline (RECIST version 1.1) according to PD-L1 status in Cohort 2[a]

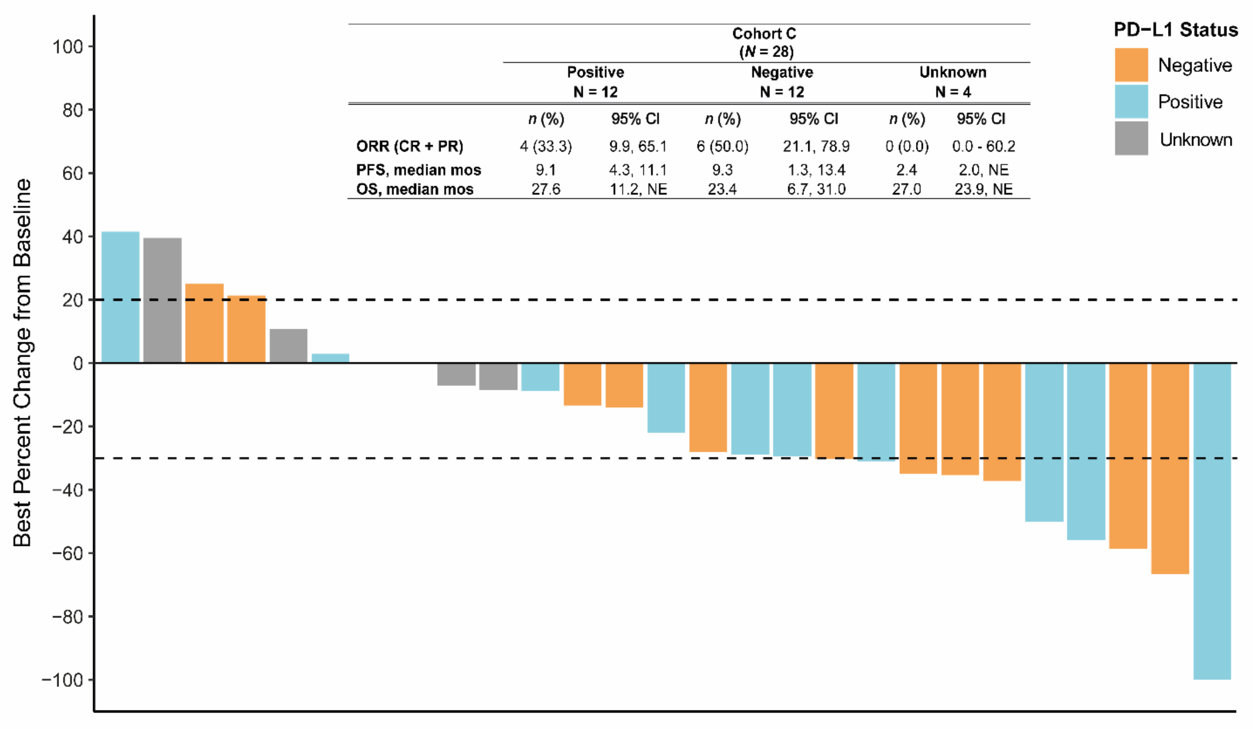

Figure legend: Best percent change in tumor size from baseline is presented for the safety population in cohort 1. The PD-L1 protein expression was assessed by an IHC assay in tumor tissue samples (see Methods).

[a]Due to sample size limitation (Table 1), the anticancer activity by PD-L1 status was not presented for cohort 1

Note: Patients without any post baseline data are not included in the graphs.

Abbreviation: CR, complete response; CI, confidence interval; NE, non-estimable; ORR, overall response rate; OS, overall survival; PFS, progression free survival; PD-L1, Programmed death-ligand 1; PR, Partial Response.

Supplementary Figure 3. (A) Overall survival (OS) and (B) progression-free survival (PFS) in Cohorts 1 and 2

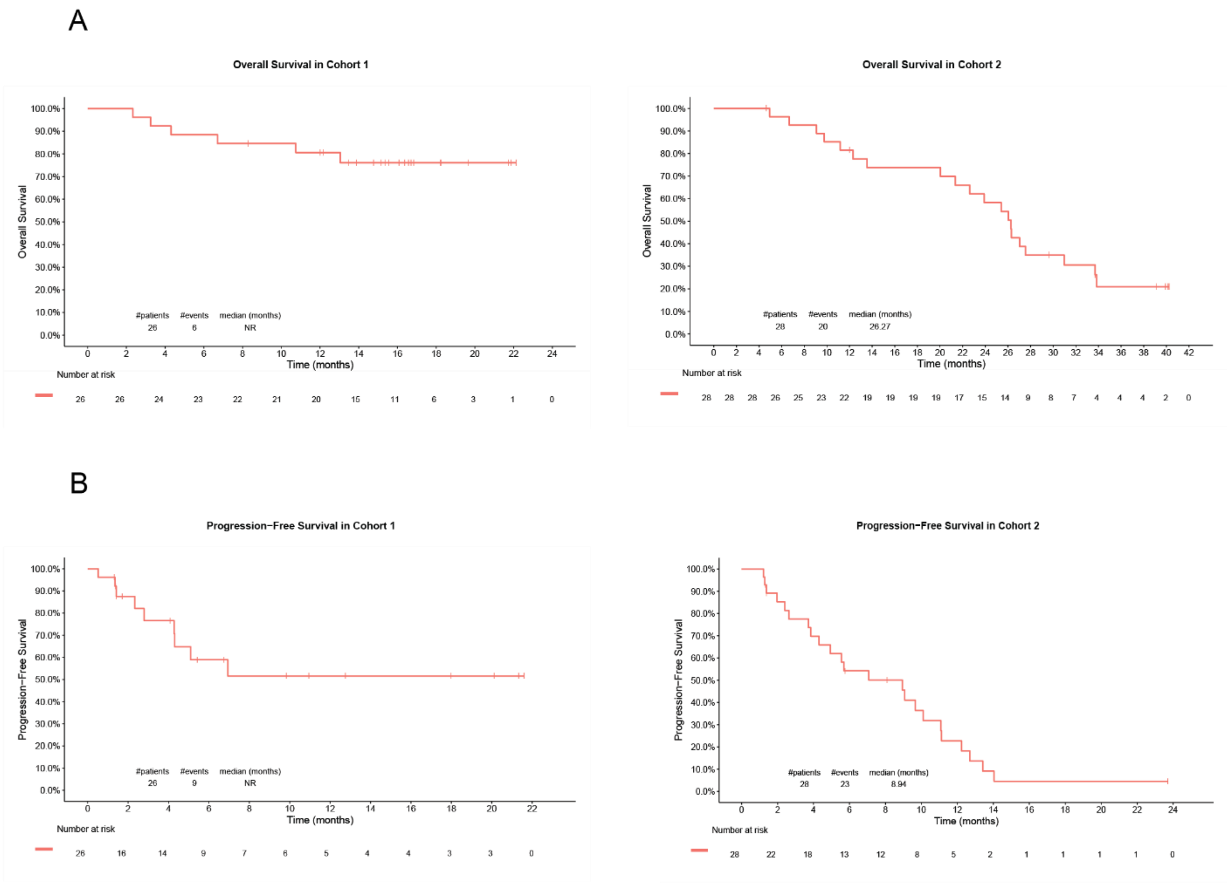

Supplement: Supplementary file 2 — Supplementary Material [file 41523_2022_482_MOESM2_ESM.pdf]
