# Supplementary material for: Abemaciclib in combination with pembrolizumab for HR+, HER2− metastatic breast cancer: Phase 1b study
Source: NPJ Breast Cancer. 2022 Nov 5;8:118. doi: 10.1038/s41523-022-00482-2 (PMC9637121; doi:10.1038/s41523-022-00482-2)
Supplement: Supplementary file 3 — Protocol [file 41523_2022_482_MOESM3_ESM.pdf]

Protocol I3Y-MC-JPCE(e)
A Phase 1b Study of Abemaciclib in Combination with Pembrolizumab for Patients with Stage IV Non-Small Cell Lung Cancer or Hormone Receptor-Positive, HER2-Negative Breast Cancer

Abemaciclib (LY2835219)

Eli Lilly and Company
Indianapolis, Indiana USA 46285

Protocol Electronically Signed and Approved by Lilly: 22 January 2016
Amendment (a) Approved by Lilly: 20 December 2016
Amendment (b) Approved by Lilly: 18 July 2018
Amendment (c) Approved by Lilly: 08 February 2019
Amendment (d) Approved by Lilly: 25 February 2020
Amendment (e) Electronically Signed and Approved by Lilly
on approval date provided below.

Approval Date: 10-Feb-2021 GMT

Table of Contents

Section **Page**

1. Synopsis 8
2. Schedule of Activities 10
3. Introduction 17
3.1. Study Rationale 17
3.1.1. Rationale for Amendment (a) 17
3.1.2. Rationale for Amendment (b) 17
3.1.3. Rationale for Amendment (c) 18
3.1.4. Rationale for Amendment (d) 18
3.1.5. Rationale for Amendment (e) 19
3.2. Background 19
3.3. Benefit/Risk Assessment 21
4. Objectives and Endpoints 22
5. Study Design 23
5.1. Overall Design 23
5.2. Number of Patients 23
5.3. End of Study Definition 23
5.4. Scientific Rationale for Study Design 23
5.5. Justification for Dose 24
6. Study Population 26
6.1. Inclusion Criteria 26
6.2. Exclusion Criteria 28
6.3. Lifestyle Restrictions 30
6.4. Screen Failures 30
7. Treatments 31
7.1. Treatments Administered 31
7.1.1. Packaging and Labelling 32
7.2. Method of Treatment Assignment 32
7.2.1. Selection and Timing of Doses 32
7.3. Blinding 32
7.4. Dosage Modification 32
7.4.1. Special Treatment Considerations 32
7.4.1.1. Dose Adjustments and Delays 32
7.4.1.1.1. Dose Adjustments 34
7.4.1.1.1.1. Abemaciclib 34
7.4.1.1.1.2. Pembrolizumab 37

7.4.1.1.2. Dose Delays and Omission 38
7.4.1.1.2.1. Abemaciclib 38
7.4.1.1.2.2. Pembrolizumab 39
7.5. Preparation/Handling/Storage/Accountability....40
7.6. Treatment Compliance 41
7.7. Concomitant Therapy 41
7.7.1. Supportive Care 42
7.7.1.1. Abemaciclib 43
7.7.1.1.1. Supportive Management of Diarrhea 43
7.7.1.2. Pembrolizumab....44
7.8. Treatment after the End of the Study 48
7.8.1. Continued Access....48
8. Discontinuation Criteria 50
8.1. Discontinuation from Study Treatment 50
8.1.1. Discontinuation of Inadvertently Enrolled Patients 50
8.2. Discontinuation from the Study 51
8.3. Lost to Follow-Up 51
9. Study Assessments and Procedures 52
9.1. Efficacy Assessments 52
9.1.1. Appropriateness of Assessments 52
9.2. Adverse Events 52
9.2.1. Serious Adverse Events....53
9.2.1.1. Events of Clinical Interest - Pembrolizumab 54
9.2.2. Suspected Unexpected Serious Adverse Reactions 55
9.2.3. Complaint Handling 55
9.3. Treatment of Overdose....55
9.4. Safety....55
9.4.1. Other Safety Measures 55
9.4.2. Safety Monitoring 56
9.4.2.1. Special Hepatic Safety Data Collection....56
9.4.2.2. Renal Function 57
9.4.2.3. Venous Thromboembolic Events 57
9.4.2.4. Interstitial Lung Disease/Pneumonitis....57
9.5. Pharmacokinetics 58
9.6. Pharmacodynamics 58
9.7. Pharmacogenomics 59
9.7.1. Samples for Pharmacogenetic Research 59
9.8. Biomarkers....59

9.8.1. Samples for Nonpharmacogenetic Biomarker Research....60
9.8.2. Tissue Samples for Disease Confirmation and Research....60
9.9. Health Economics....61
10. Statistical Considerations....63
10.1. Sample Size Determination....63
10.2. Populations for Analyses....63
10.3. Statistical Analyses....64
10.3.1. General Consideration....64
10.3.2. Efficacy Analyses....64
10.3.3. Primary Outcome and Methodology (Safety and Tolerability)....65
10.3.4. Other Analyses....66
10.3.4.1. Disposition....66
10.3.4.2. Patient Characteristics....66
10.3.4.3. Concomitant Therapy....66
10.3.4.4. Treatment Compliance....67
10.3.4.5. Pharmacokinetics....67
10.3.4.6. Biomarkers....67
10.3.4.7. Health Outcomes....67
10.3.4.7.1. Health Outcomes and Economics....68
10.3.5. Subgroup Analyses....68
10.3.6. Interim Analyses....68
11. References....69

List of Tables

Table **Page**

Table JPCE.2.1. Baseline Assessments Schedule of Activities 10

Table JPCE.2.2. On-Study-Treatment Schedule of Activities 12

Table JPCE.2.3. Post-Treatment Follow-Up Schedule of Activities 14

Table JPCE.2.4. Continued Access Schedule of Activities 16

Table JPCE.4.1. Objectives and Endpoints 22

Table JPCE.7.1. Treatment Regimens 31

Table JPCE.7.2. Toxicity Dose Adjustments and Delays of Abemaciclib 33

Table JPCE.7.3. Dose Adjustments of Abemaciclib 35

Table JPCE.7.4. General Dose Delays of Abemaciclib 38

Table JPCE.7.5. Dose Modification Guidelines for Pembrolizumab 39

Table JPCE.7.6. Infusion Reaction Treatment Guidelines 47

Table JPCE.10.1. Estimated Incidence Rate and Its 2-Sided 80% CI 63

Table JPCE.10.2. PFS Event/Censoring Scheme by RECIST 1.1 65

List of Figures

Figure **Page**

Figure JPCE.5.1. Illustration of study design for I3Y-MC-JPCE. 23
Figure JPCE.7.1. Continued access diagram..48

List of Appendices

Appendix		Page
Appendix 1.	Abbreviations and Definitions	71
Appendix 2.	Study Governance, Regulatory, and Ethical Considerations	74
Appendix 3.	Clinical Laboratory Tests	77
Appendix 4.	Sampling Schedule for Biomarkers/ Pharmacokinetics/ Pharmacodynamics	78
Appendix 5.	Creatinine Clearance Formula	80
Appendix 6.	Protocol JPCE Inducers and Strong Inhibitors of CYP3A	81
Appendix 7.	RECIST 1.1 and irRECIST Criteria	82
Appendix 8.	ECOG Performance Status	91
Appendix 9.	CTCAE 4.03 Definitions for Diarrhea and ILD/Pneumonitis	92
Appendix 10.	Protocol JPCE Hepatic Monitoring Tests for Treatment-Emergent Abnormality	94
Appendix 11.	Protocol Amendment I3Y-MC-JPCE(e) Summary: A Phase 1b Study of Abemaciclib in Combination with Pembrolizumab for Patients with Stage IV Non-Small Cell Lung Cancer or Hormone Receptor-Positive, HER2-Negative Breast Cancer	95

1. Synopsis

Protocol Title:

A Phase 1b Study of Abemaciclib in Combination with Pembrolizumab for Patients with Stage IV Non-Small Cell Lung Cancer or Hormone Receptor-Positive, HER2-Negative Breast Cancer

Summary:

Study I3Y-MC-JPCE (JPCE) is a Phase 1b trial for patients with Stage IV non-small cell lung cancer (NSCLC) or hormone receptor positive (HR+), human epidermal growth factor receptor 2 negative (HER2-) breast cancer that will further evaluate the safety of abemaciclib in combination with pembrolizumab in approximately 75 patients. This study will also evaluate preliminary efficacy in 3 patient populations.

Objectives and Endpoints:

Objectives	Endpoints
**Primary**	
To characterize further the safety profile of the combination of abemaciclib and pembrolizumab	Safety endpoints per CTCAE v 4.0, NCI 2009 and will include but are not limited to the following:<br>• TEAEs, ECIs, SAEs, and hospitalizations<br>• Clinical laboratory tests, vital signs, and physical examinations
**Secondary**	
To assess the preliminary efficacy of abemaciclib in combination with pembrolizumab	Efficacy endpoints will include;<br>• ORR per tumor assessment using RECIST v1.1<br>• PFS per RECIST v1.1<br>• DoR per RECIST v1.1<br>• DCR per RECIST v1.1<br>• OS
To characterize the PK of abemaciclib and pembrolizumab when given in combination	Abemaciclib and pembrolizumab concentrations in plasma or serum

Abbreviations: CTCAE = Common Terminology Criteria for Adverse Events; DCR = disease control rate; DoR = duration of response; ECI = Events of Clinical Interest; NCI = National Cancer Institute; ORR = objective response rate; OS = overall survival; PFS = progression-free survival; PK = pharmacokinetics; RECIST = Response Evaluation Criteria in Solid Tumors; SAE = serious adverse events; TEAE = treatment-emergent adverse event.

Overall Design:

Study JPCE is a multicenter, non-randomized, open-label, Phase 1b study of abemaciclib in combination with pembrolizumab in patients with Stage IV NSCLC or HR+, HER2- breast cancer.

Number of Patients:

Approximately 75 patients with Stage IV NSCLC or with HR+, HER2- breast cancer will be enrolled in the study.

Treatment Arms and Duration:

The tumor-specific cohorts will consist of 25 patients with Kirsten rat sarcoma mutant (KRAS mt) NSCLC expressing ≥ 1% programmed death ligand 1 (PD-L1), 25 patients with NSCLC with squamous histology, and 25 patients with HR+, HER2- metastatic breast cancer.

The planned duration is not fixed (that is, patient will remain on study treatment until disease progression by immune-related Response Evaluation Criteria in Solid Tumors [irRECIST], unacceptable toxicity, patient fulfills one of the criteria for study discontinuation [Section 8], or 24 months of pembrolizumab therapy [whichever occurs first]). Abemaciclib will continue to progression regardless of duration of therapy.

Dose and Schedule (q 21 Days)			
**Arm**	**Patients/Cohort**	**Abemaciclib Dose**	**Pembrolizumab Dose**
A	Stage IV NSCLC *KRAS mt,* PD-L1+	150 mg Q12H	200 mg Day 1 of a 21-day cycle
B	Stage IV NSCLC squamous	150 mg Q12H	200 mg Day 1 of a 21-day cycle
C	HR+, HER2- metastatic breast cancer	150 mg Q12H	200 mg Day 1 of a 21-day cycle

Abbreviations: HER2- = human epidermal growth factor receptor 2 negative; HR+ = hormone receptor positive; *KRAS mt* = Kirsten rat sarcoma mutant; NSCLC – non-small cell lung cancer; PD-L1 = programmed death ligand 1; q= every; Q12H = every 12 hours.

2. Schedule of Activities

Table JPCE.2.1. Baseline Assessments Schedule of Activities

Day relative to C1D1	≤28	≤14	≤7	Instructions
**Procedure**				
Informed consent	X			ICF must be signed before any protocol-specific procedures are performed
Inclusion/exclusion criteria		X		Collection of tumor tissue is required for ALL patients for eligibility. Sites should confirm the availability of adequate tumor tissue with the pathological laboratory prior to randomization. Arm A: *KRAS mt* testing will be done via local labs. PD-L1 testing will be done via central lab. Arm C: Most recent HR and HER2 receptor testing should be used to determine eligibility.
Physical examination		X		Including height, weight, and vital signs (for example: temperature, blood pressure, pulse rate, respiration rate)
ECOG performance status		X		
Medical history		X		Including assessment of preexisting conditions, historical illnesses, and habits (such as tobacco /alcohol)
Prior and current medication		X		
AE collection		X		CTCAE Version 4.0; Obtain only after study eligibility is confirmed.
ECG	X			Local ECG performed at baseline. Patient eligibility may be determined with a QTc interval performed within 90 days prior to study enrollment.
Radiological tumor assessment	X			RECIST 1.1/irRECIST; Scans (for example: MRI/CT) will be performed and reviewed locally.
Tumor measurement (palpable or visible)		X		
Brain MRI/CT		X		Only required for patients with treated brain metastases or for patients with evidence of neurological changes 14 days prior to receiving study drug to rule out CNS metastases.
Hematology		X		See Appendix 3
Coagulation		X		See Appendix 3
Clinical chemistry		X		See Appendix 3
Thyroid Function		X		See Appendix 3
Serum pregnancy test			X	Applies only to women of childbearing potential. Must have a negative urine or serum pregnancy test within 72 hours before the first dose of study drug (i.e., Day -3 to Day -1).

Baseline Assessments Schedule of Activities

Day relative to C1D1	≤28	≤14	≤7	Instructions
MDASI* questionnaire		X		Patients should complete before interaction with site staff.
Sample collection	For all sample collection, see Appendix 4			
Pharmacodynamics				
Pharmacokinetics				
Genetics				
Biomarkers				
Tumor biopsy				

Abbreviations: AE = adverse event; C1D1 = Cycle 1 Day 1; CNS = central nervous system; CT = computer tomography; CTCAE = Common Terminology Criteria for Adverse Events (NCI 2009); ECG = electrocardiogram; ECOG = Eastern Cooperative Oncology Group; HER2 = human epidermal growth factor receptor 2; HR = hormone receptor; ICF = informed consent form; irRECIST = immune-related Response Evaluation Criteria in Solid Tumors; *KRAS mt* = Kirsten rat sarcoma mutant; MDASI = MD Anderson Symptom Inventory questionnaire, *= inclusion of a supplemental sheet consisting of 2 additional items (worst cough and worst diarrhea); MRI = magnetic resonance imaging; PD-L1 = programmed death ligand 1; RECIST 1.1 = Response Criteria in Solid Tumors Version 1.1.

Table JPCE.2.2. On-Study-Treatment Schedule of Activities

Relative Day within cycle	Cycle 1		Cycle 2 and Beyond (per instructions)		
	1(-3)	8	1(-3)	8	
**Procedure**					**Instructions**
Physical examination	X		X		Including weight and vital signs (example: temperature, blood pressure, pulse rate, respiration rate)
Prior and current medication	X		X		
AE collection	X		X		CTCAE Version 4.0.
ECOG performance status	X		X		
Radiological tumor assessment (example: MRI/CT)			X		RECIST 1.1/irRECIST; Scans will be performed and reviewed locally; Done every 6 weeks ± 7 days from Cycle 1, Day 1 for 48 weeks, then every 9 weeks thereafter. The same method of assessment and technique used at baseline should be used for each consecutive assessment thereafter.
Tumor measurement (palpable or visible)			X		
Hematology	X	X	X	X	See Appendix 3. Day 8 performed for Cycle 1-8 only.
Coagulation	Only when clinically indicated				See Appendix 3. Should be obtained throughout the study when clinically indicated.
Clinical chemistry	X	X	X	X	See Appendix 3. Day 8 performed for Cycle 1-8 only.
Thyroid Function			X		See Appendix 3. Starting Cycle 2, Day 1 and every other cycle thereafter.
MDASI* questionnaire	X		X		Patients should complete before interaction with site staff. Completed Cycle 1, Day 1; Cycle 2, Day 1; every cycle thereafter; and at disease progression (if applicable).
Study Drug					
Abemaciclib	Daily every 12 hours				Abemaciclib is to be administered every 12 hours on Days 1 through 21 of each cycle.

On-Study-Treatment Schedule of Activities

Relative Day within cycle	Cycle 1		Cycle 2 and Beyond (per instructions)		
	1(-3)	8	1(-3)	8	
**Procedure**					**Instructions**
Pembrolizumab	X		X		Pembrolizumab is to be administered on Day 1 of every cycle (each cycle is 21 days).
Sample collection	For all sample collection, see Appendix 4				In the event of an infusion-related reaction, blood samples for PK analysis will be collected as close to the onset of the reaction as possible, at the resolution of the event, and 30 days following the event.
Pharmacodynamics					
Pharmacokinetics					
Genetics					
Biomarkers					
Tumor Biopsy					

Abbreviations: AE = adverse event; CT = computer tomography; CTCAE = Common Terminology Criteria for Adverse Events (NCI 2009); ECG = electrocardiogram; ECOG = Eastern Cooperative Oncology Group; irRECIST = Immune-related Response Evaluation Criteria in Solid Tumors; MDASI = MD Anderson Symptom Inventory questionnaire, *= inclusion of supplemental sheet consisting of 2 additional items (worst cough and worst diarrhea); MRI = magnetic resonance imaging; PK = pharmacokinetics; RECIST 1.1 = Response Criteria in Solid Tumors Version 1.1.

Table JPCE.2.3. Post-Treatment Follow-Up Schedule of Activities

Visit	Short-Term Follow-Up[a]	Long-Term Follow-Up[b]	
	801	802-8XX	
**Procedure**			**Instructions**
Physical examination	X		Including weight and vital signs (example: temperature, blood pressure, pulse rate, respiration rate)
Prior and current medication	X		
AE collection	X	X	CTCAE Version 4.0. During long-term follow-up, only SAEs that are related to study regimen or protocol procedure will be collected.<br>All drug- or procedure-related AEs and SAEs should be followed until they resolve, are no longer considered to be drug- or procedure-related, become stable or return to baseline, the patient starts a new therapy, the patient dies, or the patient becomes lost to follow-up. Frequency of evaluation is left to the judgment of the investigator.
ECOG performance status	X		
Radiological tumor assessment (example: MRI/CT)	X	X	RECIST 1.1/irRECIST; Scans will be performed and reviewed locally. If a patient is discontinued from the study, repeat radiology may be omitted if progressive disease can be documented quantitatively with clinical measurements.<br>Patients who discontinue study treatment without objectively measured progressive disease continue to evaluate tumor response according to planned tumor assessment schedule until patient has objective disease progression or study completion.
Tumor measurement (palpable or visible)	X		

Post-Treatment Follow-Up Schedule of Activities

Visit	Short-Term Follow-Up[a] 801	Long-Term Follow-Up[b] 802-8XX	
**Procedure**			**Instructions**
Collection of survival information		X	Although preferable to collect during a clinic visit, survival information may be collected by contacting the patient or family directly (for example, via telephone) if no procedures are required. This should be collected approximately every 90 days.
Collection of post-study-treatment anticancer therapy information	X	X	Perform every 90 days for the first 2 years after discontinuation from study treatment and every 6 months (±14 days)] thereafter until death or study completion.
Hematology	X		See Appendix 3
Coagulation	X		PT or INR and aPTT should be collected at the mandatory Safety Follow-Up Visit after discontinuation of study therapy.
Clinical chemistry	X		See Appendix 3
Thyroid Function	X		See Appendix 3
MDASI* questionnaire	X		Patients should complete before interaction with site staff
Sample collection	For all sample collection, see Appendix 4		
Biomarkers			

Abbreviations: AE = adverse event; aPTT = activated partial thromboplastin time; CT = computer tomography; CTCAE = Common Terminology Criteria for Adverse Events (NCI 2009); ECOG = Eastern Cooperative Oncology Group; INR = international normalized ratio; irRECIST = immune-related Response Evaluation Criteria in Solid Tumors; MDASI = MD Anderson Symptom Inventory questionnaire, *= inclusion of a supplemental sheet consisting of 2 additional items (worst cough and worst diarrhea); MRI = magnetic resonance imaging; PT = prothrombin time; RECIST 1.1 = Response Criteria in Solid Tumors Version 1.1; SAE = serious adverse event.

[a] Short-term follow up starts the day after the patient and the investigator agree that the patient will no longer continue study treatment and ends when final safety assessments are completed 30 days (±7) after last dose of study drug.

[b] Long-term follow up begins the day after short-term follow up is completed and continues every 90 days until the patient's death or overall study completion.

Table JPCE.2.4. Continued Access Schedule of Activities

Visit	Study Treatment 501-5XX	Follow-Up[a] 901	Instructions
**Procedure[b]**			
AE collection	X	X	CTCAE Version 4.0
Administer study drug	X		

Abbreviations: AE = adverse event; CTCAE = Common Terminology Criteria for Adverse Events.

a Continued access follow-up begins the day after the patient and the investigator agree that the patient will no longer continue treatment in the continued access period and lasts approximately 30 days. No follow-up procedures will be performed for a patient who withdraws informed consent unless he or she has explicitly provided permission and consent.

b Efficacy assessments will be done at the investigator's discretion based on the standard of care.

3. Introduction

3.1. Study Rationale

Study I3Y-MC-JPCE (JPCE) is a Phase 1b trial for patients with Stage IV non-small cell lung cancer (NSCLC) or hormone receptor positive (HR+), human epidermal growth factor receptor 2 negative (HER2-) breast cancer that will further evaluate the safety of abemaciclib in combination with pembrolizumab in approximately 75 patients. This study will also evaluate preliminary efficacy in the 3 patient populations.

3.1.1. Rationale for Amendment (a)

This protocol amendment provides an extended dose justification for the combination of abemaciclib and pembrolizumab including the preliminary safety results of Study JPBJ, Part E.

Additional hematology and chemistry monitoring have been incorporated on Day 8 during the first 8 cycles in all study arms based on results from neoMONARCH (I3Y-MC-JPBY). In the neoMONARCH trial resected breast cancer specimens after 14 weeks of abemaciclib monotherapy revealed increased infiltration with CD3 and CD8 lymphocytes compared to pre-treatment specimens. This observation suggests the theoretical possibility that abemaciclib could have an additive or synergistic effect with pembrolizumab in terms of immune-related side effects such as hepatitis. More frequent monitoring allows for earlier detection should the side effects occur and earlier intervention.

ECHO/MUGA and ECG procedures were omitted as there was no medical reason to include these tests.

Furthermore, pembrolizumab background information has been updated to align with the approved label and the thyroid function inclusion criteria has been removed.

Minor editorial changes have been made throughout the protocol to improve clarity and practicability of the protocol and secure alignment with the intended study design.

3.1.2. Rationale for Amendment (b)

During the course of Study JPCE, the formulation for pembrolizumab will change from a lyophilized powder to a liquid. Sites will be allowed to use either formulation, as both are approved for use in the United States and the European Union. The amendment outlines the needed changes that will accompany the liquid formulation (Section 7.5).

Response Criteria for Solid Tumors (RECIST) v1.1 is still the scientifically accepted standard for tumor assessment. Tumor assessment based on immune-related Response Evaluation Criteria in Solid Tumors (irRECIST) is considered to be exploratory (Seymour et al. 2017). In this amendment, the objective response rate (ORR) and progression-free survival (PFS) objectives based on irRECIST have been moved to exploratory objectives. In addition, patient-reported pain and disease-related symptoms outcome based on MD Anderson Symptom Inventory questionnaire (MDASI) has also been moved to exploratory objectives, as this is considered exploratory analysis in this single-arm trial.

Minor editorial changes have been made throughout the protocol to improve clarity and practicability of the protocol and to secure alignment with the intended study design.

3.1.3. Rationale for Amendment (c)

Study JPCE protocol was amended to update the dosing guidance for cases of nonhematologic toxicity, diarrhea, and ALT increase. This amendment will harmonize the dosing guidance across all clinical trials of abemaciclib in the metastatic setting. The amendment updated the safety language regarding hepatic monitoring, assessment of renal function, and venous thromboembolic events (VTEs) for ongoing patients. Minor typographical and formatting edits were made throughout the document for clarity and consistency.

3.1.4. Rationale for Amendment (d)

Study JPCE protocol was amended to update the dose modification and the safety monitoring guidance for interstitial lung disease (ILD)/pneumonitis. Changes to Section 7.4.1.1 (Dose Adjustments and Delays) and to Table JPCE.7.2 were made to specify dose modifications in response to ILD/pneumonitis. Additional monitoring guidance was included in Section 9.4.2.4. These updates are more conservative than the development core safety information of the current Investigator's Brochure (IB).

Section 7.7 (Concomitant Therapy) was updated to align with the current IB. Additional language was added cautioning against concomitant use of abemaciclib and substrates of the following:

- P-glycoprotein
- breast cancer resistance protein
- organic cation transporter 2
- multidrug and toxin extrusion protein 1 (MATE1), and
- MATE2-K.

Substrates of these transporters, including metformin, digoxin, and dofetilide, should be substituted or avoided.

Section 7.7 (Concomitant Therapy) was also updated to remove cautionary language regarding coadministration of narrow therapeutic index cytochrome P450 (CYP) substrate drugs. This is based on clinical study (Study I3Y-MC-JPCB) that found no meaningful effect of abemaciclib on the pharmacokinetics (PK) of CYP substrates, namely

- caffeine (CYP1A2)
- S-warfarin (CYP2C9)
- midazolam (CYP3A), and
- dextromethorphan (CYP2D6).

The list of inducers and strong inhibitors of CYP3A (Appendix 6) was also modified to reflect updated guidance.

The protocol was updated to bring references to abemaciclib into alignment with the sponsor's standard language.

Minor typographical and formatting edits were made throughout the document for clarity and consistency.

3.1.5. Rationale for Amendment (e)

This amendment to the Study JPCE protocol updates the dose adjustment criteria related to nonhematologic toxicity, ALT/AST, and VTEs to ensure alignment with the current IB. Specifically, protocol updates were made to the dose adjustment section for Nonhematologic Toxicity (Section 7.4.1.1.1.1.2) and Hepatic Toxicity (Section 7.4.1.1.1.1.4), the Venous Thromboembolic Events section was added (Section 7.4.1.1.1.1.6), and corresponding updates were made in Table JPCE.7.2. Additionally, the Concomitant Therapy (Section 7.7) information was updated for CYP3A modulators and transporter substrates. Finally, safety monitoring language in Special Hepatic Safety Data Collection (Section 9.4.2.1) and Venous Thromboembolic Events (Section 9.4.2.3) was updated to align with the current IB, and Interstitial Lung Disease/Pneumonitis (Section 9.4.2.4) was edited for clarity to include specific ILD/pneumonitis Grades requiring abemaciclib discontinuation.

Minor typographical and formatting edits were made throughout the document for clarity and consistency.

3.2. Background

One of the hallmarks of cancer is cell cycle dysregulation, through either overactivity of the proliferative machinery (by means of activating mutations in cyclin-dependent kinases [CDKs], gene amplification in regions of the genome that are responsible for encoding, and overexpression of individual proteins related to cell cycle proteins) or inactivation of the proteins that block the cell cycle (for example, p53 or p16, retinoblastoma complex). These alterations render cells less dependent on mitogenic signaling for proliferation and result in tumor growth.

CDK4 and CDK6 participate in a complex with D-type cyclins to initiate the transition through the G1 restriction point. The CDK4 and CDK6-cyclinD complex regulates the G1 restriction point through phosphorylation of the Rb tumor suppressor protein. Alterations in this pathway occur frequently in a broad spectrum of human cancers and involve 1) loss of CDK inhibitors by mutation or epigenetic silencing, 2) mutation/overexpression of either CDK4 and CDK6 or cyclin D, or 3) inactivation of Rb. Broad spectrums of cancers are potentially sensitive to pharmacologic inhibition of CDK4 and CDK6. From a therapeutic standpoint, the goal of inhibiting CDK4 and CDK6 with a small molecule inhibitor is to prevent cell cycle progression through the G1 restriction point, thus arresting tumor growth. Importantly, synthetic lethal interaction between Kirsten rat sarcoma (*KRAS*) mutation and CDK4 inhibition indicates a potential therapeutic application for CDK4 and CDK6 inhibitors in *KRAS* mutant (*KRAS mt*) NSCLC (Puyol et al. 2010).

Abemaciclib (LY2835219) is a potent and selective small molecule inhibitor of CDK4 and CDK6 that demonstrates antitumor activity in multiple mouse models of human cancer, physical

and PK properties suitable for drug development, and an acceptable toxicity profile in nonclinical species. Abemaciclib administered orally demonstrates single-agent activity and in vitro growth inhibition in multiple human xenograft models, including NSCLC, breast cancer, and ovarian cancer. Several preclinical studies conducted with *KRAS mt* NSCLC models showed greater activity when combined with other cytotoxic or targeted agents, suggesting increased sensitivity to abemaciclib in the presence of activated *KRAS* oncogenes. In addition, abemaciclib showed greater sensitivity in estrogen receptor positive (ER+) breast cancer lines with luminal histology. Cell–based studies, which evaluated the effect of abemaciclib on immune cell activity and metabolism, indicated moderate, not profound, inhibitory activity in T-cells at clinical dose levels. In the Phase 1 study (Study I3Y-MC-JPBA [JPBA]), abemaciclib demonstrated acceptable safety and tolerability as well as evidence of single-agent activity in NSCLC and HR+ breast cancer, including 2 NSCLC patients and 12 HR+ patients with confirmed partial responses (Patnaik et al. 2014).

The importance of intact immune surveillance in controlling outgrowth of neoplastic transformation has been known for decades (Disis 2010). Accumulating evidence shows a correlation between tumor-infiltrating lymphocytes (TILs) in cancer tissue and prognosis in various malignancies (Bremens et al. 2011; Talmadge 2011; Mei et al. 2014). In particular, the presence of CD8+ T cells and the ratio of CD8+ effector T cells/FoxP3+ regulatory T cells seem to correlate with improved prognosis and long-term survival (Liu et al. 2011). Consequently, combining with a drug targeting the programmed death receptor-1 (PD-1)/ programmed death ligand 1 (PD-L1) pathway is attractive for therapeutic intervention in NSCLC.

Pembrolizumab is a potent humanized immunoglobulin G4 (IgG4) monoclonal antibody (mAb) with high specificity of binding to the programmed cell death 1 (PD-1) receptor, thus inhibiting its interaction with programmed cell death ligand 1 (PD-L1) and programmed cell death ligand 2 (PD-L2). Based on preclinical in vitro data, pembrolizumab has high affinity and potent receptor blocking activity for PD-1. Pembrolizumab has an acceptable preclinical safety profile and is in clinical development as an intravenous immunotherapy for advanced malignancies. Keytruda™ (pembrolizumab) is indicated for the treatment of patients across a number of indications.

Refer to the IB/approved labeling for detailed background information.

Comparison of docetaxel to pembrolizumab in patients with NSCLC who had received prior therapy showed better activity with pembrolizumab in both PD-L1 > 50% and PD-L1 1% to 50% (Herbst et al. 2015). Keynote 024 and 042 (clinicaltrials.gov) is testing pembrolizumab monotherapy to chemotherapy in a Phase 3 design in chemo-naïve patients who have either a TPS ≥50% expression or TPS ≥1% PD-L1 expression, respectively. Ongoing clinical trials are being conducted in these tumor types as well as a number of other advanced solid tumor indications and hematologic malignancies. Given the benefit of pembrolizumab in NSCLC a trial to test the addition of abemaciclib in *KRAS mt* patients and in squamous patients is a reasonable developmental step. Also adding pembrolizumab to abemaciclib in breast cancer has clinical relevance in the development of both compounds. The identification of these molecular characteristics and demonstrated clinical activity of abemaciclib and pembrolizumab, provide a therapeutic opportunity to improve clinical benefit beyond what is observed with the current

treatment options available for patients with advanced and/or metastatic NSCLC and HR+, HER2- metastatic breast cancer.

Experiments of murine tumors sensitive to CDK4 and CDK6 inhibitors have also shown intra-tumor dose dependent inflammation as measured by expression of inflammatory genes, including PD-1 gene (CD274). Syngeneic animal models have shown significant increased activity of combined anti-PD-1 treatment with abemaciclib compared with either treatment alone as measured by complete response rates.

It is currently unknown what the impact of CDK4 and CDK6 inhibitors would be on immune effector cells. A second tumor biopsy will be performed after 6 weeks of abemaciclib treatment to assess the impact of abemaciclib on immune effector cells.

3.3. Benefit/Risk Assessment

More information about the known and expected benefits, risks, serious adverse events (SAEs), and reasonably anticipated adverse events (AEs) of abemaciclib are to be found in the IB.

More detailed information about the known and expected benefits and risks of pembrolizumab (Keytruda™) may be found in the following: Patient Information Leaflet, Package Insert, or Summary of Product Characteristics.

4. Objectives and Endpoints

Table JPCE.4.1 shows the objectives and endpoints of the study.

Table JPCE.4.1. Objectives and Endpoints

Objectives	Endpoints
**Primary**	
To characterize further the safety profile of the combination of abemaciclib and pembrolizumab	The safety endpoints per CTCAE v 4.0, NCI 2009 and will include but are not limited to the following:<br>• TEAEs, ECIs, SAEs, and hospitalizations<br>• Clinical laboratory tests, vital signs, and physical examinations
**Secondary**	
To assess the preliminary efficacy of abemaciclib in combination with pembrolizumab	• ORR per tumor assessment using RECIST v1.1<br>• PFS per RECIST v1.1<br>• DoR per RECIST v1.1<br>• DCR per RECIST v1.1<br>• OS
To characterize the PK of abemaciclib and pembrolizumab when given in combination	Abemaciclib and pembrolizumab concentrations in plasma or serum
**Exploratory**	
To assess the preliminary efficacy (per irRECIST) of abemaciclib in combination with pembrolizumab	ORR, PFS, DoR, DCR per tumor assessment using irRECIST
To evaluate biomarkers related to mechanism of action of abemaciclib and/or pembrolizumab, cell cycle, pathogenesis of cancer, immune modulation and assess the relationship between biomarkers and clinical outcomes	Clinical efficacy outcomes, safety outcomes and/or treatment
To evaluate the relationship between abemaciclib or pembrolizumab exposure and response	Drug exposure and efficacy outcomes such as PFS, OS, and safety outcomes such as neutropenia and diarrhea
To evaluate patient self-reported, disease-specific, and treatment-induced symptoms and distal outcomes including pain, the effects on functioning, and quality of life	Assessments by MDASI*

Abbreviations: CTCAE = Common Terminology Criteria for Adverse Events; DCR = disease control rate; DoR= duration of response; ECI = Events of Clinical Interest; irRECIST = immune-related Response Evaluation Criteria in Solid Tumors; MDASI* = MD Anderson Symptom Inventory questionnaire and accompanying supplemental sheet with 2 additional items (worst cough and worst diarrhea); NCI = National Cancer Institute; ORR = objective response rate; OS = overall survival; PFS = progression-free survival; PK = pharmacokinetics; RECIST = Response Evaluation Criteria in Solid Tumors; SAEs = serious adverse events; TEAEs = treatment-emergent adverse events.

5. Study Design

5.1. Overall Design

Study JPCE is a multicenter, nonrandomized, open-label, Phase 1b study of abemaciclib in combination with pembrolizumab in patients with Stage IV NSCLC or HR+, HER2- breast cancer.

Figure JPCE.5.1 illustrates the study design.

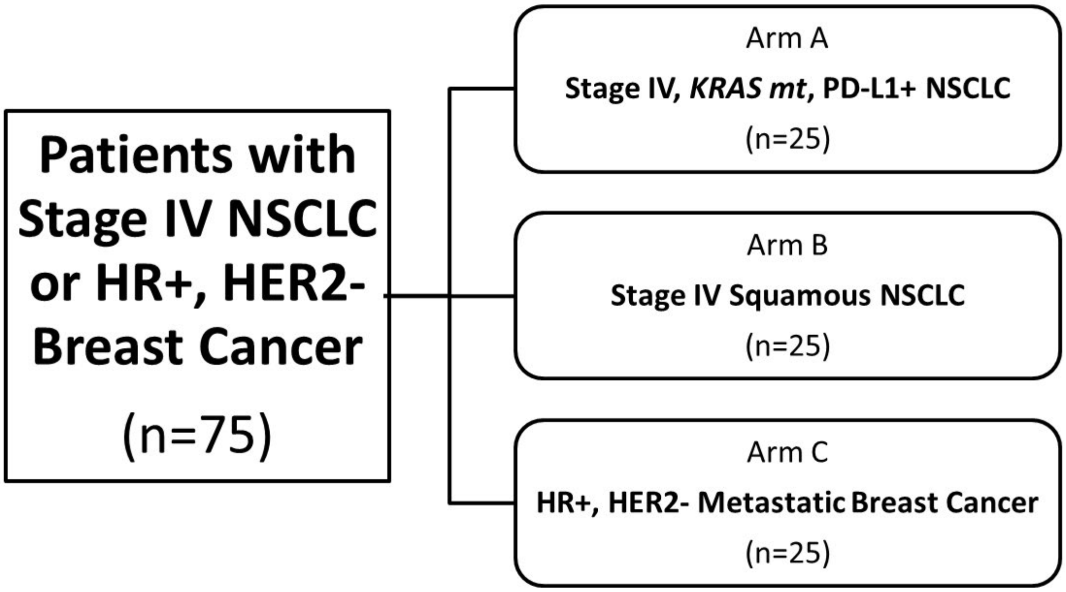

Abbreviations: HER2- = human epidermal growth factor receptor 2 negative; HR+ = hormone receptor positive; *KRAS mt* = Kirsten rat sarcoma mutant; n = number; NSCLC = non-small cell lung cancer; PD-L1 = programmed death-ligand 1(PD-L1+ expression score defined as ≥1%).

Figure JPCE.5.1. Illustration of study design for I3Y-MC-JPCE.

5.2. Number of Patients

Approximately 75 patients with Stage IV NSCLC or HR+, HER2- breast cancer will be enrolled in Study JPCE in Arms A, B, and C. The tumor-specific cohorts will consist of approximately 25 patients with *KRAS mt*, PD-L1+ NSCLC, 25 patients with NSCLC with squamous histology, and 25 patients with HR+, HER2- metastatic breast cancer. An arm may have fewer or greater than 25 patients enrolled.

5.3. End of Study Definition

End of the study is the date of the last visit or last scheduled procedure for the last patient.

5.4. Scientific Rationale for Study Design

Study JPCE is a non-randomized, parallel design in 3 separate cohorts. The primary endpoint is safety for each cohort and there is no control population. Response rate is the secondary endpoint of this study and will be used to screen for significant improvements in activity compared with

historical controls. In NSCLC cohorts, the historical control will be pembrolizumab monotherapy and in breast cancer, it will be abemaciclib monotherapy.

5.5. Justification for Dose

In Study JPCE, the sponsor plans to study abemaciclib administered orally 150 mg every 12 hours (Q12H, [±2 hours]) on Days 1 through 21 of a 21-day cycle. Plasma concentrations achieved by a dose of 150 mg Q12H in Study JPBA were similar to those associated with pRb and topo II alpha inhibition and tumor growth inhibition in the Colo-205 xenograft model (Tate et al. 2014). Furthermore, an abemaciclib dose of 150 mg Q12H at steady state has been shown in Study JPBA to inhibit CDK4 and CDK6, resulting in cell cycle inhibition upstream of the G1 restriction point. The dose of abemaciclib used in Study JPCE was supported by the preliminary safety combination data in Study I3Y-MC-JPBJ (JPBJ). Study JPBJ is an ongoing Phase 1b dose-escalation study of abemaciclib in combination with multiple single-agent options for patients with Stage IV NSCLC including pembrolizumab (Part E). In Study JPBJ Part E, abemaciclib was administered orally at 100 mg and 150 mg Q12H on Days 1 through 21 of a 21-day cycle in combination with pembrolizumab 200 mg administered every 3 weeks (Q3W). The preliminary data from the six patients included in the dose escalation cohorts Study JPBJ Part E, each received pembrolizumab 200 mg intravenous infusion Q3W in combination with abemaciclib at either 100 mg Q12H (n=3) or 150 mg Q12H (n=3) is available. No DLTS were observed in either cohort, and there were 2 Grade 3 TEAEs observed, 1 Grade 3 increased ALT and 1 Grade 3 fatigue which required prolonged hospitalization and defined as SAE. There were no Grade 4 TEAEs observed. It was therefore decided to proceed with the maximum tolerated dose (MTD) confirmation arm of abemaciclib orally at 150 mg every Q12H on Days 1 through 21 of a 21-day cycle in combination with pembrolizumab 200 mg (fixed dose) intravenous infusion on Day 1 of a 21-day cycle. These preliminary results showed that the addition of pembrolizumab to abemaciclib generated no new safety signals. Thus far, no increase in AE frequency or severity in the escalation phase of Part E treatment combination has been detected. The dose escalation results from Study JPBJ Part E suggests the doses administered, abemaciclib given twice daily in combination with pembrolizumab appears to be manageable and tolerable.

The dose of pembrolizumab to be studied in this trial is 200 mg Q3W, which is the dose recently approved in the United States in NSCLC, based on population PK model of pembrolizumab (pembro; MK-3475) in patients treated in KEYNOTE-001 and KEYNOTE-002 (Gangadhar et al. 2015). In KEYNOTE-001, an open-label Phase I study conducted to evaluate the safety, tolerability, PK and pharmacodynamics, and anti-tumor activity of pembrolizumab when administered as monotherapy. The dose escalation portion of this trial evaluated three dose levels, 1 mg/kg, 3 mg/kg and 10 mg/kg, administered every 2 weeks (Q2W) and dose expansion cohorts evaluated 2 mg/kg Q3W and 10 mg/kg Q3W in subjects with advanced solid tumors. All dose levels were well tolerated and no dose-limiting toxicities (DLTs) were observed.

An integrated body of evidence suggests that 200 mg Q3W is expected to provide similar response to 2 mg/kg Q3W, 10 mg/kg Q3W and 10 mg/kg Q2W. Previously, a flat pembrolizumab exposure-response relationship for efficacy and safety has been found in subjects with melanoma in the range of doses between 2 mg/kg and 10 mg/kg. Exposures for 200 mg

Q3W are expected to lie within this range and will be close to those obtained with 2 mg/kg Q3W dose.

In translating to other tumor indications, similarly flat exposure-response relationships for efficacy and safety as observed in subjects with melanoma (KEYNOTE-002) can be expected, as the anti-tumor effect of pembrolizumab is driven through immune system activation rather than through a direct interaction with tumor cells, rendering it independent of the specific tumor type. In addition, available PK results in subjects with melanoma, NSCLC, and other tumor types support a lack of meaningful difference in PK exposures obtained at tested doses among tumor types. Thus the 200 mg Q3W fixed-dose regimen is considered an appropriate fixed dose for other tumor indications as well.

The existing data suggest 200 mg Q3W as the appropriate dose for pembrolizumab. Therefore, pembrolizumab will be administered at a flat dose of 200 mg as an approximate 30-minute intravenous infusion on Day 1 of a 21-day cycle.

6. Study Population

Prospective approval of protocol deviations to recruitment and enrollment criteria, also known as protocol waivers or exemptions, is not permitted.

6.1. Inclusion Criteria

Patients are eligible to be included in the study only if they meet **all** of the following criteria:

[1] have a Stage IV diagnosis of 1 of the following: NSCLC (*KRAS mt*, PD-L1+); NSCLC (squamous histology); or HR+, HER2- breast cancer.

- For Part A: NSCLC, *KRAS mt*, PD-L1+ only:
    - *KRAS* mutation status done by local testing.
    - Have a diagnostic positive PD-L1 expression score (defined as ≥1%) per central testing.
    - Must be chemotherapy-naïve for metastatic NSCLC.
        - Prior therapy in the neoadjuvant or adjuvant setting is not considered as a prior line of systemic chemotherapy unless a patient has progressed ≤6 months since last dose.
- For Part B: NSCLC, squamous subtype only:
    - Must have received only 1 prior therapy containing platinum-based chemotherapy for advanced/metastatic NSCLC.
        - Prior therapy in the neoadjuvant or adjuvant setting is not considered as a prior line of systemic chemotherapy unless a patient has progressed ≤6 months since last dose.
- For Part C: HR+, HER2- breast cancer:
    - Must express at least one of the hormone receptors (HR; estrogen receptor [ER] or progesterone receptor [PgR]) by immunohistochemistry (IHC) to fulfill the requirement for HR+ disease on the primary tumor or metastatic lesion of the breast cancer.
        - ER and PgR assays are considered positive if there is at least 1% positive tumor nuclei in their sample as defined in the relevant American Society of Clinical Oncology (ASCO)/College of American Pathologists (CAP) Guidelines (Hammond et al. 2010).
    - To fulfill the requirement of HER2- disease, a breast cancer must not demonstrate, at initial diagnosis or upon subsequent biopsy, overexpression of HER2 by either IHC or in-situ hybridization (ISH) as defined in the relevant ASCO/CAP guidelines (Wolff et al. 2013).
    - Most recent HR and HER2 receptor testing should be used to determine eligibility.

- Must have previously received prior treatment with at least 1 but no more than 2 chemotherapy regimens in the metastatic setting.

[2] are amenable to provide tumor tissue prior to treatment and provide tumor tissue after treatment initiation (both mandatory).

[3] have presence of measurable disease as defined by RECIST 1.1.

[4] have adequate organ function, as defined below:

**System**	**Laboratory Value**
**Hematologic**	
ANC	≥1.5 × 10^9/L
Platelets	≥100 × 10^9/L
Hemoglobin	≥8 g/dL
**Coagulation**	
PTT or aPTT	<5 seconds above ULN and INR≤1.5 times ULN or PT <5 seconds above ULN. Patients receiving anticoagulant therapy are permitted if the PTT or aPTT and INR or PT is within therapeutic range of intended use of anticoagulants.
**Hepatic**	
Total bilirubin	≤1.5 × ULN
ALT and AST	≤3.0 × ULN **OR**<br>≤5 × ULN if the liver has tumor involvement<br>Patients on Part A with tumor involvement of the liver and ALT or AST >3.0 to ≤5.0 times ULN must have Child-Pugh Class A using the Child-Turcotte scoring system.
**Renal**	
Calculated creatinine clearance (see Appendix 5)	≥45 mL/min

Abbreviations: ALT = alanine aminotransferase; ANC = absolute neutrophil count; aPTT = activated partial thromboplastin time; AST = aspartate aminotransferase; INR = international normalized ratio; PTT = partial thromboplastin time; ULN = upper limit of normal.

[5] have a performance status (PS) ≤1 on the Eastern Cooperative Oncology Group (ECOG; Appendix 8) scale (Oken et al. 1982).

[6] have discontinued all previous treatments for cancer and recovered from the acute effects of therapy. Patients must have discontinued from previous treatments, as shown below:

**Previous Treatment**	**Length of Time Prior to First Dose of Study Treatment**
Chemotherapy	≥21 days for myelosuppressive agents or ≥14 days for nonmyelosuppressive agents, and recovered from the acute effects of therapy (treatment-related toxicity resolved to baseline) except for residual alopecia.
Immunotherapy	
Radiotherapy	
Investigational therapy	
Biologic agents (for example, anti-cancer monoclonal antibodies [mAb])	≥4 weeks (28 days) and recovered from adverse events due to agents

[7] are reliable and willing to be available for the duration of the study and are willing to follow study procedures.

[8] are at least 18 years old at the time of screening.

[9] men must be sterile or agree to use an *effective method of contraception* or a *highly effective method of contraception* during the study and for at least 120 days (4 months) following the last dose of study drug.

Refer to Appendix 1 for definitions of *effective method of contraception* and *highly effective method of contraception*.

[10] women of childbearing potential must:

a. have a negative urine or serum pregnancy test within 72 hours prior to the first dose of study drug (if urine test is positive or cannot be confirmed as negative, a serum pregnancy test will be required), and

b. agree to use a *highly effective method of contraception* during the study and for at least 120 days (4 months) following the last dose of study drug.

Refer to Appendix 1 for the definition of *highly effective method of contraception*.

[11] have an estimated life expectancy of ≥12 weeks.

[12] have given written informed consent prior to any study-specific procedures.

[13] are able to swallow oral medications.

6.2. Exclusion Criteria

Patients will be excluded from the study if they meet **any** of the following criteria:

[14] have a personal history of any of the following conditions: syncope of either unexplained or cardiovascular etiology, ventricular arrhythmia (including but not limited to ventricular tachycardia and ventricular fibrillation), or sudden cardiac arrest.
Exception: subjects with controlled atrial fibrillation for >30 days prior to study treatment are eligible.

[15] have central nervous system (CNS) metastasis with development of associated neurological changes 14 days prior to receiving study drug.

- Untreated CNS metastases are not permitted.
- Screening of asymptomatic patients without history of CNS metastasis is not required.

[16] have a history of any other cancer (except nonmelanoma skin cancer or carcinoma in-situ of the cervix or breast), unless in complete remission with no therapy for a minimum of 3 years.

- Patients enrolling into the NSCLC arms (Arm A or Arm B) with history of carcinoma in-situ of the breast must be off tamoxifen at least 21 days prior to first dose.

[17] is pregnant or breastfeeding or expecting to conceive or father children within the projected duration of the trial, starting with the pre-screening or screening visit through 120 days (4 months) after the last dose of trial treatment.

[18] have a serious concomitant systemic disorder (for example, active infection including human immunodeficiency virus, or cardiac disease) that, in the opinion of the investigator, would compromise the patient's ability to adhere to the protocol.

[19] have QTc interval of >470 msec electrocardiogram (ECG). Results from previous ECGs taken 90 days prior to screening may be used.

[20] have history of interstitial lung disease.

[21] have history of or active autoimmune disease, or other syndrome that requires systemic steroids or autoimmune agents for the past 2 years. Participants with vitiligo, resolved childhood asthma or atopy, hypothyroidism, or Sjogren's syndrome, as well as participants requiring only intranasal steroids, intermittent use of bronchodilators, local steroid injections, or physiologic replacement doses of prednisone (≤10 mg/d) [or its equivalent] are not excluded from this study. Replacement therapy (e.g., thyroxine, insulin, or physiologic corticosteroid replacement therapy for adrenal or pituitary insufficiency) is not considered a form of systemic treatment and is allowed.

[22] have received a live vaccination within 30 days of study start. Seasonal flu vaccines that do not contain live virus are permitted.

[23] have received prior treatment with an anti PD-1, anti PD-L1, anti PD-L2 agent or with an agent directed to another stimulatory or co-inhibitory T-cell receptor (e.g., anti-cytotoxic T-lymphocyte-associated protein 4 [CTLA-4], OX 40, CD137) and was discontinued from that treatment due to a Grade 3 or higher immune-related AE.

[24] are currently enrolled in a clinical trial involving an investigational product, have received treatment within 21 days of the initial dose of study drug with an investigational product or nonapproved use of a drug, or device (other than the study drug/device used in this study) for noncancer indications or any other type of medical research judged not to be scientifically or medically compatible with this study.

[25] have history of hypersensitivity or allergic reactions attributed to compound of similar chemical or biological composition to pembrolizumab.

[26] have previously received treatment with any CDK4 and CDK6 inhibitor.

[27] have active bacterial, fungal and/or known viral infection (for example, human immunodeficiency virus [HIV] antibodies, hepatitis B surface antigen [HBSAg], or hepatitis C antibodies [HCAb]). Screening is not required for enrollment.

[28] have history of or current pneumonitis.

[29] have a known psychiatric or substance abuse disorder that would interfere with the participant's ability to cooperate with the requirements of the study.

[30] have had an allogenic tissue/solid organ transplant.

[31] have a diagnosis of immunodeficiency or is receiving chronic systemic steroid therapy (at a dose exceeding 10 mg daily of prednisone equivalent) or any other form of immunosuppressive therapy within 7 days prior the first dose of study drug.

6.3. Lifestyle Restrictions

Patients will be instructed to refrain from drinking grapefruit juice.

6.4. Screen Failures

Re-screening of individuals who do not meet the criteria for participation in this study is not permitted.

7. Treatments

7.1. Treatments Administered

Table JPCE.7.1 shows the treatment regimens.

Table JPCE.7.1. Treatment Regimens

	Dose and Schedule (q 21 Days)		
**Arm**	**Patients/Cohort**	**Abemaciclib Dose**	**Pembrolizumab Dose**
A	Stage IV NSCLC *KRAS mt*, PD-L1+	150 mg Q12H	200 mg Day 1 of a 21-day cycle
B	Stage IV NSCLC squamous	150 mg Q12H	200 mg Day 1 of a 21-day cycle
C	HR+, HER2- metastatic breast cancer	150 mg Q12H	200 mg Day 1 of a 21-day cycle

Abbreviations: HER2- = human epidermal growth factor receptor 2 negative; HR+ = hormone receptor positive; *KRAS mt* = Kirsten rat sarcoma mutant; NSCLC = non-small cell lung cancer; PD-L1 = programmed death ligand 1; q = every; Q12H = every 12 hours.

Patients will receive 150 mg of abemaciclib orally Q12H in combination with pembrolizumab.

Pembrolizumab 200 mg (fixed dose) will be administered as an approximate 30-minute intravenous infusion on Day 1 of a 21-day cycle. Sites should make every effort to target infusion timing to be as close to 30 minutes as possible. However, given the variability of infusion pumps from site to site, a window between -5 minutes and +10 minutes is permitted (that is, infusion time can be 25 to 40 minutes). During Cycles 1 and 2, infusions will be followed by a 1-hour observation period. Signs and symptoms of infusion-related reactions usually develop during or shortly after drug infusion and generally resolve completely within 24 hours of completion of infusion. If there is no evidence of an infusion-related reaction, no observation period is required for subsequent treatment cycles unless clinically indicated.

During Cycles 1 through 8, when abemaciclib is scheduled to be administered on the same day as pembrolizumab and PK samples are drawn, abemaciclib should be given immediately after the initiation of the pembrolizumab infusion.

The investigator or his/her designee is responsible for the following:

- explaining the correct use of the drugs and planned duration of each individual's treatment to the patient/study site personnel/legal representative
- verifying that instructions are followed properly
- maintaining accurate records of study treatment dispensing and collection
- at the end of the study returning all unused medication to Lilly, or its designee, unless Lilly and sites have agreed all unused medication is to be destroyed by the site, as allowed by local law

7.1.1. Packaging and Labelling

All study treatments will be provided by Lilly. Clinical trial materials will be labeled according to the country's regulatory requirements.

7.2. Method of Treatment Assignment

7.2.1. Selection and Timing of Doses

A cycle is defined as an interval of 21 days. A delay of a cycle due to holiday, weekend, bad weather, or other unforeseen circumstances will be permitted for a maximum of 7 days and will not be counted as a protocol deviation.

A patient may continue to receive study treatment until he or she meets 1 or more of the specified reasons for discontinuation (as described in Section 8).

7.3. Blinding

This is an open-label study.

7.4. Dosage Modification

7.4.1. Special Treatment Considerations

7.4.1.1. Dose Adjustments and Delays

Table JPCE.7.2 presents dose adjustments to occur if the event was related to abemaciclib. Any questions related to dose adjustments may be discussed with the Lilly clinical research physician (CRP).

Table JPCE.7.2. Toxicity Dose Adjustments and Delays of Abemaciclib

Toxicity Type	Toxicity Profile and Severity	Dose Suspension	Dose Reduction
Hematologic Toxicity<br>Section 7.4.1.1.1.1.1	Grade 3	Dose **MUST** be suspended until toxicity resolves to at least Grade 2.	Dose **MAY** be reduced by 1 dose level - investigator's discretion.
Hematologic Toxicity<br>Section 7.4.1.1.1.1.1	Recurrent Grade 3	Dose **MUST** be suspended until toxicity resolves to at least Grade 2.	Dose **MUST** be reduced by 1 dose level.
Hematologic Toxicity<br>Section 7.4.1.1.1.1.1	Grade 4	Dose **MUST** be suspended until toxicity resolves to at least Grade 2.	Dose **MUST** be reduced by 1 dose level.
Hematologic toxicity: **If patient requires administration of blood cell growth factors**<br>Sections 7.4.1.1.1.1.1 and 7.7.1	Regardless of severity.<br>(Use of growth factors according to ASCO Guidelines)	Dose **MUST** be suspended for at least 48 hours after the last dose of blood cell growth factors was administered and until toxicity resolves to at least Grade 2.	Dose **MUST** be reduced by 1 dose level unless already performed for incidence of toxicity that led to the use of growth factor.
Nonhematologic Toxicity[a] (except diarrhea, ALT/AST increased, ILD/pneumonitis, and VTE[e])<br>Section 7.4.1.1.1.1.2	Persistent or recurrent[b] Grade 2 that does not resolve with maximal supportive measures within 7 days to baseline or Grade 1	Dose **MUST** be suspended until toxicity resolves to either baseline or Grade 1.	Dose **MUST** be reduced by 1 dose level.
Nonhematologic Toxicity[a] (except diarrhea, ALT/AST increased, ILD/pneumonitis, and VTE[e])<br>Section 7.4.1.1.1.1.2	Grade 3 or 4	Dose **MUST** be suspended until toxicity resolves to either baseline or Grade 1.	Dose **MUST** be reduced by 1 dose level.
Diarrhea<br>Sections 7.4.1.1.1.1.3 and 7.7.1.1.1	Grade 2 that does not resolve within 24 hours to at least Grade 1	Dose **MUST** be suspended until toxicity resolves to at least Grade 1.	Dose reduction **NOT** required.
	Persistent or recurrent[b] Grade 2 that does not resolve with maximal supportive measures or any Grade of diarrhea that requires hospitalization	Dose **MUST** be suspended until toxicity resolves to at least Grade 1.	Dose **MUST** be reduced by 1 dose level.
	Grade 3 or 4	Dose **MUST** be suspended until toxicity resolves to at least Grade 1.	Dose **MUST** be reduced by 1 dose level.
ALT/AST Increased<br>Sections 7.4.1.1.1.1.4 and 9.4.2.1	Persistent or recurrent[b] Grade 2 (>3.0-5.0×ULN)[c], or<br>Grade 3 (>5.0-20.0×ULN)[d]	Dose **MUST** be suspended until toxicity resolves to baseline or Grade 1.	Dose **MUST** be reduced by 1 dose level.
	Grade 4 (>20.0×ULN)	Abemaciclib therapy **MUST** be discontinued.	Abemaciclib therapy **MUST** be discontinued.

Toxicity Dose Adjustments and Delays of Abemaciclib

Toxicity Type	Toxicity Profile and Severity	Dose Suspension	Dose Reduction
ALT/AST Increased with increased total bilirubin, in the absence of cholestasis Sections 7.4.1.1.1.1.4 and 9.4.2.1	≥Grade 2 increased ALT/AST (>3.0 x ULN) with total bilirubin >2 x ULN	Abemaciclib therapy **MUST** be discontinued.	Abemaciclib therapy **MUST** be discontinued.
ILD/Pneumonitis Sections 7.4.1.1.1.1.5 and 9.4.2.4	Grade 2	Dose **MUST** be suspended until toxicity resolves to baseline or Grade ≤1. If persisting despite maximal supportive measures for more than 7 days, permanently discontinue abemaciclib.	Dose **MUST** be reduced by 1 dose level.
	Recurrent Grade 2 or Grade 3-4	Abemaciclib therapy **MUST** be discontinued.	Abemaciclib therapy **MUST** be discontinued.
VTE[e] Sections 7.4.1.1.1.1.6 and 9.4.2.3	Grade 3 or 4	Suspend dose and treat as clinically indicated. May resume abemaciclib therapy when participant is clinically stable.	Suspend dose and treat as clinically indicated. May resume abemaciclib therapy when participant is clinically stable.

Abbreviations: ALT = alanine aminotransferase; ASCO = American Society of Clinical Oncology; AST = aspartate aminotransferase; ILD =interstitial lung disease; ULN = upper limit of normal; VTE = venous thromboembolic events.

Note: MAY = per the investigator's clinical judgment; MUST = mandatory.

[a] Additional guidance for renal and hepatic monitoring is in Sections 9.4.2.1 and 9.4.2.2.

[b] Determination of persistent events will be at the discretion of the investigator. Recurrent toxicity refers to the same event occurring within the next 8 weeks (as measured from the stop date of the preceding event). As a general guidance, based on the risk/benefit balance assessment per the investigator, for a patient who experiences a new episode of Grade 3 hematological toxicity after more than 8 weeks following the last episode of same Grade 3 hematological toxicity, the investigator may consider resuming the patient on the same drug dose should the patient satisfy the following conditions:

- shows stable hematological counts (Grade ≤2) during that timeframe
- has absence of any signs or risk of infection
- is benefiting from study treatment

[c] Note: the patient who presents with no liver metastases at baseline.

[d] Grade 3 ALT/AST increased is a trigger for additional assessments and possibly hepatic monitoring. See Section 9.4.2.1 for additional guidance for hepatic monitoring.

[e] VTE dose modifications are specifically for patients with breast cancer. Dose modifications for patients with lung cancer who experience VTEs should be managed using the nonhematologic toxicity guidance.

7.4.1.1.1. Dose Adjustments

7.4.1.1.1.1. Abemaciclib

Dose adjustments as outlined in Table JPCE.7.3 are allowed both within a cycle and between cycles. Abemaciclib must be reduced sequentially by one dose level.

Abemaciclib dose alterations (omission, reduction, discontinuation) should not be based solely on the presentation of serum creatine values, because these may not reflect actual renal function. Further information can be found in the abemaciclib IB Section 3.2.4.

For patients requiring dose reduction(s), any re-escalation to a prior dose level is permitted only after consultation with the Lilly CRP. After re-escalation, subsequent dose adjustments should be based on the dose of abemaciclib that the patient is currently receiving.

Table JPCE.7.3. Dose Adjustments of Abemaciclib

Dose Adjustment	Oral Dose	Frequency
0	150 mg	Q12H
1	100 mg	Q12H

Abbreviation: Q12H = every 12 hours.

If a patient receiving the 100 mg Q12H dose of abemaciclib requires a further dose reduction, the patient should be discontinued from study treatment. If a patient is, in the judgment of the investigator, receiving clinical benefit from study therapy and requires further dose reduction than what is outlined in Table JPCE.7.3, the investigator must discuss with the Lilly CRP prior to any further dose reduction.

7.4.1.1.1.1.1. Hematologic Toxicity

Before the start of each cycle, hematologic toxicity possibly related to abemaciclib must resolve to at least Grade 2. If a patient experiences Grade 4 hematologic toxicity possibly related to abemaciclib, then dosing must be suspended (until the toxicity resolves to at least Grade 2) and the dose of abemaciclib must be reduced by 1 dose level as outlined in Table JPCE.7.2. If a patient experiences Grade 3 hematologic toxicity, then dosing must be suspended (until the toxicity resolves to at least Grade 2) and the dose of abemaciclib may be reduced by 1 dose level as outlined in Table JPCE.7.2 at the discretion of the investigator. If the patient experiences a recurrent episode of Grade 3 hematologic toxicity, then dosing must be suspended (until the toxicity resolves to at least Grade 2) and the dose of abemaciclib must be reduced by 1 dose level as outlined in Table JPCE.7.2.

Recurrent toxicity refers to the same event occurring within the next 8 weeks (as measured from the stop date of the preceding event). As a general guidance, based on the risk/benefit balance assessment per the investigator, for a patient who experiences a new episode of Grade 3 hematological toxicity after more than 8 weeks following the last episode of same Grade 3 hematological toxicity, the investigator may consider resuming the patient on the same drug dose should the patient satisfy the following conditions:

- The patient showed stable hematological counts (Grade ≤2) during that timeframe
- In the absence of any signs or risk of infection
- The patient is benefiting from study treatment

If a patient requires administration of blood cell growth factors, the dose of abemaciclib must be suspended for at least 48 hours after the last dose of blood cell growth factors was administered and until toxicity resolves to at least Grade 2, then must be reduced by 1 dose level, if a dose

reduction for the specific event necessitating the use of the growth factors has not already occurred.

7.4.1.1.1.1.2. Nonhematological Toxicity

Before the start of each cycle, nonhematologic toxicity (except alopecia and fatigue) possibly related to abemaciclib must resolve to either baseline or at least Grade 1. If a patient experiences ≥Grade 3 nonhematologic toxicity (except diarrhea, refer to Section 7.4.1.1.1.1.3; or ALT/AST increased, refer to Section 7.4.1.1.1.1.4; or ILD/pneumonitis, refer to Section 7.4.1.1.1.1.5; or VTE, refer to Section 7.4.1.1.1.1.6) possibly related to abemaciclib, then dosing must be suspended (until the toxicity resolves to either baseline or Grade 1) and the dose of abemaciclib must be reduced by 1 dose level as outlined in Table JPCE.7.2.

If a patient experiences persistent or recurrent Grade 2 nonhematologic toxicity (except diarrhea, refer to Section 7.4.1.1.1.1.3; or ALT/AST increased, refer to Section 7.4.1.1.1.1.4; or ILD/pneumonitis, refer to Section 7.4.1.1.1.1.5; or VTE, refer to Section 7.4.1.1.1.1.6) possibly related to abemaciclib that does not resolve with maximal supportive measures within 7 days to either baseline or Grade 1, then dosing must be suspended (until the toxicity resolves to either baseline or Grade 1) and the dose of abemaciclib must be reduced by 1 dose level as outlined in Table JPCE.7.2.

7.4.1.1.1.1.3. Diarrhea

A patient experiencing diarrhea requiring hospitalization (irrespective of grade that is, requiring intravenous rehydration) or severe diarrhea (Grade 3 or 4; see Appendix 9) must have study treatment suspended (until the toxicity resolves to either baseline or at least Grade 1) and the dose of abemaciclib must be reduced by 1 dose level as outlined in Table JPCE.7.2. If a patient experiences Grade 2 diarrhea that does not resolve with maximal supportive measures (refer to Section 7.7.1.1.1) within 24 hours to at least Grade 1, the study drug must be suspended (until the toxicity resolves to at least Grade 1) but abemaciclib dose reduction is not required. However, if a patient experiences persistent or recurrent Grade 2 diarrhea that does not resolve with maximal supportive measures (refer to Section 7.7.1.1.1) within 24 hours to at least Grade 1, then study treatment must be suspended (until the toxicity resolves to at least Grade 1) and the dose of abemaciclib must be reduced by 1 dose level as outlined in Table JPCE.7.2.

7.4.1.1.1.1.4. Hepatic Toxicity

Dose modifications and management for increased ALT/AST are provided in Table JPCE.7.2. For persistent or recurrent Grade 2 ALT/AST increase that does not resolve with maximal supportive measures within 7 days to baseline or Grade 1, or Grade 3 ALT/AST increase, abemaciclib must be suspended until the toxicity has resolved to baseline or Grade 1 and the dose must be reduced by 1 dose level. Discontinue abemaciclib for ≥Grade 2 increased ALT/AST (>3.0 x ULN) with total bilirubin (TBL) >2 x ULN, in the absence of cholestasis. For Grade 4 ALT/AST increase, the patient must be discontinued from abemaciclib. Refer to Section 9.4.2.1 for additional hepatic monitoring guidance.

7.4.1.1.1.1.5. Interstitial Lung Disease/Pneumonitis

Dose modifications and management of ILD/pneumonitis are provided in Table JPCE.7.2. If a patient experiences Grade 2 ILD/pneumonitis, then dosing must be suspended until the toxicity resolves to either baseline or Grade 1, and the dose of abemaciclib must be reduced by 1 dose level. If Grade 2 ILD/pneumonitis does not resolve with maximal supportive measures within 7 days to baseline or Grade 1, then abemaciclib must be discontinued.

For recurrent Grade 2 or Grade 3 or 4 ILD/pneumonitis, a patient must be discontinued from abemaciclib treatment. Refer to Section 9.4.2.4 for additional ILD/pneumonitis monitoring guidance.

7.4.1.1.1.1.6. Venous Thromboembolic Events

Dose modifications and management for VTE for patients with breast cancer are provided in Table JPCE.7.2. For Grade 3 or 4 VTE, suspend abemaciclib dose and treat as clinically indicated. Abemaciclib therapy may be resumed when participant is clinically stable. Dose modifications for patients with lung cancer who experience VTEs should be managed using the nonhematologic toxicity guidance. Refer to Section 9.4.2.3 for additional VTE monitoring guidance.

7.4.1.1.1.2. Pembrolizumab

Adverse events (AEs; both nonserious and serious) associated with pembrolizumab exposure may represent an immunologic etiology. These AEs may occur shortly after the first dose or several months after the last dose of treatment. Pembrolizumab must be withheld (<u>dose reductions are not permitted</u>) for drug-related toxicities and severe or life-threatening AEs (Table JPCE.7.5). See Section 7.7.1.2 for supportive care guidelines for pembrolizumab, including use of corticosteroids (Section 7.4.1.1.2.2).

7.4.1.1.2. Dose Delays and Omission

7.4.1.1.2.1. Abemaciclib

Table JPCE.7.4. General Dose Delays of Abemaciclib

General Dose Delays	Time Permitted for Delay
Delay of a cycle due to holidays, weekends, bad weather, or other unforeseen circumstances	7 days
Toxicity-time for recovery to baseline or at least Grade 1 for nonhematologic and at least Grade 2 for hematologic toxicity	Up to 14 days

In exceptional cases, a dose delay >14 days is permitted upon agreement between the investigator and the Lilly clinical research physician.

7.4.1.1.2.2. Pembrolizumab

Table JPCE.7.5. Dose Modification Guidelines for Pembrolizumab

Toxicity	Hold Treatment For Grade	Timing for Restarting Treatment	Treatment Discontinuation
Diarrhea/Colitis	2-3	Toxicity resolves to Grade 0-1	Toxicity does not resolve within 12 weeks of last dose or inability to reduce corticosteroid to 10 mg or less of prednisone or equivalent per day within 12 weeks
	4	Permanently discontinue	Permanently discontinue
AST, ALT, or Increased Bilirubin (see Section 9.4.2)	2	Toxicity resolves to Grade 0-1	Toxicity does not resolve within 12 weeks of last dose
	3-4	Permanently discontinue (see exception below)[a]	Permanently discontinue
Type 1 diabetes mellitus (if new onset) or Hyperglycemia	T1DM or 3-4	Hold pembrolizumab for new onset Type 1 diabetes mellitus or Grade 3-4 hyperglycemia associated with evidence of beta cell failure	Resume pembrolizumab when patients are clinically and metabolically stable
Hypophysitis	2-4	Toxicity resolves to Grade 0-1. Therapy with pembrolizumab can be continued while endocrine replacement therapy is instituted	Toxicity does not resolve within 12 weeks of last dose or inability to reduce corticosteroid to 10 mg or less of prednisone or equivalent per day within 12 weeks
Hyperthyroidism	3	Toxicity resolves to Grade 0-1	Toxicity does not resolve within 12 weeks of last dose or inability to reduce corticosteroid to 10 mg or less of prednisone or equivalent per day within 12 weeks
	4	Permanently discontinue	Permanently discontinue
Hypothyroidism	2-4	Therapy with pembrolizumab can be continued while thyroid replacement therapy is instituted	Therapy with pembrolizumab can be continued while thyroid replacement therapy is instituted
Infusion Reaction	2[b]	Toxicity resolves to Grade 0-1	Permanently discontinue if toxicity develops despite adequate premedication
	3-4	Permanently discontinue	Permanently discontinue
Pneumonitis	2	Toxicity resolves to Grade 0-1	Toxicity does not resolve within 12 weeks of last dose or inability to reduce corticosteroid to 10 mg or less of prednisone or equivalent per day within 12 weeks
	3-4 or recurrent 2	Permanently discontinue	Permanently discontinue
Renal Failure or Nephritis	2	Toxicity resolves to Grade 0-1	Toxicity does not resolve within 12 weeks of last dose or inability to reduce corticosteroid to 10 mg or less of prednisone or equivalent per day within 12 weeks
	3-4	Permanently discontinue	Permanently discontinue

Dose Modification Guidelines for Pembrolizumab

Toxicity	Hold Treatment For Grade	Timing for Restarting Treatment	Treatment Discontinuation
Myocarditis	1-2	Toxicity resolves to Grade 0-1	Toxicity does not resolve within 12 weeks of last dose or inability to reduce corticosteroid to 10 mg or less of prednisone or equivalent per day within 12 weeks
	3-4	Permanently discontinue	Permanently discontinue
All Other Immune-Related Toxicity	Intolerable or persistent 2	Toxicity resolves to Grade 0-1	Toxicity does not resolve within 12 weeks of last dose or inability to reduce corticosteroid to 10 mg or less of prednisone or equivalent per day within 12 weeks
	3	Toxicity resolves to Grade 0-1 or permanently discontinue based on the type of event.	Toxicity does not resolve within 12 weeks of last dose or inability to reduce corticosteroid to 10 mg or less of prednisone or equivalent per day within 12 weeks. Events that require discontinuation include and not limited to: Guillain-Barre Syndrome, encephalitis
	4 or recurrent 3	Permanently discontinue	Permanently discontinue

Note: Permanently discontinue for any severe or Grade 3 (Grade 2 for pneumonitis) drug-related AE that recurs or any life-threatening event.

[a] For patients with liver metastasis who begin treatment with Grade 2 AST or ALT, if AST or ALT increases by greater than or equal to 50% relative to baseline and lasts for at least 1 week, then patients should be discontinued.

[b] If symptoms resolve within 1 hour of stopping drug infusion, the infusion may be restarted at 50% of the original infusion rate (e.g., from 100 mL/hr to 50 mL/hr). Otherwise, dosing will be held until symptoms resolve and the subject should be premedicated for the next scheduled dose. Refer to Table JPCE.7.6 – Infusion Treatment Guidelines for further management details.

Dosing interruptions of pembrolizumab are permitted. Patients should be placed back on study therapy within 3 weeks of the scheduled interruption and reason for interruption should be documented in the electronic case report form (eCRF).

Regardless of dose delays for abemaciclib, the date of the pembrolizumab administration shall constitute Day 1 of the each cycle.

7.5. Preparation/Handling/Storage/Accountability

Abemaciclib will be supplied by Lilly for oral administration. Abemaciclib should be stored according to the temperature range listed on the product label, and should not be opened, crushed, or chewed. Patients should store abemaciclib in the original package provided and be instructed to keep all medication out of reach of children.

Pembrolizumab is a commercially available product. Investigators should consult the manufacturer’s instructions for complete packaging, labeling, storage, and stability information.

There are 2 formulations of pembrolizumab:

- Pembrolizumab is supplied as a single-use 50-mg lyophilized powder per vial. This lyophilized powder is reconstituted with sterile water for injection prior to use. The buffer contains L-histidine, polysorbate 80 as surfactant, sucrose as stabilizer/tonicity modifier, and hydrochloric acid (HCl) and/or sodium hydroxide (NaOH) for pH adjustment if necessary.

- Pembrolizumab Solution for Infusion is a sterile, non-pyrogenic aqueous solution supplied in a single-use Type I glass vial containing 100 mg/4 mL of pembrolizumab. The product is a preservative-free and latex free solution, which is essentially free of extraneous particulates. Pembrolizumab Solution for Infusion vials are filled to a target of 4.25 mL (106.25 mg) to ensure recovery of 4.0 mL (100 mg).

7.6. Treatment Compliance

Patient compliance with abemaciclib will be assessed by counting returned solid oral dosage units. Study medication administration data will be recorded in the patient's medical record and eCRF.

Patients who are significantly noncompliant will be discontinued from the study. A patient will be considered significantly noncompliant if he or she misses 7 or more consecutive days of abemaciclib (full doses), or more than 25% cumulative days of abemaciclib (full doses) during the study. Similarly, a patient will be considered significantly noncompliant if he or she is judged by the investigator to have intentionally or repeatedly taken more than the prescribed amount of medication. Abemaciclib dose suspensions or delays related to toxicity may occur and will not result in a patient being considered as noncompliant.

Pembrolizumab will be administered only at the investigational sites by authorized study site personnel. As a result, treatment compliance is ensured.

7.7. Concomitant Therapy

Appropriate documentation of all forms of premedications, supportive care, concomitant medications, and supplements must be captured at each visit in the eCRF. Concomitant medications and supportive care therapies must also be documented at the time of discontinuation and at the 30-day short-term follow-up visit.

Modulators of CYP3A

Abemaciclib is extensively metabolized through oxidation by CYP3A. In clinical drug interaction studies,

- coadministration of clarithromycin, a strong CYP3A inhibitor, increased exposure (area under the concentration-time curve) to abemaciclib by 3.4-fold (Study I3Y-MC-JPBE), and
- coadministration of rifampin, a strong CYP3A inducer, decreased exposure to abemaciclib by 95% (Study I3Y-MC-JPBF).

Strong inhibitors of CYP3A (given via non-topical routes of administration) should be substituted or avoided if possible (Appendix 6). This includes grapefruit or grapefruit juice. In particular, avoid oral administration of the very strong CYP3A inhibitor, ketoconazole.

If coadministration with a strong CYP3A inhibitor is unavoidable, investigators should reduce the dose of abemaciclib by 50 mg at the start of CYP3A inhibitor treatment. That is, for patients receiving 150 mg twice daily, reduce the dose to 100 mg twice daily. For patients who have already dose reduced to 100 mg twice daily for tolerability, reduce the dose further to 50 mg twice daily. Alternatively, the investigator may consider suspending abemaciclib for the duration of the CYP3A inhibitor medication. Dose suspensions ≥28 days must be discussed with Lilly CRP/CRS.

Upon discontinuation of the strong CYP3A inhibitor, the dose of abemaciclib may be re-escalated to the dose that was used before starting the strong inhibitor after a sufficient washout period (3-5 half-lives of the strong inhibitor). Re-escalation of the abemaciclib dose requires review and approval from Lilly CRP/CRS.

Inducers of CYP3A should be substituted or avoided if possible (Appendix 6). Coadministration with a CYP3A inducer ≥28 days must be discussed with Lilly CRP/CRS.

Transporter Substrates

At clinically relevant concentrations, abemaciclib inhibits the transporters P-glycoprotein, breast cancer resistance protein, organic cation transporter 2 (OCT2), multidrug and toxin extrusion protein 1 (MATE1), and MATE2-K. The observed serum creatinine increase in clinical studies with abemaciclib is likely due to inhibition of tubular secretion of creatinine via OCT2, MATE1, and MATE2-K. In vivo interactions of abemaciclib with narrow therapeutic index substrates of these transporters, such as digoxin and dabigatran, may occur.

Radiation Therapy

Radiation therapy is not permitted during the study while receiving pembrolizumab.

Note: Radiation therapy to a symptomatic solitary lesion may be allowed at the investigator's discretion in the absence of disease progression per RECIST 1.1.

Live Vaccines

Live vaccines within 30 days prior to the first dose of study treatment and while participating in the study are not permitted. Examples of live vaccines include, but are not limited to, the following: measles, mumps, rubella, varicella/zoster, yellow fever, rabies, Bacillus of Calmette and Guerin strain of *Mycobacterium bovis*, and typhoid vaccine. Seasonal influenza vaccines for injection are generally killed virus vaccines and are allowed; however, intranasal influenza vaccines (e.g., FluMist®) are live attenuated vaccines and are not allowed.

7.7.1. Supportive Care

Patients will receive supportive care as judged by their treating physician, if necessary. Supportive care measures may include, but are not limited to, antidiarrheal agents, antiemetic

agents, opiate and nonopiate analgesic agents, appetite stimulants (except for megestrol acetate), and granulocyte and erythroid growth factors. Details of interventions (for example, medications such as sedatives, antibiotics, analgesics, antihistamines, steroids, or erythroid-stimulating agents), procedures (for example, paracentesis or thoracentesis), or blood products (for example, blood cells, platelets, or fresh frozen plasma transfusions) should be recorded on the eCRFs.

If it is unclear whether a therapy should be regarded as supportive care, the investigator should consult the Lilly CRP. Use of any supportive care therapy should be reported on the eCRFs. Guidelines regarding the use of other specific supportive care agents are presented below.

7.7.1.1. Abemaciclib

7.7.1.1.1. Supportive Management of Diarrhea

At enrollment, patient should receive instructions on the management of diarrhea. In the event of diarrhea, supportive measures should be initiated as early as possible. These include the following:

- At the first sign of loose stools, the patient should initiate anti-diarrheal therapy (for example, loperamide) and notify the investigator/site for further instructions and appropriate follow-up.
- Patients should also be encouraged to drink fluids (for example, 8 to 10 glasses of clear liquids per day).
- Site personnel should assess response within 24 hours.
- If diarrhea does not resolve with anti-diarrheal therapy within 24 hours to either baseline or Grade 1, study drug should be suspended until diarrhea is resolved to baseline or Grade 1.
- When study drug recommences, dosing should be adjusted as outlined in Table JPCE.7.2 and Section 7.4.1.1.1.1.

In cases of significant diarrhea, Grade 2 through 4 (Appendix 9), which has not responded to interventions as outlined above, if the investigators are considering the addition of steroids to treat potential colitis, the sponsor strongly recommends an endoscopic procedure to document colitis prior to initiating steroids.

In severe cases of diarrhea, the measuring of neutrophil counts and body temperature and proactive management of diarrhea with antidiarrheal agents should be considered.

If diarrhea is severe (requiring intravenous rehydration) and/or associated with fever or severe neutropenia, broad-spectrum antibiotics such as fluoroquinolones must be prescribed.

Patients with severe diarrhea or any grade of diarrhea associated with severe nausea or vomiting should be carefully monitored and given intravenous fluid (IV hydration) and electrolyte replacement.

7.7.1.2. Pembrolizumab

Patients should receive appropriate supportive care measures as deemed necessary by the treating investigator. Suggested supportive care measures for the management of AEs with potential immunologic etiology are outlined below. Where appropriate, these guidelines include the use of oral or intravenous treatment with corticosteroids as well as additional anti-inflammatory agents if symptoms do not improve with administration of corticosteroids. Note that several courses of steroid tapering may be necessary as symptoms may worsen when the steroid dose is decreased. For each disorder, attempts should be made to rule out other causes such as metastatic disease or bacterial or viral infection, which might require additional supportive care. The treatment guidelines are intended to be applied when the investigator determines the events to be related to pembrolizumab.

Note: If after the evaluation the event is determined not to be related, the investigator does not need to follow the treatment guidance (as outlined below). Refer to Section 7.4.1.1.2.2 for dose modification.

Pneumonitis:

- For **Grade 2 events**, treat with systemic corticosteroids. When symptoms improve to Grade 1 or less, steroid taper should be started and continued over no less than 4 weeks.
- For **Grade 3 or 4 events**, immediately treat with intravenous steroids. Administer additional anti-inflammatory measures, as needed.
- Add prophylactic antibiotics for opportunistic infections in the case of prolonged steroid administration.

Diarrhea Colitis:

Patients should be carefully monitored for signs and symptoms of enterocolitis (such as diarrhea, abdominal pain, blood or mucus in stool, with or without fever) and of bowel perforation (such as peritoneal signs and ileus).

- All subjects who experience diarrhea/colitis should be advised to drink liberal quantities of clear fluids. If sufficient oral fluid intake is not feasible, fluid and electrolytes should be substituted via intravenous infusion. For Grade 2 or higher diarrhea, consider gastrointestinal consultation and endoscopy to confirm or rule out colitis.
- For **Grade 2 diarrhea/colitis**, administer oral corticosteroids.
- For **Grade 3 or 4 diarrhea/colitis**, treat with intravenous steroids followed by high-dose oral steroids.
- When symptoms improve to Grade 1 or less, steroid taper should be started and continued over no less than 4 weeks.

Type 1 diabetes mellitus (T1DM; if new onset, including diabetic ketoacidosis [DKA]) or ≥ Grade 3 hyperglycemia, if associated with ketosis (ketonuria) or metabolic acidosis (DKA):

- For **T1DM** or **Grade 3 or 4** Hyperglycemia

  - Insulin replacement therapy is recommended for T1DM and for Grade 3 or 4 hyperglycemia associated with metabolic acidosis or ketonuria.
  - Evaluate patients with serum glucose and a metabolic panel, urine ketones, glycosylated hemoglobin, and C-peptide.

Hypophysitis:

- For **Grade 2** events, treat with corticosteroids. When symptoms improve to Grade 1 or less, steroid taper should be started and continued over no less than 4 weeks. Replacement of appropriate hormones may be required as the steroid dose is tapered.
- For **Grade 3 or 4** events, treat with an initial dose of intravenous corticosteroids followed by oral corticosteroids. When symptoms improve to Grade 1 or less, steroid taper should be started and continued over no less than 4 weeks. Replacement of appropriate hormones may be required as the steroid dose is tapered.

Hyperthyroidism or Hypothyroidism:
Thyroid disorders can occur at any time during treatment. Monitor patients for changes in thyroid function (at the start of treatment, periodically during treatment, and as indicated based on clinical evaluation) and for clinical signs and symptoms of thyroid disorders.

- **Grade 2** hyperthyroidism events (and **Grade 2 through 4** hypothyroidism):
  - In hyperthyroidism, non-selective beta-blockers (for example, propranolol) are suggested as initial therapy.
  - In hypothyroidism, thyroid hormone replacement therapy, with levothyroxine or liothyronine, is indicated per standard of care.
- **Grade 3 or 4** hyperthyroidism
  - Treat with an initial dose of intravenous corticosteroid followed by oral corticosteroids. When symptoms improve to Grade 1 or less, steroid taper should be started and continued over no less than 4 weeks. Replacement of appropriate hormones may be required as the steroid dose is tapered.

Hepatic:

- For **Grade 2** events, monitor liver function tests more frequently until returned to baseline values (consider weekly).
  - Treat with intravenous or oral corticosteroids
- For **Grade 3 or 4** events, treat with intravenous corticosteroids for 24 to 48 hours.
- When symptoms improve to Grade 1 or less, a steroid taper should be started and continued over no less than 4 weeks.

Renal Failure or Nephritis:

- For **Grade 2 through 4** events, treat with systemic corticosteroids.
- When symptoms improve to Grade 1 or less, steroid taper should be started and continued over no less than 4 weeks.

Management of Infusion Reactions:

Signs and symptoms usually develop during or shortly after drug infusion and generally resolve completely within 24 hours of completion of infusion. See Table JPCE.7.6.

Table JPCE.7.6. Infusion Reaction Treatment Guidelines

NCI CTCAE Grade	Treatment	Premedication at subsequent dosing
Grade 1<br>Mild reaction; infusion interruption not indicated; intervention not indicated	Increase monitoring of vital signs as medically indicated until the subject is deemed medically stable in the opinion of the investigator.	None
Grade 2<br>Requires therapy or infusion interruption but responds promptly to symptomatic treatment (e.g., antihistamines, NSAIDS, narcotics, intravenous fluids); prophylactic medications indicated for ≤24 hrs	**Stop infusion and monitor symptoms.**<br>Additional appropriate medical therapy may include but is not limited to:<br>• Intravenous fluids<br>• Antihistamines<br>• NSAIDS<br>• Acetaminophen<br>• Narcotics<br>Increase monitoring of vital signs as medically indicated until the subject is deemed medically stable in the opinion of the investigator.<br>If symptoms resolve within 1 hour of stopping drug infusion, the infusion may be restarted at 50% of the original infusion rate (e.g., from 100 mL/hr to 50 mL/hr). Otherwise dosing will be held until symptoms resolve and the subject should be premedicated for the next scheduled dose.<br>**Subjects who develop Grade 2 toxicity despite adequate premedication should be permanently discontinued from further trial treatment administration.**	Subject may be premedicated 1.5 h (± 30 minutes) prior to infusion of pembrolizumab with:<br>Diphenhydramine 50 mg po (or equivalent dose of antihistamine).<br>Acetaminophen 500-1000 mg po (or equivalent dose of antipyretic).
Grades 3 or 4<br>Grade 3:<br>Prolonged (i.e., not rapidly responsive to symptomatic medication and/or brief interruption of infusion); recurrence of symptoms following initial improvement; hospitalization indicated for other clinical sequelae (e.g., renal impairment, pulmonary infiltrates)<br>Grade 4:<br>Life-threatening; pressor or ventilatory support indicated	**Stop infusion.**<br>Additional appropriate medical therapy may include but is not limited to:<br>• Intravenous fluids<br>• Antihistamines<br>• NSAIDS<br>• Acetaminophen<br>• Narcotics<br>• Oxygen<br>• Pressors<br>• Corticosteroids<br>• Epinephrine*<br>Increase monitoring of vital signs as medically indicated until the subject is deemed medically stable in the opinion of the investigator.<br>Hospitalization may be indicated.<br>*In cases of anaphylaxis, epinephrine should be used immediately.<br>**Subject is permanently discontinued from further trial treatment administration.**	No subsequent dosing
Appropriate resuscitation equipment should be available in the room and a physician readily available during the period of drug administration.		

Abbreviations: CTCAE = Common Terminology Criteria for Adverse Events; NCI = National Cancer Institute; NSAID = non-steroid anti-inflammatory drug; po = orally.

7.8. Treatment after the End of the Study

Study completion will occur following the final analysis of OS, which will occur approximately 1 year after last patient entered treatment in the study, as determined by Lilly.

Investigators will continue to follow Schedule of Activities (Section 2) for all patients until notified by Lilly that study completion has occurred.

7.8.1. Continued Access

Patients who are still on study treatment at the time of study completion may continue to receive study treatment if they are experiencing clinical benefit and no undue risks.

Continued access period will apply to this study only if at least 1 patient is still on abemaciclib when study completion occurs. Lilly will notify investigators when the continued access period begins.

The patient's continued access to abemaciclib will end when a criterion for discontinuation is met (Section 8). Continued access follow-up will begin the day after the patient and the investigator agree to discontinue abemaciclib and lasts approximately 30 (±7) days. Follow-up procedures will be performed as shown in the Continued Access Schedule of Activities (Table JPCE.2.4).

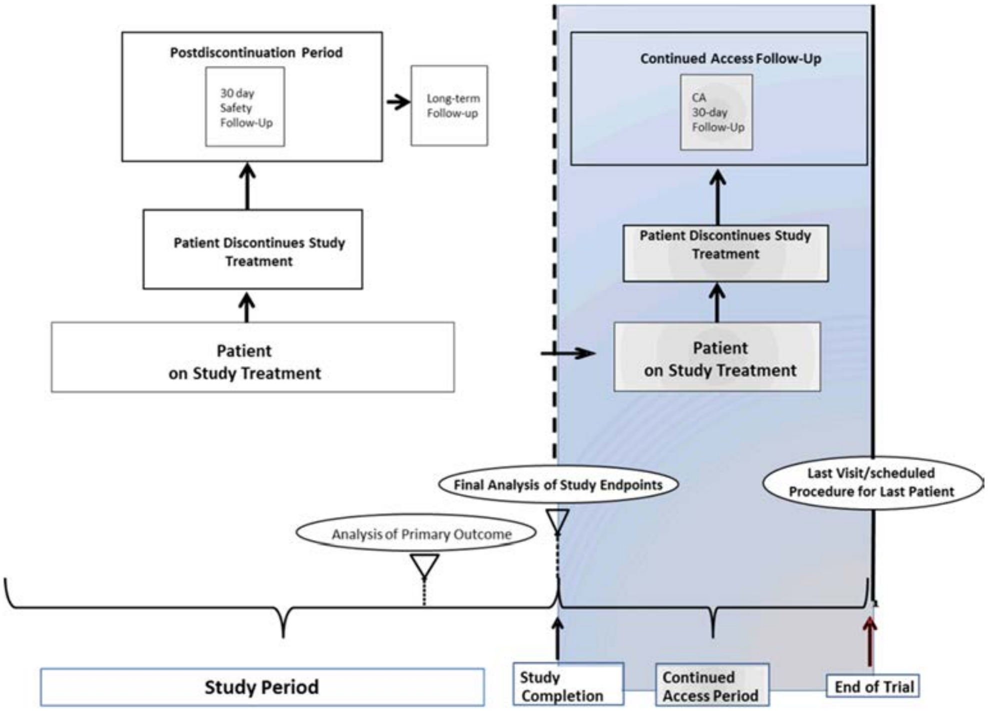

Abbreviation: CA = continued access.

Figure JPCE.7.1. Continued access diagram.

Patients who are in short-term follow-up when the continued access period begins will continue in short-term follow-up until the 30-day short-term follow-up visit is completed. Long-term follow-up does not apply.

Patients who are in long-term follow-up when the continued access period begins will be discontinued from long-term follow-up.

8. Discontinuation Criteria

8.1. Discontinuation from Study Treatment

Patients will be discontinued from study treatment in the following circumstances:

- the patient is enrolled in any other clinical trial involving an investigational product or any other type of medical research judged not to be scientifically or medically compatible with this study
- the patient becomes pregnant during the study. See Section 9.2 regarding regulatory reporting requirements on fetal outcome and breastfeeding.
- the patient is significantly noncompliant with study procedures and/or treatment
- disease progression
  - Patients may remain on study treatment with repeat imaging >4 weeks to access tumor response or confirmed progression per irRECIST to account for unique tumor response seen with immunotherapeutic drugs
- unacceptable toxicity
- the patient has had 1 dose reduction and experiences an AE that would cause a second dose reduction
- the patient, for any reason, requires treatment with another therapeutic agent that has been demonstrated to be effective for treatment of the study indication. Discontinuation from study treatment will occur prior to introduction of the new agent.
- the investigator decides that the patient should be discontinued from study treatment
- the patient requests to be discontinued from study treatment
- the patient's designee (for example, parents, legal guardian, or caregiver) requests that the patient be discontinued from study treatment

Patients who are discontinued from study treatment will have follow-up procedures performed as shown in the Schedule of Activities (Section 2).

For patients who discontinue study treatment without objectively measured progressive disease, continue to evaluate tumor response according to planned tumor assessment schedule by the same method used at baseline and throughout the follow-up periods until patient has objective disease progression or until the study's final analysis of OS. After the patient has objective disease progression, radiologic tests are no longer required.

8.1.1. Discontinuation of Inadvertently Enrolled Patients

If Lilly or the investigator identifies a patient who did not meet enrollment criteria and was inadvertently enrolled, a discussion must occur between the Lilly CRP and the investigator to determine if the patient may continue in the study. If both agree it is medically appropriate to continue, the investigator must obtain documented approval from the Lilly CRP to allow the inadvertently enrolled patient to continue in the study with or without study treatment.

8.2. Discontinuation from the Study

Patients will be discontinued from the study in the following circumstances:

- participation in the study needs to be stopped for medical, safety, regulatory, or other reasons consistent with applicable laws, regulations, and good clinical practice (GCP)
- the patient becomes pregnant during the study. See Section 9.2 regarding regulatory reporting requirements on fetal outcome and breastfeeding.
- the investigator decides that the patient should be discontinued from the study
- the patient requests to be discontinued from the study
- the patient's designee (for example, parents, legal guardian, or caregiver) requests that the patient be discontinued from the study

Patients who discontinue from the study early will have follow-up procedures performed as shown in the Schedule of Activities (Section 2).

8.3. Lost to Follow-Up

A patient will be considered lost to follow-up if he or she repeatedly fails to return for scheduled visits and is unable to be contacted by the study site. Study site personnel are expected to make diligent attempts to contact patients who fail to return for a scheduled visit or who the site is otherwise unable to follow-up.

Study site personnel will attempt to collect the survival status for all enrolled patients who are lost to follow-up, including enrolled patients who do not receive study treatment, within legal and ethical boundaries. Public sources may be searched for survival status information. If the patient's survival status is determined, the survival status will be documented, and the patient will not be considered lost to follow-up.

Lilly personnel will not be involved in any attempts to collect survival status information.

9. Study Assessments and Procedures

Section 2 provides the Schedule of Activities for this study.

Appendix 3 provides a list of the laboratory tests that will be performed for this study.

Appendix 4 provides the schedule for collection of samples in this study.

Unless otherwise stated in the following subsections, all samples collected for specified laboratory tests will be destroyed within 60 days after receipt of confirmed test results. Certain samples may be retained for a longer period, if necessary, to comply with applicable laws, regulations, or laboratory certification standards.

9.1. Efficacy Assessments

Tumor response will be assessed every 6 weeks (± 7 days) by investigator, with confirmatory assessment (including disease progression) obtained at the next routine scheduled imaging time point (that is, after 6 weeks ±7 days). For the tumor expansion phase, after 48 weeks, tumor assessments will be conducted every 9 weeks. The same method of assessment and technique used at baseline should be used for each consecutive assessment thereafter.

See Section 10.3.2 for definitions of the efficacy endpoints.

9.1.1. Appropriateness of Assessments

The measures used to assess safety and efficacy in this study are consistent with those used in most conventional oncology trials.

9.2. Adverse Events

The investigator will use Common Terminology Criteria for Adverse Events (CTCAE) Version 4.0 (NCI 2009) to assign AE terms and severity grades.

Investigators are responsible for:

- monitoring the safety of patients in this study and for alerting Lilly or its designee to any event that seems unusual, even if this event may be considered an unanticipated benefit to the patient
- the appropriate medical care of patients during the study
- documenting their review of each laboratory safety report
- following, through an appropriate health care option, AEs that are serious or otherwise medically important, considered related to study treatment or the study, or that caused the patient to discontinue study treatment before completing the study. The patient should be followed until the event resolves, stabilizes with appropriate diagnostic evaluation, or is reasonably explained. Frequency of follow-up evaluation is left to the discretion of the investigator.

Progression of the cancer under study is not considered an AE unless it is considered to be drug-related by the investigator.

After the ICF is signed, study site personnel will record via eCRF the occurrence and nature of each patient's preexisting conditions, including clinically significant signs and symptoms of the disease under treatment in the study. In addition, study site personnel will record via eCRF any change in the preexisting conditions and any new conditions as AEs. Investigators should record their assessment of the potential relatedness of each AE to study procedure or study treatment via eCRF. From the time of treatment allocation through 30 days following cessation of treatment, all AEs must be reported by the investigator.

The investigator will record all relevant AE/SAE information in the CRF.

The investigator will interpret and document whether or not an AE has a reasonable possibility of being related to study treatment or a study procedure, taking into account the disease, concomitant treatments, or pathologies. A "reasonable possibility" means that there is a cause and effect relationship between the study treatment and/or study procedure and the AE.

Planned surgeries and nonsurgical interventions should not be reported as AEs unless the underlying medical condition has worsened during the course of the study.

Study site personnel must report any dose modifications or treatment discontinuations that result from AEs to Lilly or its designee via eCRF, clarifying, if possible, the circumstances leading to the dose modification or discontinuation of treatment.

Adverse events meeting either of the below criteria, although not serious per International Council for Harmonisation (ICH) definition, are reportable in the same timeframe as SAEs to meet certain local requirements.

Therefore, these events need to be reported within 24 hours:

- is a new cancer (that is not a condition of the study);
- is associated with an overdose.

9.2.1. Serious Adverse Events

An SAE is any AE from this study that results in one of the following outcomes:

- death
- initial or prolonged inpatient hospitalization
- a life-threatening experience (that is, immediate risk of dying)
- persistent or significant disability/incapacity
- congenital anomaly/birth defect
- considered significant by the investigator for any other reason: important medical events that may not result in death, be life-threatening, or require hospitalization may be considered serious, based upon appropriate medical judgment.

Although all AEs after signing the ICF are recorded in the eCRF, SAE reporting begins after the patient has signed the ICF and has received study treatment. If an SAE occurs after signing the ICF, but prior to receiving study treatment, it needs to be reported ONLY if it is considered reasonably possibly related to study procedure.

Study site personnel must notify Lilly or its designee of any SAE within 24 hours of investigator awareness of the event via a Lilly-approved method. If alerts are issued via telephone, they are to be immediately followed with official notification on study-specific SAE forms. This 24-hour notification requirement refers to the initial SAE information and all follow-up SAE information.

For the time period beginning at treatment allocation through 90 days following cessation of treatment, or 30 days following cessation of treatment if subject initiates new anticancer therapy, whichever is earlier, any SAE, or follow up to a SAE, including death due to any cause other than progression of cancer, whether or not related to the study treatment, must be reported within 24 hours via a Lilly-approved method.

Pregnancy (during maternal or paternal exposure to study treatment) does not meet the definition of an AE but should be reported. To fulfill regulatory requirements, any pregnancy should be reported from the time of treatment allocation through 120 days following cessation of treatment, or 30 days following cessation of treatment if patient initiates new anticancer therapy, whichever is earlier, must be reported by the investigator to collect data on the outcome for both mother and fetus.

Investigators are not obligated to actively seek AEs or SAEs for patients once they have discontinued and/or completed the study (the patient summary CRF has been completed). However, if the investigator learns of any SAE, including a death, at any time after a patient has been discharged from the study, and he/she considers the event reasonably possibly related to the study treatment or study participation, the investigator must promptly notify Lilly.

Planned hospitalizations or procedures for preexisting conditions that were recorded in the patient's medical history at the time of enrollment should not be considered SAEs. Hospitalization or prolongation of hospitalization without a precipitating clinical AE (for example, for the administration of study treatment or other protocol-required procedure) should not be considered SAEs.

Serious adverse events, including death, caused by disease progression should not be reported unless the investigator deems them to be possibly related to study treatment.

9.2.1.1. Events of Clinical Interest - Pembrolizumab

The select non-serious and serious adverse events below, also known as Events of Clinical Interest (ECI), must be reported to the sponsor.

An ECI is defined as:

1) an overdose of pembrolizumab (defined as ≥1000 mg [5 times the dose] of pembrolizumab) that is not associated with clinical symptoms or abnormal laboratory values or
2) an elevated aspartate aminotransferase (AST) or alanine aminotransferase (ALT) laboratory value that is greater than or equal to 3 times the upper limit of normal (ULN) and an elevated total bilirubin laboratory value that is greater than or equal to 2 times the ULN, and at the same time, an alkaline phosphatase laboratory value that is less than

2 times the ULN, as determined by way of protocol-specific laboratory testing or unscheduled laboratory testing.

Events of Clinical Interest (ECIs) identified from the date of first dose through 90 days following discontinuation of study treatment, or 30 days after the initiation of a new anticancer therapy, whichever is earlier, need to be reported to the sponsor within 24 hours.

9.2.2. Suspected Unexpected Serious Adverse Reactions

Suspected unexpected serious adverse reactions (SUSARs) are serious events that are not listed in the IB and that the investigator identifies as related to study treatment or study procedure. United States 21 CFR 312.32 and European Union Clinical Trial Directive 2001/20/EC and the associated detailed guidances or national regulatory requirements in participating countries require the reporting of SUSARs. Lilly has procedures that will be followed for the recording and expedited reporting of SUSARs that are consistent with global regulations and associated detailed guidances.

9.2.3. Complaint Handling

Lilly collects product complaints on investigational products used in clinical studies in order to ensure the safety of study participants, monitor quality, and to facilitate process and product improvements.

Patients will be instructed to contact the investigator as soon as possible if he or she has a complaint or problem with the investigational product so that the situation can be assessed.

9.3. Treatment of Overdose

Refer to the IB and/or Product Label (abemaciclib and/or pembrolizumab).

9.4. Safety

9.4.1. Other Safety Measures

For each patient, ECGs, vital signs, laboratory tests, or other tests should be collected as shown in the Schedule of Activities (Section 2). Electrocardiograms should be recorded according to the study-specific recommendations.

Blood samples will be collected to determine whether patients meet inclusion/exclusion criteria and to monitor patient health. Enrollment and treatment decisions may be based upon results performed locally. If local laboratory tests are used for this purpose, then a duplicate specimen must be submitted to the central laboratory. Discrepancies between local and central laboratory that may have an impact on eligibility or treatment decisions will not be considered protocol deviations.

Investigators must document their review of each laboratory safety report.

Any clinically significant findings that result in a diagnosis and that occur after the patient receives the first dose of study treatment should be reported to Lilly or its designee as an AE via eCRF.

9.4.2. Safety Monitoring

Lilly will periodically review evolving aggregate safety data within the study by appropriate methods.

To ensure patient safety and comply with regulatory guidance, the investigator is to consult with the Lilly CRP regarding collection of specific recommended clinical information and follow-up laboratory tests. Refer to the Event of Clinical Interest (ECI) Guidance for Potential DILI (Drug-Induced Liver Injury) in Clinical Trials supplement provided.

9.4.2.1. Special Hepatic Safety Data Collection

Close Hepatic Monitoring and Evaluation

Liver testing (Appendix 10), including ALT, AST, ALP, TBL, direct bilirubin, gamma-glutamyl transferase, and creatine kinase, should be repeated within 2 to 4 days to confirm the abnormality and to determine if it is increasing or decreasing, if one or more of these conditions occur:

If a participant with baseline results of ...	develops the following elevations:
ALT or AST <1.5x ULN	ALT or AST ≥3x ULN
ALT or AST ≥1.5x ULN	ALT or AST ≥2x baseline

Abbreviations: ALT = alanine aminotransferase; AST = aspartate aminotransferase; ULN = upper limit of normal.

If the abnormality persists or worsens, clinical and laboratory monitoring and evaluation for possible causes of abnormal liver tests, should be initiated by the investigator in consultation with the Lilly-designated medical monitor. At a minimum, this evaluation should include physical examination and a thorough medical history, including symptoms, recent illnesses (for example, heart failure, systemic infection, hypotension, or seizures), history of concomitant medications (including over-the-counter, herbal and dietary supplements, history of alcohol drinking and other substance abuse). In addition, the evaluation should include a blood test for prothrombin time (PT-INR); serological tests for viral hepatitis A, B, C, E, autoimmune hepatitis; and an abdominal imaging study (for example, ultrasound or CT scan).

Additional Hepatic Safety Data Collection

Additional safety data should be collected via the adverse event form in the CRF if 1 or more of the following conditions occur:

In participants enrolled with baseline ALT/AST <1.5 ULN:

- Elevation of serum ALT/AST to ≥5x ULN on 2 or more consecutive blood tests.
- The combination of elevated ALT/AST ≥3x ULN and elevated TBL ≥2x ULN.

In participants enrolled with baseline ALT/AST ≥1.5x ULN:

- Elevated ALT/AST ≥3x baseline on 2 or more consecutive tests.

- The combination of elevated ALT/AST ≥2x baseline and elevated TBL ≥2x ULN.

In all study participants:

- Discontinuation from study treatment due to a hepatic event or abnormality of liver tests.
- Occurrence of a hepatic event considered to be an SAE.

9.4.2.2. Renal Function

Abemaciclib has been shown to increase serum creatinine due to inhibition of renal tubular secretion of creatinine without affecting cystatin C-calculated glomerular filtration rate. Increases in serum creatinine occurred within the first 2 weeks of treatment, remained stable through the treatment period, and were reversible upon treatment discontinuation. If deterioration of renal function is suspected, serum creatinine should not be the only measure used to assess a patient's renal function.

Dose adjustment (omission, reduction, or discontinuation) should not solely be based on interpretation of serum creatinine values because these may not reflect renal function. If deterioration of renal function is suspected per the investigator's clinical assessment, dose alteration should follow the protocol guidance for non-hematological toxicities (Table JPCE.7.2).

9.4.2.3. Venous Thromboembolic Events

VTE has been identified as an adverse drug reaction for abemaciclib in combination with endocrine therapy. In the randomized Phase 3 studies in patients with breast cancer treated with abemaciclib in combination with endocrine therapy (ET), a greater number of patients experienced VTEs in the abemaciclib plus ET arm than in the placebo plus ET arm or ET alone arm. The majority of patients who experienced VTEs were treated with anticoagulants. At this time, the mechanism underlying the association between abemaciclib and the occurrence of VTEs is not known. Monitor patients for signs and symptoms of deep vein thrombosis and pulmonary embolism and treat as medically appropriate.

9.4.2.4. Interstitial Lung Disease/Pneumonitis

Interstitial lung disease (ILD)/pneumonitis has been identified as an adverse drug reaction for abemaciclib and pembrolizumab. The risk of ILD/pneumonitis in patients receiving abemaciclib in combination with pembrolizumab seems higher and more severe than reported previously for patients receiving abemaciclib with or without anastrozole or pembrolizumab monotherapy. Fatal cases of ILD/pneumonitis have been reported in patients treated with abemaciclib in combination with pembrolizumab with or without anastrozole monotherapy.

Ask your patients to report any new or worsening pulmonary symptoms such as dyspnea, cough, and fever, and investigate and treat as per your local clinical practice (including corticosteroids as appropriate). If ILD/pneumonitis is suspected, investigations may include imaging, such as high-resolution computed tomography, broncheoalveolar lavage, and biopsy as clinically indicated.

In the event of ILD/pneumonitis, consider the following:

Grade 1:

- Reassess history and physical (H&P) and pulse oximetry (resting and with ambulation) every 1 to 2 weeks
- Computer tomography (CT) scan with contrast if clinically indicated or worsening of baseline pulmonary symptoms.

Grade 2:

- Monitor H&P and pulse oximetry (resting and with ambulation) every 3 to 7 days
- Chest CT scan with contrast for evaluation and repeat in 3 to 4 weeks or sooner if clinically indicated
- Pulmonary consultation
- Infectious workup and bronchoscopy with bronchoalveolar lavage prior to initiation of treatment
- Empiric antibiotics
- Start prednisone/methylprednisolone
- If there is no symptomatic improvement after 48 to 72 hours of steroid use, treat the event as Grade 3.

Recurrent Grade 2, Grade 3 or 4:

- Inpatient care
- Infectious workup, and pulmonary and infectious disease consultation
- Start intravenous methylprednisolone
- Consider adding any of the following if there is no improvement after 48 hours:
  - infliximab
  - intravenous immunoglobulin (IVIg)
  - mycophenolate mofetil.

Refer to Table JPCE.7.2 and Table JPCE.7.5 for guidance on dose modification of abemaciclib and pembrolizumab for patients with ILD/pneumonitis (see Appendix 9 for ILD/pneumonitis CTCAE grades). Discontinue abemaciclib in cases of recurrent Grade 2 or severe (Grade 3 or 4) ILD/pneumonitis.

9.5. Pharmacokinetics

Pharmacokinetic samples will be collected as shown in Appendix 4.

Blood samples will be used to determine the concentrations of abemaciclib and its metabolites and pembrolizumab.

Bioanalytical samples collected to measure abemaciclib and pembrolizumab concentration will be retained for a maximum of 1 year following the last patient visit for the study.

9.6. Pharmacodynamics

See Section 9.8.

Samples collected will be identified by the patient number (coded) and retained at a facility selected by Lilly for a maximum of 15 years following the last patient visit for the study.

9.7. Pharmacogenomics

9.7.1. Samples for Pharmacogenetic Research

A whole blood sample will be collected for pharmacogenetic analysis as specified in Appendix 4, where local regulations allow.

Samples will not be used to conduct unspecified disease or population genetic research, either now or in the future. Samples will be used to investigate variable response to study treatment and to investigate genetic variants thought to play a role in cancer. Assessment of variable response may include evaluation of AEs or differences in efficacy.

All samples will be coded with the patient number. These samples and any data generated can be linked back to the patient only by the study site personnel. Samples will be retained for a maximum of 15 years after the last patient visit for the study, or for a shorter period if local regulations and/or ethical review boards (ERBs)/institutional review boards (IRBs) impose shorter time limits, at a facility selected by Lilly or its designee. This retention period enables use of new technologies, response to questions from regulatory agencies, and investigation of variable response that may not be observed until later in the development of abemaciclib or after abemaciclib becomes commercially available or while pembrolizumab is commercially available.

Molecular technologies are expected to improve during the 15-year storage period and therefore cannot be specifically named. However, existing technologies include whole genome and exome sequencing, genome-wide association studies, multiplex assays, candidate gene studies, and epigenetic analyses. Regardless of the technology utilized, data generated will be used only for the specific research scope described in this section.

9.8. Biomarkers

Biomarker research is performed to address questions of relevance to drug disposition, target engagement, pharmacodynamics, mechanism of action, variability of patient response (including safety), and clinical outcome. Sample collection is incorporated into clinical studies to enable examination of these questions through measurement of biomolecules including deoxyribonucleic acid (DNA), ribonucleic acid (RNA), proteins, lipids, and other cellular elements.

As part of Lilly's ongoing efforts to understand the relationship between cancer, genetics, and response to therapy, this study will analyze biomarkers relevant to abemaciclib or pembrolizumab, cell cycle, immune modulation, and/or cancer and/or for related research methods or validation of diagnostic tools or assays.

Samples for biomarker research will be collected as specified in Appendix 4, where local regulations allow. It is possible that biomarker data for patients in the study has already been generated from samples that were collected and analyzed prior to enrolling in this trial. This may include data generated from genetic analyses. If available, these data may be requested from medical records for use in the research described in Sections 9.8.1 and 9.8.2.

9.8.1. Samples for Nonpharmacogenetic Biomarker Research

Plasma, serum, and whole blood (flow/RNA/DNA) samples for nonpharmacogenetic biomarker research will be collected as specified in Appendix 4 where local regulations allow.

Samples will be examined for biomarkers related to cancer, variable response to abemaciclib or pembrolizumab, the mechanism of action of study drugs, and/or for research-related methods, or validating diagnostic tools or assays.

All samples will be coded with the patient number. These samples and any data generated can be linked back to the patient only by the study site personnel.

Samples will be retained for a maximum of 15 years after the last patient visit for the study, or for a shorter period if local regulations and/or ERBs/IRBs impose shorter time limits, at a facility selected by Lilly or its designee. This retention period enables use of new technologies, response to questions from regulatory agencies, and investigation of variable response that may not be observed until later in the development of abemaciclib or after abemaciclib becomes commercially available or while pembrolizumab is commercially available.

9.8.2. Tissue Samples for Disease Confirmation and Research

Tumor tissue will be examined for biomarkers related to cancer, abemaciclib, pembrolizumab, cell cycle, and/or immune modulation.

Collection of the following tumor tissue samples are **required** for all patients in order to participate in this study (see Appendix 4 for more details):

- a biopsy specimen collected during screening (newly obtained),
- on-treatment biopsy specimen collected Day 1 of Cycle 3

Formalin-fixed paraffin-embedded tumor tissue obtained from the primary tumor or metastatic site should be provided as a block or unstained slides. Due diligence should be used to make sure that tumor sample (not a normal adjacent or a tumor margin sample) is provided. Pathology report accompanying archival tissue may also be requested. The report must be coded with the patient number. Personal identifiers, including the patient's name and initials, must be removed from the institutional pathology report prior to submission. Archival blocks will be sectioned and returned to the study site. Slides and tissue samples collected on-study will not be returned.

Samples will be retained for a maximum of 15 years after the last patient visit for the study, or for a shorter period if local regulations and/or ERBs/IRBs impose shorter time limits, at a facility selected by Lilly or its designee. This retention period enables use of new technologies, response to questions from regulatory agencies, and investigation of variable response that may not be observed until later in the development of abemaciclib or after abemaciclib becomes commercially available.

Technologies are expected to improve during the 15-year storage period and therefore cannot be specifically named. However, existing technologies, including mutation profiling, copy number variability, gene expression, multiplex assays, and/or immunohistochemistry may be performed

on these tissue samples to assess potential associations with these biomarkers and clinical outcomes.

In addition to the required biopsies and biomarker samples discussed in Sections 9.8.1 and 9.8.2, patients may be asked to undergo collection of an additional biopsy specimen and blood sample after treatment with study drugs has been initiated, including potentially after disease progression. Such additional biopsies are optional and should only be performed if clinically feasible. If these additional samples are requested, they will be used to further investigate biomarkers that may explain treatment response and resistance mechanisms.

9.9. Health Economics

Patient-reported outcomes will be measured using the standardized MDASI 19-item core questionnaire (Cleeland et al. 2000). An accompanying supplemental sheet will include 2 additional symptoms items thought to be particularly relevant to this study: a single-item worst cough assessment and a single-item worst diarrhea assessment. The MDASI questionnaire is a 19-item instrument that includes 13 symptom items (pain, fatigue, nausea, disturbed sleep, distress [emotional/upset], shortness of breath, remembering things, lack of appetite, drowsy [sleepy], dry mouth, sad, vomiting, numbness or tingling) and 6 interference items (general activity, mood, work, relations with others, walking, and enjoyment with life). The addition of cough and diarrhea bring the item total for symptoms from 13 up to 15, and the total number of items from 19 to 21.

Responses for the MDASI are captured through the use of 11-point numeric rating scales anchored at 0 (not present or does not interfere) and ranged through 10 (as bad as you can imagine or completely interferes). The MDASI recall period is 24 hours, and typical completion time for this instrument is less than 5 minutes.

Single-items will be individually evaluated for clinically important change. The use of an 11-point, 0 to 10 numeric rating scale (NRS) facilitates the identification of meaningful change, based on a substantial amount of supporting literature. Multi-item symptom and interference scores will be considered as exploratory to assess the concepts of symptom and interference burden.

The self-reported questionnaires will be administered as shown in the Schedule of Activities (Section 2) in countries where the questionnaires have been translated into the native language of the region and linguistically validated. This study will rely on a paper-and-pencil-based administration and patients will complete the questionnaire at the beginning of the visit at baseline and on Day 1 of each treatment cycle, beginning with Cycle 1. Every effort should be made to collect this information during the short-term follow-up visit (that is, Visit 801).

Resource use will be collected during the Day 1 visit, beginning at Cycle 2. Data elements of concern will include, but will not be limited to, the following: hospitalizations (with lengths of stay), emergency room visits and any other clinic visits (for example, urgent care facilities), transfusions, growth factors, and the use of concomitant medication. An appropriate CRF will be developed to facilitate capture of this information. The intent of this effort is to capture sufficient

information to adequately characterize the resource use and economic burden associated with study treatment.

10. Statistical Considerations

10.1. Sample Size Determination

The primary objective of this study is to further evaluate safety and tolerability of abemaciclib in combination with pembrolizumab after the Phase 1a Study JPBJ. The sample size of Study JPCE was selected to allow adequate assessment of safety at the recommended doses for each tumor type.

Approximately 75 treated patients (approximately 25 per cohort for *KRAS mt*, PD-L1+ NSCLC, NSCLC with squamous histology, and HR+, HER2- metastatic breast cancer). A sample size of n=25 and n=75 (combining the 3 cohorts) will provide an 80% confidence interval (CI) of (0, 8.8%) or (0, 3.0%) for the rate of occurrence of an unobserved AE. In addition, for tumor response, under each cohort, with a total sample size of n=25, the 80% CI of the observed response rate is approximately equal to the observed incidence rate +/- 10% to 15%. Example point estimates of incidence rates and corresponding 2-sided 80% CIs are summarized in Table JPCE.10.1. The values are provided as a reference for estimation rather than a basis of any decision criteria.

Table JPCE.10.1. Estimated Incidence Rate and Its 2-Sided 80% CI

**Num. of Cases (N=25)**	**Estimate Rate**	**80% CI***	
		Lower Limit	Upper Limit
**0**	0.0	0.0	0.09
**3**	0.12	0.04	0.25
**5**	0.20	0.10	0.34
**8**	0.32	0.20	0.47
**11**	0.44	0.30	0.59
**14**	0.56	0.41	0.70
**17**	0.68	0.53	0.80
**20**	0.80	0.66	0.90

Abbreviation: CI = confidence interval.
* 80% Clopper-Pearson interval for binomial distribution with sample size of 25

10.2. Populations for Analyses

The following populations will be defined for this study:

Safety population: Includes patients who have received at least one dose of study drug. The safety population will be used for all safety, efficacy, and resource utilization analyses.

10.3. Statistical Analyses

10.3.1. General Consideration

Statistical analysis of this study will be the responsibility of Lilly.

An analysis of primary outcome on safety and efficacy will occur after all patients have completed (or discontinued from) approximately 24 weeks of treatment (Section 10.3.3). The updated analysis may occur based on data collected for approximately 12 months after the last patient enters treatment in each arm. The analyses of each arm may be updated based on the final data lock of the study approximately 12 months after the last patient enters treatment in the study. At this time, the clinical study report for all arms may be written.

Descriptive statistics will be derived where appropriate. The safety and efficacy results will be summarized by cohort; dose exposure and density for each study drug will be calculated by each cohort.

Any change to the data analysis methods described in the protocol will require an amendment ONLY if it changes a principal feature of the protocol.

Any other change to the data analysis methods described in the protocol, and the justification for making the change, will be described in the clinical study report.

Additional exploratory analyses of the data will be conducted as deemed appropriate.

10.3.2. Efficacy Analyses

The objective response rate (ORR), disease control rate (DCR), duration of response (DoR), PFS per investigator assessment and the corresponding CI, will be provided by cohort (according to RECIST v1.1 for disease progression). Time-to-event variables, such as DoR, PFS, and OS, will be estimated by Kaplan-Meier (1958) methodology by cohort. Presentations of preliminary efficacy may include patients enrolled in DLT Phase 1a with the same tumor type and dosing scheme. Individual changes in the tumor burden over time will be presented graphically within a tumor type (that is, Waterfall plots). Subgroup analysis of interest will be further defined in the Statistical Analysis Plan (SAP). The details of PFS censoring scheme by RECIST 1.1 are presented below.

Table JPCE.10.2. PFS Event/Censoring Scheme by RECIST 1.1

Situation[a]	Event/Censor	Date of Event or Censor
Tumor progression or death	Event	Earliest date of PD or death
No tumor progression and no death	Censored	Date of last adequate radiological assessment or date of first treatment (whichever is later)[b]
Unless		
No baseline radiological tumor assessment available	Censored	Date of first treatment
No adequate postbaseline radiological tumor assessment available and death reported after 2 scan intervals following first treatment[b,c]	Censored	Date of first treatment
New anticancer treatment started and no tumor progression or death within 14 days	Censored	Date of last adequate radiological assessment prior to (start of new therapy + 14 days) or date of first treatment (whichever is later)[b]
Patient lost to follow-up (or withdrew consent from study participation) before documented progression or death	Censored	Date of last adequate radiological assessment or date of first treatment (whichever is later)[b]
Tumor progression or death documented immediately after 2 or more scan intervals following last adequate radiological tumor assessment or first treatment (whichever is later)[b,c]	Censored	Date of last adequate radiological assessment or date of first treatment (whichever is later)[b]

Note: Progression-free survival and associated outcome are determined by the earliest of the dates above, if more than 1 situation applies.

Abbreviations: CR = complete response; PD = progressive disease; PFS = progression-free survival; PR = partial response; RECIST 1.1 = Response Evaluation Criteria in Solid Tumors Version 1.1; SD = stable disease.

a Symptomatic deterioration (that is, symptomatic progression that is not radiologically confirmed) will not be considered as tumor progression.

b Adequate radiological tumor assessment refers to an assessment with one of the following responses: CR, PR, SD, or PD.

c Refer to the statistical analysis plan for the definition of 2 scan intervals, including any adjustment for scan window.

10.3.3. Primary Outcome and Methodology (Safety and Tolerability)

The primary objective is to evaluate safety and tolerability of abemaciclib in combination with pembrolizumab. All patients who receive at least 1 dose of study treatment will be evaluated for safety and toxicity. Adverse event terms and severity grades will be assigned by the investigator using CTCAE, Version 4.0.

Safety analyses will include summaries of the following:

- The CTCAE Version 4.0 term reported by the investigator will be mapped to the Medical Dictionary for Regulatory Activities (MedDRA) Preferred Term (PT) and System Organ

Class (SOC) using the corresponding MedDRA Lower Level Term (LLT), unless the reported CTCAE term is 'Other – specify.'
- If the reported CTCAE term is 'Other – specify,' the MedDRA LLT, PT, and SOC centrally mapped from the verbatim AE term will be used.
- All listings and summaries will use the PT resulting from this process.

Preexisting conditions are defined as AEs that begin prior to the first dose of study drug.

A treatment-emergent adverse event (TEAE) is defined as an event that first occurred or worsened in severity after baseline. Comparisons of preexisting conditions to on-treatment events at the LLT level will be used in the treatment-emergent computation. The following summaries will be produced by PT within SOC: preexisting conditions, SAEs, TEAEs, drug-related TEAEs, and procedure-related TEAEs.

The following summaries will be produced by PT within SOC and maximum CTCAE grade: laboratory-based TEAEs, nonlaboratory-based TEAEs, drug-related laboratory-based TEAEs, and drug-related nonlaboratory-based TEAEs.

Reasons for death will be summarized separately for on-therapy and within 30 days of treatment discontinuation.

Hospitalizations and transfusions during the study treatment period or during the 30-day short-term follow-up period will be summarized by cohort.

10.3.4. Other Analyses

10.3.4.1. Disposition

A detailed description of patient disposition will be provided. It will include a summary of the number patients treated, as well as number and percentage of patients completing the study or discontinued (overall and by reason for discontinuation) treated patients. All patients received treatment will be accounted for in the summation. In addition, the number of patients who are screen failures and the reason for screen failure will be summarized.

A summary of all important protocol deviations will be provided.

10.3.4.2. Patient Characteristics

Patient characteristics at baseline will be summarized by cohort:

- Patient demographics
- Baseline disease characteristics
- Historical illnesses
- Prior anticancer therapy

Other patient characteristics will be summarized as deemed appropriate

10.3.4.3. Concomitant Therapy

Concomitant medications will be summarized for the safety population.

10.3.4.4. Treatment Compliance

The number of dose omissions, reductions, and delays, cycles received, and dose intensity will be summarized for all treated patients per treatment arm. Treatment compliance information for study drug will be collected through solid oral dosage unit counts at each tumor assessment visit. The estimate of percent compliance will be given by:

Percent Compliance =x 100

The actual cumulative dose taken will be determined based on counting the number of solid oral dosage units returned at each visit and subtracting that number from the number of dosage units dispensed. The expected cumulative dose to be taken will be determined based on the assigned dose and taking into account any dose reductions or omissions.

10.3.4.5. Pharmacokinetics

Abemaciclib PK analyses will be conducted on all patients who have received at least 1 dose of study treatment and have had samples collected (see PK sampling schedule in Appendix 4).

Mean population PK parameters for abemaciclib in plasma and inter-individual PK variability will be computed using nonlinear mixed-effect modeling implemented in NONMEM. Likewise, if warranted by the data, mean population PK parameters for pembrolizumab in serum will be computed using nonlinear mixed-effect modelling.

Pharmacodynamic data (such as response rate, change in tumor size, PFS, OS, neutrophils, or diarrhea) collected in this study may be analyzed by means of NONMEM and connected to the population PK model for abemaciclib and/or pembrolizumab in a PK/pharmacodynamic model.

10.3.4.6. Biomarkers

Biomarker analyses will be based on the subset of patients from the above cohorts from whom a valid assay result (according to laboratory guideline) has been obtained. Correlative analyses will be performed to investigate associations between biomarkers and clinical endpoints as deemed appropriate.

An exploratory objective of this study is to evaluate biomarkers related to mechanism of action of abemaciclib and/or pembrolizumab, pathogenesis of cancer, immune modulation, and associations with clinical and/or safety outcomes. A corresponding exploratory objective for paired biopsies is to assess the change in expression from baseline for the biomarkers of interest to evaluate target engagement. From a statistical perspective, assuming that the standard deviation of the paired expression changes is roughly twice as large as its average, a sample size of 25 paired samples would provide ~ 70% power to detect a clinically meaningful reduction or increase in expression (say 30%) at a significance level of 5%. However, the power would be ~55% or lower if the number of paired samples available is 20 or fewer.

10.3.4.7. Health Outcomes

Change from baseline in MDASI scores will be listed and summarized for each study part at each postbaseline time point specified in the study schedule. Resource utilization will be described as appropriate.

10.3.4.7.1. Health Outcomes and Economics

For the MDASI* questionnaire (that is, the 19 core MDASI items with 2 supplemental items: one for cough and one for diarrhea) that is used in this study to collect patient self-reported information on disease and treatment-associated symptoms, along with consequent or coincident interference with affective dimensions (that is, mood, relations with others, and enjoyment with life) and activity dimensions (that is, walking, general activity, and work), the compliance rate by treatment arm will be calculated as the number of completed assessments divided by the number of expected assessments (that is, patients still on study). Compliance rates, reasons for noncompliance, and data collected for each instrument will be summarized by treatment arm.

The MDASI core (MDASI; Cleeland et al. 2000) plus 2 added items (that is, the MDASI*) single-item scores and changes from baseline will be listed. Changes from baseline will be summarized at each postbaseline time point specified in the study schedule. The summary at each post baseline time point will include mean, median, and standard deviation.

Resource use data will be summarized with descriptive statistics. If modeling is indicated, the appropriate methods will be used for the level of data measurement captured (for example: count data to be modeled with Poisson or negative binomial regression).

10.3.5. Subgroup Analyses

Subgroup analyses of interest will be further defined in the SAP.

10.3.6. Interim Analyses

An interim analysis of safety and preliminary efficacy may occur for each cohort after all patients in the given cohort have completed (or discontinued from) approximately 24 weeks of treatment. Any cohort-specific interim analyses may be combined if they are expected to occur within approximately a month, and interim analyses may also be combined with the ongoing trial-level safety review or annual safety review for annual safety update reporting.

11. References

Bremens RM, Al-Shibili K, Donnem T, Sirera R, Al-Saad S, Andersen S, Stenvold H, Camps C, Busund LT. The role of tumor-infiltrating immune cells and chronic inflammation at the tumor site on cancer development, progression, and prognosis: emphasis on non-small cell lung cancer. *J Thor Onc*. 2011;6(4):824-833.

Cleeland CS, Mendoza TR, Wang XS, Chou C, Harle MT, Morrissey M, Engstrom MC. Assessing symptom distress in cancer patients: the M.D. Anderson Symptom Inventory. *Cancer.* 2000;89(7):1634–1646.

Cockcroft DW, Gault MD. Prediction of creatinine clearance from serum creatinine. *Nephron*. 1976;16:31-41.

Disis ML. Immune regulation of cancer. *J Clin Oncol.* 2010;28(29):4531-4538.

Eisenhauer EA, Therasse P, Bogaert J, Schwartz LH, Sargent D, Ford R, Dancey J, Arbuck S, Gwyther S, Mooney M, Rubinstein L, Shankar L, Dodd L, Kaplan R, Lacombe D, Verweij J. New response evaluation criteria in solid tumours: revised RECIST guideline (version 1.1). *Eur J Cancer*. 2009;45(2) 228-247.

Gangadhar TC, Mehnert J, Patnaik A, Hamid O, Carlino MS, Hodi FS, Blank CU, Ribas A, Robert C, Kondic AG, Ahamadi M, Freshwater T, de Greef R, van Vugt M, Lubiniecki G, Ebbinghaus S, Kang SP, Daud A. Population pharmacokinetic (popPK) model of pembrolizumab (pembro; MK-3475) in patients (pts) treated in KEYNOTE-001 and KEYNOTE-002. *J Clin Onc.* 2015;33(15):3058.

Hammond ME, Hayes DF, Dowsett M, Allred DC, Hagerty KL, Badve S, Fitzgibbons PL, Francis G, Goldstein NS, Hayes M, Hicks DG, Lester S, Love R, Mangu PB, McShane L, Miller K, Osborne CK, Paik S, Perlmutter J, Rhodes A, Sasano H, Schwartz JN, Sweep FC, Taube S, Torlakovic EE, Valenstein P, Viale G, Visscher D, Wheeler T, Williams RB, Wittliff JL, Wolff AC. American Society of Clinical Oncology/College of American Pathologists guideline recommendations for immunohistochemical testing of estrogen and progesterone receptors in breast cancer. *J Clin Oncol*. 2010;28:2784.

Herbst RS, Baas P, Kim DW, Felip E, Perez-Gracia JL, Han JY, Molina J, Kim JH, Arvis CD, Ahn MJ, Majem M, Fidler MJ, de Castro G, Garrido M, Lubiniecki GM, Shentu Y, Im E, Dolled-Filhart M, Garon EB. Pembrolizumab versus docetaxel for previously treated, PD-L1-positive, advanced non-small-cell lung cancer (KEYNOTE-010): a randomised controlled trial. *Lancet.* 2015 published online Dec 19. http://dx.doi.org/10.1016/S0140-6736(15)01281-7.

Kaplan EL, Meier P. Nonparametric estimation of incomplete observations. *J Amer Stat Assoc*. 1958;53:457-481.

Liu F, Lang R, Zhao J, Zhang X, Pringle GA, Fan Y, Yin D, Gu F, Yao Z, Fu L. $CD8^+$ cytotoxic T cell and $FOXP3^+$ regulatory T cell infiltration in relation to breast cancer survival and molecular subtypes. *Breast Can Res Treat*. 2011;130(2):645-655.

Mei Z, Liu Y, Liu C, Cui A, Liang Z, Wang G, Peng H, Cui L, Li C. Tumour-infiltrating inflammation and prognosis in colorectal cancer: systematic review and meta-analysis. *Br J Cancer.* 2014;110:1595-1605.

[NCI] National Cancer Institute. Cancer Therapy Evaluation Program, Common Terminology Criteria for Adverse Events, Version 4.0, DCTD, NCI, NIH, DHHS. 2009. Publish date: 29 May 2009.

Oken MM, Creech RH, Tormey DC, Horton J, Davis TE, McFadden ET, Carbone PP. Toxicity and response criteria of the eastern cooperative oncology group. *Am J Clin Oncol*. 1982;5:649-655.

Patnaik A, Rosen LS, Tolaney SM, Tolcher AW, Goldman JW, Gandhi L, Papadopoulos KP, Beeram M, Rasco DW, Myrand SP, Kulanthaivel P, Andrews JM, Frenzel M, Cronier D, Chan EM, Flaherty K, Wen PY, Shapiro G. LY2835219, a novel cell cycle inhibitor selective for CDK4/6, in combination with fulvestrant for patients with hormone receptor positive (HR+) metastatic breast cancer. *J Clin Oncol*. 2014;32(suppl):5s. Abstract 534.

Puyol M, Martin A, Dubus P, Mulero F, Pizcueta P, Khan G, Guerra C, Santamaría D, Barbacid M. A synthetic lethal interaction between K-Ras oncogenes and Cdk4 unveils a therapeutic strategy for non-small cell lung cancer. *Cancer Cell*. 2010;18(1):63-73.

Seymour L, Bogaerts J, Perrone A, Ford R, Schwartz LH, Mandrekar S, Lin NU, Litière S, Dancey J, Chen A, Hodi FS, Therasse P, Hoekstra OS, Shankar LK, Wolchok JD, Ballinger M, Caramella C, de Vries EG; RECIST working group. iRECIST: guidelines for response criteria for use in trials testing immunotherapeutics. *Lancet Oncol.* 2017;18(3):e143-e152.

Talmadge JE. Immune cell infiltration of primary and metastatic lesions: Mechanisms and clinical impact. *Sem Can Bio*. 2011;21(2):131-138.

Tate SC, Cai S, Ajamie RT, Burke T, Beckmann RP, Chan EM, De Dios A, Wishart GN, Gelbert LM, Cronier DM. Semi-Mechanistic Pharmacokinetic/Pharmacodynamic Modeling of the Antitumor Activity of LY2835219, a New Cyclin-Dependent Kinase 4/6 Inhibitor, in Mice Bearing Human Tumor Xenografts. *Clin Cancer Res.* 2014;20:3763.

Wolff AC, Hammond ME, Hicks DG, Dowsett M, McShane LM, Allison KH, Allred DC, Bartlett JM, Bilous M, Fitzgibbons P, Hanna W, Jenkins RB, Mangu PB, Paik S, Perez EA, Press MF, Spears PA, Vance GH, Viale G, Hayes DF. Recommendations for human epidermal growth factor receptor 2 testing in breast cancer: American Society of Clinical Oncology/College of American Pathologists clinical practice guideline update. *J Clin Oncol.* 2013;31(31):3997-4013.

Appendix 1. Abbreviations and Definitions

Term	Definition
**ADA**	anti-drug antibody
**AE**	Adverse event: any untoward medical occurrence in a patient or clinical investigation patient administered a pharmaceutical product and that does not necessarily have a causal relationship with this treatment. An adverse event can therefore be any unfavorable and unintended sign (including an abnormal laboratory finding), symptom, or disease temporally associated with the use of a medicinal (investigational) product, whether or not related to the medicinal (investigational) product.
**ALT**	alanine aminotransferase
**AST**	aspartate aminotransferase
**CIOMS**	Council for International Organizations of Medical Sciences
**collection database**	a computer database where clinical trial data are entered and validated.
**CRP**	Clinical research physician: Individual responsible for the medical conduct of the study. Responsibilities of the CRP may be performed by a physician, clinical research scientist, global safety physician, or other medical officer.
**CTCAE**	Common Terminology Criteria for Adverse Events
**CYP**	cytochrome P450
**DCR**	disease control rate
**DoR**	duration of response
**ECG**	Electrocardiogram
**ECI**	Events of Clinical Interest
**ECOG**	Eastern Cooperative Oncology Group
**eCRF**	electronic case report form
**effective method of contraception**	Effective method of contraception means male condom with spermicide, female condom with spermicide, diaphragm with spermicide, cervical sponge, or cervical cap with spermicide.<br>Also see the definition of highly effective method of contraception.
**enroll**	The act of assigning a patient to a treatment. Patients who are enrolled in the trial are those who have been assigned to a treatment.

Term	Definition
**enter**	Patients entered into a trial are those who sign the informed consent form directly or through their legally acceptable representatives.
**ERB**	ethical review board
**GCP**	good clinical practice
**HER2**	human epidermal growth factor receptor 2
**highly effective method of contraception**	combined oral contraceptive pill and mini-pill, NuvaRing®, implantable contraceptives, injectable contraceptives (such as Depo-Provera®), intrauterine device (such as Mirena® and ParaGard®), contraceptive patch for women <90 Kg (<198 pounds), total abstinence, or vasectomy.<br>Also see the definition of effective method of contraception.
**H&P**	history and physical
**HR+**	hormone receptor positive
**IB**	investigator's brochure
**ICF**	informed consent form
**ICH**	International Council for Harmonisation
**ILD**	interstitial lung disease
**interim analysis**	An interim analysis is an analysis of clinical trial data conducted before the final reporting database is created/locked.
**investigational product**	A pharmaceutical form of an active ingredient or placebo being tested or used as a reference in a clinical trial, including products already on the market when used or assembled (formulated or packaged) in a way different from the authorized form, or marketed products used for an unauthorized indication, or marketed products used to gain further information about the authorized form.
**IRB**	institutional review board
**irRECIST**	immune-related Response Evaluation Criteria in Solid Tumors
KRAS mt	Kirsten rat sarcoma mutant
**MATE**	multidrug and toxin extrusion protein
**MDASI**	MD Anderson Symptom Inventory
**MedDRA**	Medical Dictionary for Regulatory Activities
**NSCLC**	non-small cell lung cancer
**ORR**	objective response rate

Term	Definition
**OS**	overall survival
**PD-L1**	programmed death ligand 1
**PFS**	progression-free survival
**PK**	pharmacokinetic(s)
**QTc**	corrected QT interval
**RECIST**	Response Evaluation Criteria in Solid Tumors
**SAE**	serious adverse event
**SAP**	Statistical Analysis Plan
**screen**	The act of determining if an individual meets minimum requirements to become part of a pool of potential candidates for participation in a clinical study.
**screen failure**	patient who does not meet one or more criteria required for participation in a trial
**SUSAR**	suspected unexpected serious adverse reaction
**TBL**	total bilirubin
**TEAE**	Treatment-emergent adverse event: an untoward medical occurrence that emerges during a defined treatment period, having been absent pretreatment, or worsens relative to the pretreatment state, and does not necessarily have to have a causal relationship with this treatment.
**ULN**	upper limit of normal
**VTE**	venous thromboembolic event

Appendix 2. Study Governance, Regulatory, and Ethical Considerations

Informed Consent

The investigator is responsible for:

- ensuring that the patient understands the potential risks and benefits of participating in the study
- ensuring that informed consent is given by each patient. This includes obtaining the appropriate signatures and dates on the ICF prior to the performance of any study procedures and prior to the administration of study treatment.
- answering any questions the patient may have throughout the study and sharing in a timely manner any new information that may be relevant to the patient's willingness to continue his or her participation in the trial.

Ethical Review

Documentation of ERB/IRB approval of the protocol and the ICF must be provided to Lilly before the study may begin at the investigative sites. Lilly or its representatives must approve the ICF, including any changes made by the ERBs/IRBs, before it is used at the investigative sites. All ICFs must be compliant with the ICH guideline on GCP.

The study site's ERBs/IRBs should be provided with the following:

- the current IB or package labeling, Patient Information Leaflet, Package Insert, or Summary of Product Characteristics and updates during the course of the study
- the ICF
- relevant curricula vitae

Regulatory Considerations

This study will be conducted in accordance with:

- consensus ethics principles derived from international ethics guidelines, including the Declaration of Helsinki and Council for International Organizations of Medical Sciences (CIOMS) International Ethical Guidelines
- applicable ICH GCP Guidelines
- applicable laws and regulations.

Some obligations of Lilly may be assigned to a third-party organization.

Investigator Information

Licensed physicians specializing in oncology will participate as investigators in this clinical trial.

Protocol Signatures

Lilly's responsible medical officer will approve the protocol, confirming that, to the best of his or her knowledge, the protocol accurately describes the planned design and conduct of the study.

After reading the protocol, each principal investigator will sign the protocol signature page and send a copy of the signed page to a Lilly representative.

Final Report Signature

The investigator will sign the final clinical study report for this study, indicating agreement, to the best of his or her knowledge, with the analyses, results, and conclusions of the report.

The clinical study report coordinating investigator will sign the final clinical study report for this study, indicating agreement that, to the best of his or her knowledge, the report accurately describes the conduct and results of the study.

An investigator will be chosen by Lilly to serve as the clinical study report coordinating investigator. If this investigator is unable to fulfill this function, another investigator will be chosen by Lilly to serve as the clinical study report coordinating investigator.

Lilly's responsible medical officer and statistician will approve the final clinical study report for this study, confirming that, to the best of his or her knowledge, the report accurately describes the conduct and results of the study.

Data Quality Assurance

To ensure accurate, complete, and reliable data, Lilly or its representatives will do the following:

- provide instructional material to the study sites, as appropriate
- sponsor start-up training to instruct the investigators and study coordinators. This session will give instruction on the protocol, the completion of the CRFs, and study procedures.
- make periodic visits to the study site
- be available for consultation and stay in contact with the study site personnel by mail, telephone, and/or fax
- review and evaluate CRF data and use standard computer edits to detect errors in data collection
- conduct a quality review of the database

In addition, Lilly or its representatives will periodically check a sample of the patient data recorded against source documents at the study site. The study may be audited by Lilly or its representatives, and/or regulatory agencies at any time. Investigators will be given notice before an audit occurs.

The investigator will keep records of all original source data. This might include laboratory tests, medical records, and clinical notes. If requested, the investigator will provide Lilly, applicable regulatory agencies, and applicable ERBs/IRBs with direct access to original source documents.

Data Capture System

An electronic data capture system will be used in this study. The site maintains a separate source for the data entered by the site into Lilly-provided electronic data capture system.

Case report form data will be encoded and stored in a clinical trial database. Data managed by a central vendor, such as laboratory test data, will be stored electronically in the central vendor's database system. Data will subsequently be transferred from the central vendor to the Lilly data warehouse.

Any data for which the paper documentation provided by the patient will serve as the source document will be identified and documented by each site in that site's study file. Paper documentation provided by the patient may include, for example, a paper diary to collect patient-reported outcome (PRO) measures (for example, MDASI) or a participant diary.

Data from complaint forms submitted to Lilly will be encoded and stored in the global product complaint management system.

Study and Site Closure

Discontinuation of Study Sites

Study site participation may be discontinued if Lilly or its designee, the investigator, or the ERB/IRB of the study site judges it necessary for medical, safety, regulatory, or other reasons consistent with applicable laws, regulations, and GCP.

Discontinuation of the Study

The study will be discontinued if Lilly judges it necessary for medical, safety, regulatory, or other reasons consistent with applicable laws, regulations, and GCP.

Appendix 3. Clinical Laboratory Tests

Clinical Laboratory Tests

**Hematology** - central laboratory	
Leukocytes (WBC)	Erythrocytes (RBC)
Neutrophils[a]	Hemoglobin (HGB)
Lymphocytes	Hematocrit (HCT)
Monocytes	Mean corpuscular volume (MCV)
Eosinophils	Mean corpuscular hemoglobin concentration (MCHC)
Basophils	Platelets (PLT)
**Coagulation** - local laboratory	
Activated partial thromboplastin time (aPTT)	
Prothrombin Intl. normalized ratio (INR) or Prothrombin time (PT)	
**Clinical Chemistry** - central laboratory (except as indicated)[c]	
**Serum Concentrations of:**	
Alanine aminotransferase (ALT)	Calcium
Albumin	Chloride
Alkaline phosphatase	Creatinine[b]
Aspartate aminotransferase (AST)	Cystatin C
Bilirubin, direct	Potassium
Bilirubin, total	Protein, total
Blood urea nitrogen (BUN) or blood urea	Sodium
**Pregnancy Test** (for female patients of childbearing potential)[c]	
Serum pregnancy test	
Urine pregnancy test	
**Other Laboratory Tests** – central laboratory	
Free thyroxine (FT4)	
Thyroid stimulating hormone (TSH)	
Triiodothyronine (T3) or Free Triiodothyronine (FT3)	

Abbreviations: Intl = international; RBC = red blood cells; WBC = white blood cells.

[a] Neutrophils reported by automated differential hematology instruments include both segmented and band forms. When a manual differential is needed to report the neutrophils, the segmented and band forms should be added together and recorded on the CRF, unless the CRF specifically provides an entry field for bands.

[b] Creatinine clearance (CrCl) requires the use of local creatinine result. Creatinine will be assayed by both central and local laboratories when used to calculate CrCl, otherwise only to be drawn centrally.

[c] Local or investigator-designated laboratory.

Appendix 4. Sampling Schedule for Biomarkers/ Pharmacokinetics/ Pharmacodynamics

It is essential that the exact infusion start and stop times (actual clock readings), as well as infusion parameters (such as, type of infusion pump, flow rate settings) are recorded. The exact time of collection of each venous blood sample will be based on the clock used to record infusion times. It is essential that the pharmacokinetic blood samples not be withdrawn from the same site as the drug infusion.

Pharmacokinetic sampling windows are provided as guidance. Due to practical and logistical concerns, some deviation from the specified sampling time is normal and expected. Sites should keep in mind that drawing the sample and recording the actual time on the appropriate form is of primary importance. Differences from the time specified in the protocol are not considered protocol deviations as long as samples are collected and accurate dates and times are recorded in a timely manner on the appropriate forms.

Sampling Schedule for Biomarkers/ Pharmacokinetics/Pharmacodynamics

Procedure	Baseline/Cycle 1	Cycle 2	Cycle 3	Cycle 6	Cycle 8	Short-Term Follow-Up
Pharmacokinetics of Abemaciclib and Pembrolizumab[a]	C1D1: Before infusion<br>C1D8: anytime during visit	C2D1: Before infusion	C3D1: Before infusion	C6D1: Before infusion	C8D1: Before infusion	
Tumor tissue	Baseline: Obtain biopsy specimen (either newly collected within 28 days of C1D1 **OR** if cut slides are from an archive specimen, they must be submitted to the testing laboratory within 14 days and 28 days prior to C1D1)		Tumor collection C3D1 (+7 days)			Optional tumor tissue may be requested at the time of progressive disease. This is not a mandatory collection
Plasma	C1D1: Before infusion	C2D1: Before infusion				Any time during the short-term follow-up visit prior to beginning new postdiscontinuation anticancer therapy
Whole blood (flow)	C1D1: Before infusion<br>C1D1: 8 hours post initiating infusion	C2D1: Before infusion	C3D1: Before infusion			
Whole blood (PaxGene)	C1D1: Before infusion	C2D1: Before infusion	C3D1: Before infusion			
Whole blood	C1D1: Before infusion	C2D1: Before infusion		C6D1: Before infusion		
Serum	C1D1: Before infusion<br>C1D1: 8 hours post initiating infusion	C2D1: Before infusion	C3D1: Before infusion			
Whole blood (PGx)	any time prior to the first infusion[b]					

Abbreviations: C = cycle; D = day; IRR = infusion-related reaction.

[a] For pembrolizumab: In the event of an IRR, additional blood samples will be collected for pharmacokinetics analysis at the following time points: (i) as close as possible to the onset of the IRR, (ii) at the resolution of the IRR, and (iii) 30 days following the IRR.

[b] A pretreatment blood sample is preferred; however, the whole blood sample for genetic analysis may be collected at a later time point if necessary.

Appendix 5. Creatinine Clearance Formula

Note: This formula is to be used for calculating creatinine clearance (CrCl) from **local laboratory results only.**

For serum creatinine concentration in mg/dL:

$$\textbf{CrCl} = \frac{(\mathbf{140 - age^{a}}) \times (\mathbf{wt}) \times \mathbf{0.85} \text{ (if female), or } \times \mathbf{1.0} \text{ (if male)}}{\mathbf{72} \times \textbf{serum creatinine } (mg/dL)}$$

(mL/min)

For serum creatinine concentration in µmol/L:

$$\textbf{CrCl} = \frac{(\mathbf{140 - age^{a}}) \times (\mathbf{wt}) \times \mathbf{0.85} \text{ (if female), or } \times \mathbf{1.0} \text{ (if male)}}{\mathbf{0.81} \times \textbf{serum creatinine } (\mu mol/L)}$$

(mL/min)

[a] Age in years, weight (wt) in kilograms.
Source: Cockcroft and Gault 1976.

Appendix 6. Protocol JPCE Inducers and Strong Inhibitors of CYP3A

The information in this table is provided for guidance to investigators and does not preclude the use of these medications if clinically indicated.

**Strong Inducers of CYP3A**
Carbamazepine
Dexamethasone[a]
Phenobarbital/phenobarbitone
Phenytoin
Rifapentine
Rifampin
Rifabutin
St John's wort

**Moderate Inducers of CYP3A**
Bosentan
Lesinurad
Modafinil
Primidone
Telotristat ethyl

**Strong Inhibitors of CYP3A**
Aprepitant
Ciprofloxacin
Clarithromycin
Conivaptan
Diltiazem
Erythromycin
Fluconazole
Itraconazole
Ketoconazole
Nefazodone
Posaconazole
Troleandomycin
Verapamil

[a] Important note: All patients may receive supportive therapy with dexamethasone, preferably for ≤7 days, if clinically indicated.

Appendix 7. RECIST 1.1 and irRECIST Criteria

Response and progression will be evaluated in this study using the international criteria proposed by the New Response Evaluation Criteria in Solid Tumors (RECIST): Revised RECIST Guideline (version 1.1; Eisenhauer et al. 2009) (as a secondary objective) and by the immune-related Response Evaluation Criteria in Solid Tumors (irRECIST) (as an exploratory objective only).

RECIST 1.1

Measurability of Tumor at Baseline

Tumor lesions/lymph nodes will be categorized at baseline as measurable or nonmeasurable. Measurable disease is defined by the presence of at least 1 measurable lesion.

Measurable
Tumor lesions: Measured in at least 1 dimension (longest diameter in the plane of measurement is to be recorded) with a minimum size of:

- 10 mm by computed tomography (CT) or magnetic resonance imaging (MRI) scan (slice thickness ≤5 mm)
- 10 mm caliper measurement by clinical exam (nonmeasurable lesions if cannot be accurately measured with calipers)
- 20 mm by chest X-ray.

Malignant lymph nodes: To be considered pathologically enlarged and measurable, a lymph node must be ≥15 mm in short axis when assessed by CT scan (CT scan thickness recommended to be ≤5 mm).

Nonmeasurable
All other lesions, including small lesions (longest diameter <10 mm or pathological lymph nodes with ≥10 to <15 mm short axis) as well as truly nonmeasurable lesions. Lesions considered truly nonmeasurable include: leptomeningeal disease, ascites, pleural/pericardial effusions, lymphangitis cutis/pulmonis, inflammatory breast disease, lymphangitis involvement of skin or lung, abdominal masses/abdominal organomegaly identified by physical exam that is not measurable by reproducible imaging techniques.

Special Considerations for Lesion Measurability

Bone Lesions:

- Bone scan, positron emission tomography (PET) scan or plain films are not considered adequate imaging techniques to measure bone lesions.

- Lytic bone lesions or mixed lytic-blastic lesions, with identifiable soft tissue components, that can be evaluated by cross sectional imaging techniques such as CT or MRI, can be considered measurable lesions if the soft tissue component meets the definition of measurability.
- Blastic bone lesions are nonmeasurable.

Cystic Lesions:

- Simple cysts should not be considered as malignant lesions (neither measurable nor nonmeasurable)
- Cystic lesions thought to represent cystic metastases can be considered as measurable lesions, if they meet the definition of measurability. If noncystic lesions are presented in the same patients, these are preferred for selection as target lesions.

Lesions with Prior Local Treatment:

- Tumor lesions situated at a previously irradiated area, or in an area subjected to other loco-regional therapy, are nonmeasurable unless there has been demonstrated progression in the lesion.

Baseline Documentation of Target and Nontarget Lesion

Target Lesions

When more than 1 measurable lesion is present at baseline, all lesions up to a maximum of 5 lesions total (and a maximum of 2 lesions per organ) representative of all involved organs should be identified as target lesions and will be recorded and measured at baseline. Non-nodal Target lesions should be selected on the basis of their size (lesions with the longest diameter), be representative of all involved organs, and can be reproduced in repeated measurements. Measurable lymph nodes are target lesions if they meet the criteria of a short axis of ≥15 mm by CT scan. All measurements are to be recorded in the eCRF in millimeters (or decimal fractions of centimeters [cm]).

Nontarget Lesions

All other lesions (or sites of disease) are identified as nontarget lesions (chosen based on their representativeness of involved organs and the ability to be reproduced in repeated measurements) and should be recorded at baseline. Measurement of these lesions are not required but should be followed as ‘present,’ ‘absent,’ or in rare cases ‘unequivocal progression.’ In addition, it is possible to record multiple nontarget lesions involving the same organ as a single item on the eCRF (for example, multiple liver metastases recorded as 1 liver lesion).

Lymph nodes with short axis ≥10 mm but <15 mm should be considered nontarget lesions. Nodes that have a short axis <10 mm are considered nonpathological and are not recorded or followed.

Specifications by Methods of Measurement

All measurements should be recorded in metric notation, using a ruler or calipers if clinically assessed. All baseline evaluations should be performed as closely as possible to the beginning of treatment and never more than 4 weeks before the beginning of the treatment.

The same method of assessment and the same technique should be used to characterize each identified and reported lesion at baseline and during follow up. Imaging-based evaluation is should always be done rather than clinical examination unless the lesion(s) being followed cannot be imaged but are assessed by clinical exam.

An adequate volume of a suitable contrast agent should be given so that the metastases are demonstrated to best effect and a consistent method is used on subsequent examinations for any given patient. If prior to enrollment it is known a patient is not able to undergo CT scans with intravenous contrast due to allergy or renal insufficiency, the decision as to whether a noncontrast CT or MRI (with or without intravenous contrast) should be used to evaluate the patient at baseline and follow up should be guided by the tumor type under investigation and the anatomic location of the disease.

Clinical Lesions: Clinical lesions will only be considered measurable when they are superficial and ≥10 mm diameter as assessed using calipers (for example, skin nodules). For the case of skin lesions, documentation by color photography, including a ruler to estimate the size of the lesion is recommended. When lesions can be evaluated by both clinical exam and imaging, imaging evaluation should be undertaken since it is more objective and may be reviewed at the end of the study.

Chest X-ray: Chest CT is preferred over chest X-ray when progression is an important endpoint. Lesions on chest X-ray may be considered measurable if they are clearly defined and surrounded by aerated lung.

CT and MRI: CT scan is the best currently available and reproducible method to measure lesions selected for response assessment. Measurability of lesions on CT scan is based on the assumption that CT slice thickness is ≤5 mm. When CT scan have slice thickness >5 mm, the minimum size for a measurable lesion should be twice the slice thickness. MRI is also acceptable in certain situations (for example, for body scans). If there is concern about radiation exposure at CT, MRI may be used instead of CT in selected instances.

Ultrasound: Ultrasound should not be used to measure lesion size. Ultrasound examinations cannot be reproduced in their entirety for independent review at a later date and, because they are operator dependent, it cannot be guaranteed that the same technique and measurements will be taken from one assessment to the next. If new lesions are identified by ultrasound in the course of the study, confirmation by CT or MRI is advised.

Endoscopy, Laparoscopy: The utilization of these techniques for objective tumor evaluation is not advised. However, such techniques can be useful to confirm complete pathological response when biopsies are obtained or to determine relapse in trials where recurrence following complete response (CR) or surgical resection is an endpoint.

Tumor Markers: Tumor markers alone cannot be used to assess tumor response. If markers are initially above the upper normal limit, they must normalize for a patient to be considered in CR. Specific guidelines for both prostate-specific antigen (PSA) response (in recurrent prostate cancer) and CA-125 response (in recurrent ovarian cancer) have been published.

Cytology, Histology: These techniques can be used to differentiate between partial responses (PR) and CR in rare cases if required by protocol (for example, residual lesions in tumor types such as germ cell tumors, where known residual benign tumors can remain). When effusions are known to be a potential adverse effect of treatment (for example, with certain taxane compounds or angiogenesis inhibitors), the cytological confirmation of the neoplastic origin of any effusion that appears or worsens during treatment can be considered if the measurable tumor has met criteria for response or stable disease (SD) in order to differentiate between response (or SD) and progressive disease.

Pet Scan (FDG-PET, PET CT): PET is not recommended for lesion assessment. If a new lesion is found by PET, another assessment must be done by CT, unless the PET CT is of diagnostic quality. If CT is done to confirm the results of the earlier PET scan, the date of progression must be reported as the earlier date of the PET scan.

Bone Scan: If lesions measured by bone scan are reported at baseline, it is necessary to repeat the bone scan when trying to identify a CR or PR in target disease or when progression in bone is suspected.

Response Criteria

Evaluation of Target Lesions

Complete Response (CR): Disappearance of all target lesions. Any pathological lymph nodes (whether target or nontarget) must have reduction in short axis to <10 mm. Tumor marker results must have normalized.

Partial Response (PR): At least a 30% decrease in the sum of diameter of target lesions, taking as reference the baseline sum diameters.

Progressive Disease: At least a 20% increase in the sum of the diameters of target lesions, taking as reference the smallest sum on study (including the baseline sum if that is the smallest). In addition to the relative increase of 20%, the sum must also demonstrate an absolute increase of at least 5 mm. The appearance of one or more new lesions is also considered progression.

For equivocal findings of progression (for example, very small and uncertain new lesions; cystic changes or necrosis in existing lesions), treatment may continue until the next scheduled assessment. If at the next scheduled assessment, progression is confirmed, the date of progression should be the earlier date when progression was suspected.

Stable Disease (SD): Neither sufficient shrinkage to qualify for PR nor sufficient increase to qualify for progressive disease, taking as reference the smallest sum diameters while on study.

Not Evaluable: When an incomplete radiologic assessment of target lesions is performed or there is a change in the method of measurement from baseline that impacts the ability to make a reliable evaluation of response.

Evaluation of Nontarget Lesions

Complete Response: Disappearance of all nontarget lesions and normalization of tumor marker level. All lymph nodes must be nonpathological or normal in size (<10 mm short axis).

Non-CR/ non-progressive disease: Persistence of 1 or more nontarget lesions and/or maintenance of tumor marker level above the normal limits.

Progressive Disease: Unequivocal progression of existing nontarget lesions. The appearance of 1 or more new lesions is also considered progression.

Not Evaluable: When a change in method of measurement from baseline occurs and impacts the ability to make a reliable evaluation of response.

Evaluation of Best Overall Response

The best overall response is the best response recorded from the start of the study treatment until the earliest of objective progression or start of new anticancer therapy, taking into account any requirement for confirmation. The patient's best overall response assignment will depend on the findings of both target and nontarget disease and will also take into consideration the appearance of new lesions. The best overall response will be calculated via an algorithm using the assessment responses provided by the investigator over the course of the trial.

Time Point Response

It is assumed that at each protocol-specified time point, a response assessment occurs. (When no imaging/measurement is done at all at a particular time point, the patient is not evaluable (NE) at that time point.) Table 1 provides a summary of the overall response status calculation at each time point for patients who have *measurable disease* at baseline.

Table 1. Time Point Response: Patients with Target (± Nontarget) Disease

Target Lesions	Nontarget Lesions	New Lesions	Overall Response
CR	CR	No	CR
CR	Non-CR/non-progressive disease	No	PR
CR	Not evaluated	No	PR
PR	Non-progressive disease or not all evaluated	No	PR
SD	Non-progressive disease or not all evaluated	No	SD
Not all evaluated	Non-progressive disease	No	NE
Progressive disease	Any	Yes or No	Progressive disease
Any	Progressive disease	Yes or No	Progressive disease
Any	Any	Yes	Progressive disease

Abbreviations: CR = complete response; PR = partial response; SD = stable disease; NE = inevaluable.

Table 2 is to be used when patients have *nonmeasurable* disease only.

Table 2. Time Point Response: Patients with Nontarget Disease Only

Nontarget Lesions	New Lesions	Overall Response
CR	No	CR
Non-CR/non-progressive disease	No	Non-CR/non-progressive disease[a]
Not all evaluated	No	NE
Unequivocal progressive disease	Yes or No	Progressive disease
Any	Yes	Progressive disease

Abbreviations: CR = complete response; NE = inevaluable.

[a] non-CR/non-progressive disease is preferred over SD for nontarget disease.

Frequency of Tumor Re-Evaluation

A baseline tumor evaluation must be performed within 4 weeks before patient begins study treatment. Frequency of tumor re-evaluation while on and adapted to treatment should be protocol-specific and adapted to the type and schedule of treatment. In the context of Phase 2 studies where the beneficial effect therapy is not known, follow up every 6-8 weeks is reasonable. Normally, all target and nontarget sites are evaluated at each assessment using the same method. However, bone scans may need to be repeated only when CR is identified in target disease or when progression in bone is suspected.

Confirmatory Measurement/Duration of Response

Confirmation:
The main goal of confirmation of objective response in clinical trials is to avoid overestimating the response rate observed. The confirmation of response is particularly important in *nonrandomized trials* where response (CR/PR) is the primary end point. In this setting, to be assigned a status of PR/CR, changes in tumor measurements must be confirmed by repeat assessments that should be performed no less than 4 weeks after the criteria for response are first met. To confirm a response of CR, a full assessment of all target and nontarget lesions that were present at baseline must occur, including those measured by bone scan. To confirm a PR or SD, a full assessment of target lesions that were present at baseline must occur; assessment of nontargets is not required.

However, in *randomized trial* (Phase 2 or 3) or studies where SD or progression is the primary endpoint, confirmation of response is not required. But elimination of the requirement may increase the importance of central review to protect against bias, in particular of studies which are not blinded.

In the case of SD, follow-up measurements must have met the SD criteria at least once after start of treatment at a minimum interval not less than 6 weeks measured from first dose.

Duration of Overall Response
The duration of overall response is measured from the time measurement criteria are first met for CR or PR (whichever is first recorded) until the first date that disease is recurrent or objective progression is observed (taking as reference for progressive disease the smallest measurements recorded on study).

The duration of overall CR is measured from the time measurement criteria are first met for CR until the first date that recurrent disease is objectively documented.

Duration of Stable Disease
Stable disease is measured from the start of the treatment (in randomized trials, from date of randomization) until the criteria for objective progression are met, taking as reference the smallest sum on study (if the baseline sum is the smallest, that is the reference for calculation of progressive disease).

Independent Review of Response and Progression

When objective response (CR + PR) is the primary end point, and when key drug development decisions are based on the observation of a minimum number of responders, it is recommended that all claimed responses be reviewed by an expert(s) independent of the study. If the study is a randomized trial, ideally reviewers should be blinded to treatment assignment.

irRECIST Assessment of Disease
As noted above, if tumor imaging shows initial disease progression, the study site may elect to continue treatment, repeat imaging ≥ 4 weeks later and assess tumor response or confirmed progression per irRECIST.

irRECIST is RECIST 1.1 adapted as described below to account for the unique tumor response seen with immunotherapeutic drugs. irRECIST will be used by site investigator/local radiology review to assess tumor response and progression, and make treatment decisions. This data will be collected in the clinical database.

Table 3. Imaging and Treatment after First Radiologic Evidence of PD

	Clinically Stable		Clinically Unstable	
	**Imaging**	**Treatment**	**Imaging**	**Treatment**
1st radiologic evidence of PD by RECIST 1.1	Repeat imaging at ≥ 4 weeks at site to confirm PD	May continue study treatment at the local site Investigator's discretion while awaiting confirmatory tumor imaging by site by irRECIST.	Repeat imaging at ≥ 4 weeks to confirm PD per physician discretion only	Discontinue treatment
Repeat tumor imaging confirms PD by irRECIST at the local site	No additional imaging required	Discontinue treatment (exception is possible upon consultation with sponsor)	No additional imaging required	N/A
Repeat tumor imaging shows SD, PR or CR by irRECIST by the local site	Continue regularly scheduled imaging assessments	Continue study treatment at the local site Investigator's discretion	Continue regularly scheduled imaging assessments	May restart study treatment if condition has improved and/or clinically stable per Investigator's discretion. Next tumor image should occur according to the every X week (XX ± 7 days) imaging schedule for the first year and Y weeks (XX ± 7 days) after the first year

Abbreviations: CR = complete response; irRECIST = immune-related Response Evaluation Criteria in Solid Tumors; N/A = not applicable; PD = progressive disease; PR = partial response; RECIST 1.1 = Response Evaluation Criteria in Solid Tumors Version 1.1; SD = stable disease.

- In determining whether or not the tumor burden has increased or decreased, local study site investigators should consider all target lesions as well as non-target lesions. Subjects that are deemed clinically unstable are not required to have repeat tumor imaging for confirmation.

- For a **clinically stable** subject with first radiologic evidence of progressive disease by RECIST 1.1 (that is, **unconfirmed progression of disease**), it is at the discretion of the site investigator to continue treating the subject with the assigned treatment per protocol until progression of disease is confirmed at least 28 days from the date of the scan first suggesting PD. If radiologic progression is confirmed by subsequent scan then the subject will be discontinued from trial treatment. If radiologic progression is not confirmed by irRECIST per the site, then the subjects may continue on treatment and follow the regular imaging schedule intervals until progression is confirmed at a later time point by the site.

    - **NOTE**: If a subject has confirmed radiographic progression (that is, 2 scans at least 4 weeks apart demonstrating progressive disease) per irRECIST, but the subject is

achieving a clinically meaningful benefit, and there is no further increase in the tumor burden at the confirmatory tumor imaging, an exception to continue treatment may be considered following consultation with the sponsor. In this case, if treatment is continued, tumor imaging should continue to be performed following the intervals as outlined in Table 3

- Any subject deemed **clinically unstable** should be discontinued from trial treatment at 1st radiologic evidence of PD and is not required to have repeat imaging for PD confirmation.
- In subjects who discontinue study therapy without documented disease progression, every effort should be made to continue monitoring their disease status by tumor imaging using the same imaging schedule used while on treatment (every Y weeks (XX days ± 7 days), until (1) the start of new anti-cancer treatment, (2) disease progression (3) death, or (4) the end of the study, whichever occurs first.

irRECIST data will be collected in the clinical database.

Appendix 8. ECOG Performance Status

Eastern Cooperative Oncology Group (ECOG) Performance Status

Activity Status	Description
**0**	Fully active, able to carry on all predisease performance without restriction
**1**	Restricted in physically strenuous activity but ambulatory and able to carry out performance of a light or sedentary nature, for example, light housework, office work
**2**	Ambulatory and capable of all selfcare but unable to carry out any work activities. Up and about more than 50% of waking hours
**3**	Capable of only limited selfcare, confined to bed or chair more than 50% of waking hours
**4**	Completely disabled. Cannot carry on any selfcare. Totally confined to bed or chair
**5**	Dead

Source: Oken et al. 1982.

Appendix 9. CTCAE 4.03 Definitions for Diarrhea and ILD/Pneumonitis

Diarrhea will be evaluated in this study using the criteria proposed by Common Terminology Criteria for Adverse Events (CTCAE) v4.0 revised: CTCAE 4.03-14 June 2010: Gastrointestinal disorders.

**Gastrointestinal Disorders**					
**Grade**					
**Adverse Event**	**1**	**2**	**3**	**4**	**5**
Diarrhea	Increase of <4 stools per day over baseline; mild increase in ostomy output compared to baseline.	Increase of 4-6 stools per day over baseline; moderate increase in ostomy output compared to baseline	Increase ≥7 stools per day over baseline; incontinence; hospitalization indicated; severe increase in ostomy output compared to baseline; limiting self-care ADL	Life-threatening consequences; urgent intervention indicated	Death
Definition: a disorder characterized by frequent and watery bowel movements.					

Abbreviation: ADL = Activities of Daily Living.

ILD/pneumonitis will be evaluated in this study using the criteria proposed by CTCAE v4.0 revised: CTCAE 4.03–June 14, 2010: Respiratory, thoracic, and mediastinal disorders.

**Respiratory, Thoracic, and Mediastinal Disorders**					
	**Grade**				
**Adverse Event**	**1**	**2**	**3**	**4**	**5**
Pneumonitis/ILD	Asymptomatic; clinical or diagnostic observations only; intervention not indicated	Symptomatic; medical intervention indicated; limiting instrumental ADL	Severe symptoms; limiting self-care ADL; oxygen indicated	Life-threatening respiratory compromise; urgent intervention indicated (e.g., tracheotomy or intubation)	Death
Definition: a disorder characterized by inflammation focally or diffusely affecting the lung parenchyma.					

Abbreviations: ADL = Activities of Daily Living; ILD = interstitial lung disease.

Appendix 10. Protocol JPCE Hepatic Monitoring Tests for Treatment-Emergent Abnormality

Selected tests may be obtained in the event of a treatment-emergent hepatic abnormality and may be required in follow up with patients in consultation with the Lilly clinical research physician.

Hepatic Monitoring Tests

**Hepatic Hematology[a]**	**Haptoglobin[a]**
Hemoglobin	
Hematocrit	**Hepatic Coagulation[a]**
RBC	Prothrombin Time
WBC	Prothrombin Time, INR
Neutrophils, segmented and bands	
Lymphocytes	**Hepatic Serologies[a,b]**
Monocytes	Hepatitis A antibody, total
Eosinophils	Hepatitis A antibody, IgM
Basophils	Hepatitis B surface antigen
Platelets	Hepatitis B surface antibody
	Hepatitis B Core antibody
**Hepatic Chemistry[a]**	Hepatitis C antibody
TBL	Hepatitis E antibody, IgG
Direct bilirubin	Hepatitis E antibody, IgM
Alkaline phosphatase	
ALT	**Anti-nuclear antibody[a]**
AST	
GGT	**Anti-smooth muscle antibody[a]**
CPK	

Abbreviations: ALT = alanine aminotransferase; AST = aspartate aminotransferase; CPK = creatine phosphokinase; GGT = gamma glutamyl transferase; Ig = immunoglobulin; INR = international normalized ratio; RBC = red blood cells; WBC = white blood cells.

a Assayed by Lilly-designated laboratory.

b Reflex/confirmation dependent on regulatory requirements and/or testing availability.

Appendix 11. Protocol Amendment I3Y-MC-JPCE(e) Summary: A Phase 1b Study of Abemaciclib in Combination with Pembrolizumab for Patients with Stage IV Non-Small Cell Lung Cancer or Hormone Receptor-Positive, HER2-Negative Breast Cancer

Overview

Protocol I3Y-MC-JPCE, A Phase 1b Study of Abemaciclib in Combination with Pembrolizumab for Patients with Stage IV Non-Small Cell Lung Cancer or Hormone Receptor-Positive, HER2-Negative Breast Cancer has been amended. The new protocol is indicated by amendment (e) and will be used to conduct the study in place of any preceding version of the protocol.

The overall changes and rationale for the changes made to this protocol are as follows:

- The Rationale for Amendment (e) was added as Section 3.1.5
- Table JPCE.7.2 was updated with dose adjustment criteria for nonhematologic toxicity, ALT/AST increased, and VTEs to align with the current IB
- The abemaciclib dose adjustment criteria for Nonhematologic Toxicity (Section 7.4.1.1.1.1.2) criteria were updated for ALT/AST and added for VTEs to align with the current IB
- The dose adjustment criteria for Hepatic Toxicity (Section 7.4.1.1.1.1.4) were updated related to ALT/AST to align with the current IB
- A cross reference to safety monitoring guidance was added to Interstitial Lung Disease/Pneumonitis (Section 7.4.1.1.1.1.5)
- The dose adjustment criteria for VTEs were added as Section 7.4.1.1.1.1.6 to align with the current IB
- Information for modulators of CYP3A and transporter substrates was updated in the section for Concomitant Therapy (Section 7.7) based on current information
- Safety monitoring language for Special Hepatic Safety Data Collection (Section 9.4.2.1) was updated based on current safety guidance
- Safety monitoring language for VTE (Section 9.4.2.3) was updated to align with the current IB, and
- Section 9.4.2.4 was edited for clarity to include specific ILD/pneumonitis Grades requiring abemaciclib discontinuation, aligning with the IB.

Minor typographical and formatting edits were made throughout the document for clarity and consistency.

Leo Document ID = 2dfa5410-0eaf-4772-a0a6-9ea751de7469

Approver: PPD

Approval Date & Time: 10-Feb-2021 20:04:59 GMT

Signature meaning: Approved

Approver: PPD

Approval Date & Time: 10-Feb-2021 21:46:01 GMT

Signature meaning: Approved
